# Supplementary material for: Impact of COVID-19 vaccination on hospitalization, hospital utilization and expenditure for COVID-19: A retrospective cohort analysis of a South African private health insured population
Source: PLoS One. 2025 Jan 24;20(1):e0317686. doi: 10.1371/journal.pone.0317686 (PMC11760578; doi:10.1371/journal.pone.0317686)
Supplement: Attachment — Submitted filename: PONE-D-24-26424_reviewer_UNN.pdf [file pone.0317686.s004.pdf]

PLOS ONE

Impact of COVID-19 vaccination on COVID-19 hospitalisation, hospital related utilisation and expenditure: Analysis of a South African private health insured population.

--Manuscript Draft--

**Manuscript Number:**	PONE-D-24-26424
**Article Type:**	Research Article
**Full Title:**	Impact of COVID-19 vaccination on COVID-19 hospitalisation, hospital related utilisation and expenditure: Analysis of a South African private health insured population.
**Short Title:**	Impact of COVID-19 vaccination on COVID-19 hospitalisation in a South African private insured population.
**Corresponding Author:**	Geetesh Solanki<br>SAMRC: South African Medical Research Council<br>Cape Town, Western Cape SOUTH AFRICA
**Keywords:**	COVID-19; COVID-19 vaccine; Vaccination; effectiveness, impact assessment; hospitalisation; expenditure; costs; utilisation; hospital length of stay; South Africa, low and middle-income countries; Developing Countries
**Abstract:**	Background:<br>Evidence on the impact of COVID-19 vaccination on COVID-19 hospitalization, utilisation and expenditures in the African context, in privately insured populations and in real world contexts is limited. This study quantifies the impact of COVID-19 vaccination on these outcomes in a large private health insured population cohort over the period of the COVID-19 pandemic in South Africa using real world data.<br>Methods:<br>This cross-sectional analytic study analyses the records of 550,332 individuals belonging to two large health insurances over the period from 1 March 2020 to 31 December 2022. The impact of COVID-19 vaccination was assessed on three outcomes – COVID-19 related hospitalisation risk, utilisation and expenditures. In addition to the variable of interest, COVID-19 vaccination status (not vaccinated, partly vaccinated, fully vaccinated), the analysis incorporated gender, age, chronic conditions, and province as predictor variables. Cox Proportional Hazards model was used to model the impact of vaccination on the risk of hospitalization and Zero-inflated negative binomial models were used to model the impact on utilisation and expenditures.<br>Findings:<br>The COVID-19 related hospitalization rate (events per 1000 individuals per year) was 10.29 for the non-vaccinated, 4.47 for the partly vaccinated and 1.97 for the fully vaccinated. After adjustment, the hospitalisation rate was 94.51% lower for the partly vaccinated (aHR 0.06, 95%CI 0.06, 0.07) and 93.49% lower for the fully vaccinated (aHR 0.07, 95%CI 0.06, 0.07) when compared to the non-vaccinated.<br>The hospital utilization rate (days per 1000 individuals per year) was 93.82 for the non-vaccinated, 44.11 for the partly vaccinated and 20.52 for the fully vaccinated. After adjustment, hospital utilisation was 17.70% (95% CI -24.78%, -9.95%) lower for the partly vaccinated and 20.04% (95% CI -28.26%, -10.88%) lower for the fully-vaccinated compared to the non-vaccinated. The relative risk of zero hospital days was 4.34 (95% CI 4.02, 4.68) for the partly vaccinated increasing to 18.55 (95% CI 17.12, 20.11) for the fully vaccinated compared to the non-vaccinated.<br>The per annum COVID-19 related hospital expenditure for non-vaccinated individuals was USD18, for the partly vaccinated USD6 and for the fully vaccinated USD2. After adjustment for all other factors, hospital expenditure was -32.83% (95% CI -41.06%, -23.44%) lower for the partly vaccinated and -55.29% (95% CI -61.13%, -48.57%) lower for the fully vaccinated when compared to the non-vaccinated. Compared to the non-vaccinated, the relative risk of zero hospital expenditure was 4.38 (95% CI 4.06, 4.73) for the partly vaccinated and 18.61 (95% CI 17.18, 20.16) for the fully vaccinated.<br><br>Conclusions<br>The findings of this study, based on a large cohort of insured individuals followed over

Powered by Editorial Manager® and ProduXion Manager® from Aries Systems Corporation

	the entire period of the pandemic using real world data, confirms that COVID-19 vaccination substantially reduced the risk of hospitalisation, hospital utilisation and expenditures. The findings raise questions as to whether there was a case for applying personal responsibility for COVID-19 vaccination in the study population. The information generated from the study could be used in other assessments of COVID-19 vaccination strategies aimed at better resource allocation, planning and prioritization strategies in the future.
Order of Authors:	Geetesh Solanki
	Susan Cleary
	Francesca Little
Additional Information:	
Question	Response
**Financial Disclosure**<br><br>Enter a financial disclosure statement that describes the sources of funding for the work included in this submission. Review the submission guidelines for detailed requirements. View published research articles from *PLOS ONE* for specific examples.<br><br>This statement is required for submission and **will appear in the published article** if the submission is accepted. Please make sure it is accurate.<br><br>**Funded studies**<br>Enter a statement with the following details:<br>• Initials of the authors who received each award<br>• Grant numbers awarded to each author<br>• The full name of each funder<br>• URL of each funder website<br>• Did the sponsors or funders play any role in the study design, data collection and analysis, decision to publish, or preparation of the manuscript?<br><br>Did you receive funding for this work?	Yes
Please add funding details.<br>as follow-up to "**Financial Disclosure**<br><br>Enter a financial disclosure statement that describes the sources of funding for the work included in this submission. Review the submission guidelines for detailed requirements. View published research	Geetesh Solanki is employed on a contractual basis by the South African Medical Research Council and NMG Consultants and Actuaries, an independent consulting firm providing consulting and actuarial services to South African private health insurance funds.<br><br>NMG provided access to the data used for the study but did not have any other role or involvement in the study and Geetesh Solanki's commercial affiliation to NMG therefore played no role in influencing the study.

articles from *PLOS ONE* for specific examples.<br><br>This statement is required for submission and **will appear in the published article** if the submission is accepted. Please make sure it is accurate.<br><br>**Funded studies**<br>Enter a statement with the following details:<br>• Initials of the authors who received each award<br>• Grant numbers awarded to each author<br>• The full name of each funder<br>• URL of each funder website<br>• Did the sponsors or funders play any role in the study design, data collection and analysis, decision to publish, or preparation of the manuscript?<br><br>Did you receive funding for this work?"	The South African Medical Research Council provided support in the form of salaries for authors Geetesh Solanki, and the University of Cape Town for Francesca Little and Susan Cleary. None of these institutions had any additional role in the study design, data collection and analysis, decision to publish, or preparation of the manuscript. The specific roles of these authors are articulated in the 'author contributions' section."
Please select the country of your main research funder (please select carefully as in some cases this is used in fee calculation).<br>as follow-up to "**Financial Disclosure**<br><br>Enter a financial disclosure statement that describes the sources of funding for the work included in this submission. Review the submission guidelines for detailed requirements. View published research articles from *PLOS ONE* for specific examples.<br><br>This statement is required for submission and **will appear in the published article** if the submission is accepted. Please make sure it is accurate.	SOUTH AFRICA - ZA

**Funded studies**<br>Enter a statement with the following details:<br>• Initials of the authors who received each award<br>• Grant numbers awarded to each author<br>• The full name of each funder<br>• URL of each funder website<br>• Did the sponsors or funders play any role in the study design, data collection and analysis, decision to publish, or preparation of the manuscript?<br><br>Did you receive funding for this work?"	
**Competing Interests**<br><br>Use the instructions below to enter a competing interest statement for this submission. On behalf of all authors, disclose any competing interests that could be perceived to bias this work—acknowledging all financial support and any other relevant financial or non-financial competing interests.<br><br>This statement is required for submission and **will appear in the published article** if the submission is accepted. Please make sure it is accurate and that any funding sources listed in your Funding Information later in the submission form are also declared in your Financial Disclosure statement.<br><br>View published research articles from *PLOS ONE* for specific examples.	Geetesh Solanki is employed on a contractual basis by NMG Consultants and Actuaries, an independent consulting firm providing consulting and actuarial services to South African private health insurance funds.<br><br>NMG provided access to the data used for this study but did not have any additional role in the study design, data collection and analysis, decision to publish, or preparation of the manuscript. As such, there were no conflicts of interest in the conduct of the study.

**NO authors have competing interests**<br><br>Enter: *The authors have declared that no competing interests exist.*<br><br>**Authors with competing interests**<br><br>Enter competing interest details beginning with this statement:<br><br>*I have read the journal's policy and the authors of this manuscript have the following competing interests: [insert competing interests here]*<br><br>* typeset	
**Ethics Statement**<br><br>Enter an ethics statement for this submission. This statement is required if the study involved:<br><br>• Human participants<br>• Human specimens or tissue<br>• Vertebrate animals or cephalopods<br>• Vertebrate embryos or tissues<br>• Field research<br><br>Write "N/A" if the submission does not require an ethics statement.<br><br>General guidance is provided below. Consult the submission guidelines for detailed instructions. **Make sure that all information entered here is included in the Methods section of the manuscript.**	Ethics approval for the use of the secondary database used to carry out this study was granted by the Faculty of Health Sciences Human Research Ethics Committee of the University of Cape Town – approval number HREC Ref: 606/2023. No individual consent was required as the research was based on anonymised secondary claims data.<br>Data for the study were made available as part of NMG Consultants and Actuaries (NMG) commitment to support research initiatives with broader public health significance. The data were accessed in terms of and under the conditions set out in the agreement between NMG and the client schemes and between NMG and the researcher. The terms set out in these agreements ensured that the access to and use of the data is in full compliance with the requirements of the Protection of Personal Information (PoPI) Act in South Africa[42]. All the data were provided in a de-identified format and aggregated at an individual level and the research team had no access to information that would enable the identification of any individual. All findings are presented at an aggregate level and no confidential member, health care provider or scheme information is disclosed. Individual consent for the data was therefore not required.

Powered by Editorial Manager® and ProduXion Manager® from Aries Systems Corporation

**Format for specific study types**<br><br>**Human Subject Research (involving human participants and/or tissue)**<br>• Give the name of the institutional review board or ethics committee that approved the study<br>• Include the approval number and/or a statement indicating approval of this research<br>• Indicate the form of consent obtained (written/oral) or the reason that consent was not obtained (e.g. the data were analyzed anonymously)<br><br>**Animal Research (involving vertebrate animals, embryos or tissues)**<br>• Provide the name of the Institutional Animal Care and Use Committee (IACUC) or other relevant ethics board that reviewed the study protocol, and indicate whether they approved this research or granted a formal waiver of ethical approval<br>• Include an approval number if one was obtained<br>• If the study involved *non-human primates*, add *additional details* about animal welfare and steps taken to ameliorate suffering<br>• If anesthesia, euthanasia, or any kind of animal sacrifice is part of the study, include briefly which substances and/or methods were applied<br><br>**Field Research**<br><br>Include the following details if this study involves the collection of plant, animal, or other materials from a natural setting:<br>• Field permit number<br>• Name of the institution or relevant body that granted permission	
**Data Availability**<br><br>Authors are required to make all data underlying the findings described fully available, without restriction, and from the time of publication. PLOS allows rare exceptions to address legal and ethical concerns. See the PLOS Data Policy and FAQ for detailed information.	No - some restrictions will apply

Powered by Editorial Manager® and ProduXion Manager® from Aries Systems Corporation

A Data Availability Statement describing where the data can be found is required at submission. Your answers to this question constitute the Data Availability Statement and **will be published in the article**, if accepted.<br><br>**Important:** Stating 'data available on request from the author' is not sufficient. If your data are only available upon request, select 'No' for the first question and explain your exceptional situation in the text box.<br><br>Do the authors confirm that all data underlying the findings described in their manuscript are fully available without restriction?	
**Describe where the data may be found in full sentences. If you are copying our sample text, replace any instances of XXX with the appropriate details.**<br><br>• If the data are **held or will be held in a public repository**, include URLs, accession numbers or DOIs. If this information will only be available after acceptance, indicate this by ticking the box below. For example: *All XXX files are available from the XXX database (accession number(s) XXX, XXX.).*<br>• If the data are all contained **within the manuscript and/or Supporting Information files**, enter the following: *All relevant data are within the manuscript and its Supporting Information files.*<br>• If neither of these applies but you are able to provide **details of access elsewhere**, with or without limitations, please do so. For example:<br><br>*Data cannot be shared publicly because of [XXX]. Data are available from the XXX Institutional Data Access / Ethics Committee (contact via XXX) for researchers who meet the criteria for access to confidential data.*<br><br>*The data underlying the results presented in the study are available from (include the name of the third party*	The data used for this study are not proprietary and will be made available by the SAMRC to individuals who want to access. The criteria for access would broadly incorporate requests from individuals with credible academic/research credentials. This does not alter our adherence to PLOS ONE policies on sharing data and materials. Requests for access to the data should be directed to: Mr Ishen Seocharan, SAMRC Email: Ishen. Seocharan@mrc.ac.za

and contact information or URL).<br>• This text is appropriate if the data are owned by a third party and authors do not have permission to share the data.<br><br>* typeset	
Additional data availability information:	Tick here if the URLs/accession numbers/DOIs will be available only after acceptance of the manuscript for publication so that we can ensure their inclusion before publication.

Manuscript

Click here to access/download;Manuscript;Impact COVID Vac COVIDHosp ver 3 July24.docx

Impact of COVID-19 vaccination on COVID-19 hospitalisation, hospital related utilisation and expenditure: Analysis of a South African private health insured population.

Geetesh Solanki[1, 2, 3], Susan Cleary[2,] Francesca Little[4],

Author affiliations

[1] Health Systems Research Unit, South African Medical Research Council

[2] Health Economics Unit, University of Cape Town, Cape Town, South Africa

[3] NMG Consultants and Actuaries, Cape Town, South Africa

[4] Department of Statistical Sciences, University of Cape Town

Corresponding author: Geetesh Solanki – geetesh.solanki@mrc.ac.za

// Abstract

Background:

Evidence on the impact of COVID-19 vaccination on COVID-19 hospitalization, utilisation and expenditures in the African context, in privately insured populations and in real world contexts is limited. This study quantifies the impact of COVID-19 vaccination on these outcomes in a large private health insured population cohort over the period of the COVID-19 pandemic in South Africa using real world data.

Methods:

This cross-sectional analytic study analyses the records of 550,332 individuals belonging to two large health insurances over the period from 1 March 2020 to 31 December 2022. The impact of COVID-19 vaccination was assessed on three outcomes – COVID-19 related hospitalisation risk, utilisation and expenditures. In addition to the variable of interest, COVID-19 vaccination status (not vaccinated, partly vaccinated, fully vaccinated), the analysis incorporated gender, age, chronic conditions, and province as predictor variables. Cox Proportional Hazards model was used to model the impact of vaccination on the risk of hospitalization and Zero-inflated negative binomial models were used to model the impact on utilisation and expenditures.

Findings:

The COVID-19 related hospitalization rate (events per 1000 individuals per year) was 10.29 for the non-vaccinated, 4.47 for the partly vaccinated and 1.97 for the fully vaccinated. After adjustment, the hospitalisation rate was 94.51% lower for the partly vaccinated (aHR 0.06, 95%CI 0.06, 0.07) and 93.49% lower for the fully vaccinated (aHR 0.07, 95%CI 0.06, 0.07) when compared to the non-vaccinated.

The hospital utilization rate (days per 1000 individuals per year) was 93.82 for the non-vaccinated, 44.11 for the partly vaccinated and 20.52 for the fully vaccinated. After adjustment, hospital utilisation was 17.70% (95% CI -24.78%, -9.95%) lower for the partly vaccinated and 20.04% (95% CI -28.26%, -10.88%) lower for the fully- vaccinated compared to the non-vaccinated. The relative risk of zero hospital days was 4.34 (95% CI 4.02, 4.68) for the partly vaccinated increasing to 18.55 (95% CI 17.12, 20.11) for the fully vaccinated compared to the non-vaccinated.

The per annum COVID-19 related hospital expenditure for non-vaccinated individuals was USD18, for the partly vaccinated USD6 and for the fully vaccinated USD2. After adjustment for all other factors, hospital expenditure was -32.83% (95% CI -41.06%, -23.44%) lower for the partly vaccinated and -55.29% (95% CI -61.13%, -48.57%) lower for the fully vaccinated when compared to the non-vaccinated. Compared to the non-vaccinated, the relative risk of zero hospital expenditure was 4.38 (95% CI 4.06, 4.73) for the partly vaccinated and 18.61 (95% CI 17.18, 20.16) for the fully vaccinated.

Conclusions:

The findings of this study, based on a large cohort of insured individuals followed over the entire period of the pandemic using real world data, confirms that COVID-19 vaccination substantially reduced the risk of hospitalisation, hospital utilisation and expenditures. The findings raise questions as to whether there was a case for applying personal responsibility for COVID-19 vaccination in the study population. The information generated from the study could be used in other assessments of COVID-19 vaccination strategies aimed at better resource allocation, planning and prioritization strategies in the future.

Introduction

COVID-19 was declared a Public Health Emergency of International Concern on 30 January 2020, and a pandemic on 11 March 2020 by the World Health Organization (WHO) [1, 2], triggering an international response to develop a preventive COVID-19 vaccine with four vaccine candidates entering human evaluation by March 2020[3]. South Africa commenced its national COVID-19 vaccination program on 17 February 2021[4]. By 31 December 2023, based on the World Health Organisation (WHO) COVID-19 dashboard, South Africa had a total of 4.1 million COVID-19 confirmed cases; 70 vaccination doses had been administered per 100 population, 41 persons per 100 population had completed primary series vaccination, and 7 persons per 100 population had completed the primary vaccination series and at least one additional dose or booster vaccination [5]. The COVID-19 reported deaths on the WHO dashboard are however likely to be under-reported, with studies showing that while there were 93,186 COVID-19 reported deaths over the period from December 2019 to January 2022, there were 295,135 excess natural deaths (most likely due to COVID-19) [6].

The emergence and development of the COVID-19 pandemic provided the impetus for research initially focused on identifying the risk factors for COVID-19 related morbidity and mortality. There is now a considerable body of evidence highlighting the importance of comorbidities, age and obesity as important risk factors[7-9] . Following the development, approval and application of various COVID-19 vaccines, the research focus shifted towards examining the effectiveness of the vaccine in reducing the rates of infections, severity, hospitalization, and mortality. While varying in the population(s) studied and methodologies used, there is a substantial evidence including systematic reviews and/or meta-analyses of "real world" studies[10, 11] reporting on the type and dose of vaccines[11], specific risk groups[12, 13], examining duration and impact of the vaccine[14] and the impact on long COVID[13].

A review of the literature on the impact of COVID-19 vaccination in African and sub-Saharan regions revealed clinical trials examining the efficacy of specific COVID-19 vaccines against specific COVID-19 variants [15-18], studies relying on modelling the impact and cost-effectiveness of the COVID-19 vaccination[19-21], studies examining equity issues related to COVID-19 vaccine access and coverage[22-24], and studies examining the effectiveness of COVID-19 vaccination in "real world" situations[17, 25]. Overall, while there is now a substantial global body of evidence on the impact of COVID-19 vaccine globally, research in African and sub-Saharan regions is more limited. Societies in countries in these regions are highly segmented, with high levels of inequity and access to health services, differences in levels of vaccine awareness, and in vaccine coverage. The overall impact of vaccination across the different strata in these countries can therefore be expected to be different and further research is required to elucidate the differences.

Evidence on privately insured populations in these regions (and globally) is also limited. With 40% of health services in the sub Saharan African region being sourced from private providers, the for-profit private sector is an important provider of health services [26]. South Africa has a highly fragmented two-tiered health system with substantial disparities in access, facilities and spending between the government-funded public health system and the private health system. Around 18% of the total South African population is covered by private health insurance[27] which is the dominant funding mechanism for the private health system. The differences in underlying demographic profiles, socio-economic differentials, and access to health services of the public and private sector-dependent populations can be expected to result in differences in their COVID-19 related outcomes.

Relying largely on a fee-for-service model for provider payment and established clinical coding system, the private health insurance model generates substantial data, enabling analyses of utilization, risk and expenditure of beneficiaries which can provide valuable insights. In South Africa, this approach has been taken to investigate for example, use of antibiotics [28], take-up of influenza vaccines[29], caesarean

section rates [30] and risk factors for COVID-19 related outcomes[31]. To date, only one study, focused on examining differences in mortality amongst individuals admitted to hospital with COVID-19 during the first and second waves in South Africa, included data from the private sector [32]. The study found a lower overall case-fatality risk in the private sector than in the public sector. Globally, apart from studies reporting on Medicare/Medicaid members, reports on the impact of COVID-19 vaccination on COVID-19 related outcomes in private sector-dependent populations are limited and research in this area can provide new and different insights on the impact of COVID-19 and COVID-19 vaccination.

The aim of this paper is to assess and quantify the impact of COVID-19 vaccination on COVID-19 hospitalization, hospital related utilization and expenditure in a private health insured population cohort over the entire period of the COVID-19 pandemic in South Africa using Real World Data (RWD). The results from this study contribute to addressing the gap in knowledge on the aggregate “real life” impact of COVID-19 vaccination in a population cohort over the entire period of the pandemic; in insured populations; and in populations in Sub Saharan Africa; and it will generate information to assess the effectiveness, cost and cost effectiveness of COVID-19 vaccination in preventing COVID-19 related morbidity.

Methods

Study design

This is a cross sectional analytic study based on the analysis of secondary anonymised demographic and medical claims records held in the data warehouse of NMG Consultants and Actuaries (NMG), an independent consulting firm providing consulting and actuarial services to South African private health insurance funds that provide financial cover for medical expenses for members who pay a monthly contribution for this cover.

Study population

The study population consisted of 550,332 individuals belonging to 2 large NMG client funds representing around 5% of South Africa’s privately insured population. The average family size, average contributions and health care expenditure of the study population were compared with those of the overall health insured population and found to be broadly comparable, thus ensuring that the findings of this study are generalisable to the broader South African population with private health insurance (Table S1)[33].

Data Sources

Data for the period from 1 March 2020 to 31 December 2022 were extracted from the NMG data warehouse on 6 November 2023. The data extract covered the period of the four waves of the COVID-19 pandemic in South Africa with the first wave starting on 5 March 2020[34] (first reported case of COVID-19 in South Africa) and the fourth wave “ending" with the lifting of restrictions related to the wave on 30 December 2022[35].

For each insured individual, the data contains the following variables: unique individual identifier, date of birth, gender, and province. For each claim submitted to the administrator for reimbursement of services rendered or items dispensed to an insured individual, the data contains the following variables: a unique individual identifier; dates for commencement and completion of the service; a code and description for each service rendered/item dispensed; an ICD-10 (10th revision of the International Statistical Classification of Diseases and Related Health Problems) code for the diagnosis of the condition being treated; a Current Procedural Terminology (CPT) code for the procedure carried out; a National Pharmaceutical Product Index (NAPPI) code for any surgical, medical or consumable item dispensed; and

the amount being claimed (amount charged by provider) and the expenditure incurred (amount paid by insurer).

Data extraction and classification

The approach used to extract and classify the data is schematically summarized in Figure 1. From all the data for the period, a 3-step approach was used to extract and compile the data into various datasets.

Figure 1. Approach to data extraction

For the first step, three data files were created. The first "demographic" file consisted of a record per individual who belonged to either of the two medical schemes at any time over the study period. Each record captured the demographic information for the individual (unique study ID, date of joining and exiting the fund, study period, age group, gender, geographical region, and co-morbidities). Although the overall study period extended from 1 March 2020 to 31 December 2022 (34 months or 2.83 years), there are some individuals who joined the health insurance scheme after 1 March 2020 and some who left before 31 December 2022. For these individuals the study period was limited to the period during

which they belonged to the fund within the broader study period. For the comorbidity variable, the 26 chronic disease list conditions listed by the South African Council for Medical Schemes as part of the Prescribed Minimum Benefits were considered for defining the comorbidity status[36]. The individuals with these comorbidities were identified using the South African Council for Medical Scheme Guidelines for identifying members with medical conditions using claims records [37].

The second "COVID-19 vaccination status" data file consisted of a record for every change in the vaccination status of an individual (unique study ID, date of change of vaccination status, vaccination status). The South African government authorized the Pfizer BioNtech and Johnson and Johnson (J&J) vaccines. For the purposes of this study, in line with approaches used in studies elsewhere[38] and the categorization used in South Africa[39], individuals were categorized as fully vaccinated 28 days after receiving the J&J vaccine or 14 days after receipt of the second dose of Pfizer BioNtech. Individuals receiving further booster vaccinations remained in the fully vaccinated category. Individuals were categorized as partially vaccinated the day after the first vaccine dose (J&J or Pfizer BioNtech) until meeting the criteria for complete vaccination. Individuals were recorded as non-vaccinated if they had received no vaccinations. Individuals received their vaccinations at various points in time and their vaccination status therefore changed over time. For those who were hospitalized, the vaccination status at the time of admission for each hospital event was recorded. For those who were not hospitalized, the vaccination status at the end point of the study was recorded.

The "COVID-19 hospital admission" data file consisted of a record for each COVID-19 related hospital admission (unique study ID, date of admission, date of discharge, length of stay and total hospital and related expenditure). These admissions were identified using admission ICD codes U07.1 or U07.2[40]. Individuals with multiple admissions had separate records for each admission.

For the second step, two separate datasets were created using information from the three files described above. The first dataset, created to analyze the risk of hospitalization, consisted of "time stamped" records for every hospitalisation or change of vaccination status event, together with records for all individuals who had no events. Each record included demographic information of the individual, hospitilisation and vaccination status indicators and the time stamp for the event.

The second dataset, created to analyse hospital utilisation and expenditures, consisted of a record for every individual who had no hospital event over the period and a record for every hospital event for individuals hospitalised over the period. Each record included demographic information of the individual, hospitalisation and vaccination status indicators, and number of hospital days and expenditure per hospital event (zero if no hospital event).

Statistical analysis

The impact of COVID-19 vaccination was assessed on three COVID-19 related outcomes – the risk of hospitalization (individual having a hospital event or not), extent of hospital utilisation (number of days in hospital) and extent of hospital expenditures (total hospital and related expenditures in 2022 USD terms).

In addition to the variable of interest, COVID-19 vaccination status (not vaccinated, partly vaccinated, fully vaccinated), the analysis incorporated gender (male, female), age (less than 18, between 18 and 25, between 25 and 40, between 40 and 65 and greater than 65 years), chronic condition (yes, no) and province (Eastern Cape, Free State, Gauteng, KwaZulu-Natal, Limpopo, Mpumalanga, North West, Northern Cape, Western Cape) as predictor variables.

For the impact of vaccination on risk of hospitalization analysis, summary statistics reported include frequencies and percentages for the number of individuals, and hospitalization frequencies and rates

(number of hospital events per 1000 individuals per annum). For the multivariable analysis, survival analysis was used to model the impact of vaccination on the risk of hospitalization to account for censoring due to no hospitalization during the observation period. A Cox Proportional Hazards model was used to estimate the relative hazard of hospitalization. Time-varying vaccination status was incorporated through repeated measures per individual relating to the periods with different vaccination statuses. The large data set did not allow for estimation of shared frailty models. The repeated observations per individual (individuals with multiple vaccination periods and multiple hospital events) was taken into consideration by incorporating the data clustering option by individual in the modelling and hence the estimation of robust standard errors. The proportional hazards assumption was assessed through Kaplan-Meier curves and found to be justifiable. Unadjusted Hazard Ratios (UHR) and Adjusted Hazard Ratios (AHR) with 95% confidence intervals are presented.

For the impact of vaccination on hospital utilisation analysis, the summary statistics included the total number of days in hospital, number of days per hospitalization event and the number of hospital days per 1000 individuals per annum. For the expenditure analysis, all the expenditures in South African Rands (ZAR) were converted to US Dollar (USD) value using the December USD/ZAR exchange rate for the given year and then inflated/deflated to be expressed in 2022 values using the South African Consumer Price Index[41]. The summary statistics include the total expenditure in millions (USD), expenditure per hospitalization event and the expenditure per individual per annum.

For the multivariable analysis of the impact of vaccination on utilisation and expenditure, zero-inflated negative binomial models were used as the exploratory analyses indicated a highly skewed distribution not only because of the zeros, but also a skewed distribution on the non-zero values. The repeated observations per individual (individuals with multiple hospital events) would ideally have been dealt with by the inclusion of random effects in the model. However, as STATA does not allow for the

incorporation of random effects in zero inflated models, the data clustering option by subject was used to derive robust standard errors.

The zero-inflated negative binomial model produces two sets of outputs: the count component and the zero-inflated component. The count component examines the effects of predictor variables on the expected non-zero count of the outcome variable, while the zero-inflated component explores factors influencing the probability of the presence of excess zeros. The unadjusted and adjusted estimated coefficients (e.g. beta1) from the count component of the model output were expressed as percentage increase or decrease in the number of average days/ expenditures depending on whether the coefficient is positive or negative using the formula, (exp(beta1)-1) x100. The unadjusted and adjusted estimated coefficients from the zero inflated component were expressed as the risk ratio for zero hospital days compared to the reference group using the formula exp(beta). STATA/SE 16.1 was used for all the analyses.

Ethical considerations

Data for the study were made available as part of NMG Consultants and Actuaries (NMG) commitment to support research initiatives with broader public health significance. The company does not advise its clients on the clinical treatment of its members. The data were accessed in terms of and under the conditions set out in the agreement between NMG and the client schemes and between NMG and the researcher. The terms set out in these agreements ensured that the access to and use of the data is in full compliance with the requirements of the Protection of Personal Information (PoPI) Act in South Africa[42].

All the data were provided in a de-identified format and aggregated at an individual level and the research team had no access to information that would enable the identification of any individual. All

findings are presented at an aggregate level and no confidential member, health care provider or scheme information is disclosed. Ethics approval for the use of the database to carry out this study was granted by the Faculty of Health Sciences Human Research Ethics Committee (project registration number HREC Ref: 606/2023).

Results

Sample description

The total dataset comprised the information on 550,332 individuals who belonged to the two health insurance funds between 1 March 2020 to 31 December 2022. At the end of the study period, 62.1% were not vaccinated, 12.2% were partly vaccinated, and 25.7% were fully vaccinated. Of all the individuals, 53.6% were female, 34.4% were less than 18 years old, 23.3% had one or more chronic conditions, and the largest proportion (45.4%) of individuals were from the Gauteng province (Table 1).

Hospitalization Risk

The overall COVID-19 related hospitalization rate (events per 1000 individuals per year) for the study population was 7.45 (Table 1). The hospitalization rate was 10.29 for the non-vaccinated, 4.47 for the partly vaccinated and 1.97 for the fully vaccinated. Across the other predictor variables, males (7.56), individuals aged 65 and above (25.62), those with chronic conditions (20.12) and those from the Western Cape (9.02) had the highest hospitalization rates.

In multivariable analysis, after adjustment for all other factors, the risk of hospitalisation was 94.51% lower for the partly vaccinated (aHR 0.06, 95%CI 0.06, 0.07) and 93.49% lower for the fully vaccinated (aHR 0.07, 95%CI 0.06, 0.07) when compared with non-vaccinated. The risk of hospitalization was higher for males (aHR 1.17, 95%CI 1.13, 1.12), those aged 65 and above (aHR 17.27, 95% CI 15.78,

18.89), and those with chronic conditions (aHR 3.33, 95%CI 3.18, 3.50). Individuals from all the other provinces had a higher risk of utilization than individuals from the Eastern Cape, the reference group.

Table 1. Sample description, and univariate and multivariate analysis of impact of COVID-19 vaccination on COVID-19 related hospitalization

Variable	Number of individuals		# of Hosp Events		Hazard Ratio for Hospitilisation	
	N	%	N	Events/1000 Individuals/ per annum**	UHR (95% CI)	AHR (95% CI)
**Vaccination Status***						
Not Vaccinated	341,972	62.1%	9,972	10.29	Reference	Reference
Part Vaccinated	66,885	12.2%	848	4.47	0.17 (0.15, 0.18)	0.06 (0.06, 0.07)
Fully vaccinated	141,475	25.7%	791	1.97	0.20 (0.19, 0.22)	0.07 (0.06, 0.07)
**Gender**						
Female	295,116	53.6%	6,145	7.35	Reference	Reference
Male	255,216	46.4%	5,466	7.56	1.05 (1.01, 1.09)	1.17 (1.13, 1.22)
**Age**						
Less than 18	189,405	34.4%	927	1.73	Reference	Reference
Between 18-25	40,190	7.3%	271	2.38	1.33 (1.16, 1.53)	1.99 (1.73, 2.29)
Between 25-40	145,004	26.3%	2,407	5.86	3.49 (3.23, 3.78)	5.04 (4.66, 5.46)
Between 40-65	140,255	25.5%	5,431	13.67	7.64 (7.11, 8.22)	10.32 (9.55, 11.14)
Greater than 65	35,478	6.4%	2,575	25.62	13.93 (12.84, 15.11)	17.27 (15.78, 18.89)
**Chronic Condition?**						
No	422,116	76.7%	4,303	3.60	Reference	Reference
Yes	128,216	23.3%	7,308	20.12	4.94 (4.74, 5.15)	3.33 (3.18, 3.50)
**Province**						
Eastern Cape	40,694	7.4%	827	7.17	Reference	Reference
Free State	22,347	4.1%	461	7.28	1.08 (0.95, 1.22)	1.26 (1.12, 1.43)
Gauteng	249,650	45.4%	4,812	6.80	0.95 (0.88, 1.03)	1.33 (1.22, 1.44)
KwaZulu-Natal	85,572	15.5%	2,084	8.60	1.21 (1.11, 1.32)	1.30 (1.19, 1.42)
Limpopo	19,410	3.5%	271	4.93	0.74 (0.64, 0.85)	1.04 (0.90, 1.21)
Mpumalanga	18,514	3.4%	320	6.10	0.90 (0.78, 1.03)	1.02 (0.88, 1.17)
North West	19,064	3.5%	419	7.76	1.14 (1.00, 1.30)	1.33 (1.17, 1.52)
Northern Cape	10,002	1.8%	242	8.54	1.27 (1.09, 1.48)	1.42 (1.22, 1.66)
Western Cape	85,079	15.5%	2,175	9.02	1.26 (1.15, 1.37)	1.32 (1.21, 1.44)
**Total**	550,332	100.0%	11,611	7.45		

Vaccination Status*: Status at end of study for number of individuals, status at time of hospital event for hospital events.

Events/1000 Individuals/per annum**: Total events/ number of individuals/study period (2.83 years) *1000

Hospitalization Utilisation

The overall COVID-19 related hospital utilization rate was 69.22 days per 1000 individuals per year with an average length of stay of 9.30 days (Table 2). The utilization rate was 93.82 for the non-vaccinated, 44.11 for the partly vaccinated and 20.52 for the fully vaccinated. Across the other predictor variables,

males (73.78), individuals aged 65 and above (322.4), those with chronic conditions (214.87) and those from the Western Cape (86.56) had the highest hospitalization rates.

In multivariable analysis, after adjustment for all other factors, the results from the count component of the model indicated that compared with the non-vaccinated, hospital utilisation was 17.70% (95% CI -24.78%, -9.95%) lower for the partly vaccinated and 20.04% (95% CI -28.26%, -10.88%) lower for the fully- vaccinated. The zero inflated component results indicated that compared to the non-vaccinated, the relative risk of zero hospital days was 4.34 (95% CI 4.02, 4.68) for the partly vaccinated increasing to 18.55 (95% CI 17.12, 20.11) for the fully vaccinated. Across the other predictor variables, the results from the count component indicated that hospital utilisation was higher by 10.87% (95% CI 5.38%, 16.64%) for males compared to females, 198.39% (95% CI 156.51%, 247.10%) for those greater than 65-year-old compared to under 18's, 24.82% (95% CI 17.99%, 32.05%) for those with chronic conditions compared to those without. The zero inflated component results indicated that that the relative risk of zero hospital days was 0.87 (95% CI 0.84, 0.91) for males compared to females, 0.06 (95% CI 0.06, 0.07) for those greater than 65-year-old compared to under 18's, 0.26 (95% CI 0.25, 0.27) for those with a chronic condition compared to those without and 0.74 (95% CI 0.68, 0.82) for those from the Western Cape relative to the Eastern Cape.

Table 2. Sample description, and univariate and multivariate analysis of impact of COVID-19 vaccination on COVID-19 hospital utilisation

	Hospital Days			% Difference in Hospital Days vs Reference Group***		Risk Ratio of Zero Hospital Day vs. Reference Group****	
	Number of days	Days/ Event	Days/ 1000 individuals/ year**	Unadjusted	Adjusted	Unadjusted	Adjusted
**Vaccination Status***							
Not Vaccinated	91,738	9.19	93.82	Reference	Reference	Reference	Reference
Part Vaccinated	7,976	9.41	44.11	-0.54% (-9.26%, 9.02%)	-17.70% (-24.78%, -9.95%)	2.29 (2.12, 2.47)	4.34 (4.02, 4.68)
Fully vaccinated	8,219	10.39	20.52	0.17% (-10.39%, 11.98%)	-20.04% (-28.26%, -10.88%)	5.19 (4.80, 5.62)	18.55 (17.12, 20.11)
**Gender**							
Female	54,582	8.88	65.28	Reference	Reference	Reference	Reference
Male	53,351	9.76	73.78	11.90% (6.27%, 17.83%)	10.87% (5.38%, 16.64%)	0.98 (0.94, 1.02)	0.87 (0.84, 0.91)
**Age**							
Less than 18	3,870	4.17	7.21	Reference	Reference	Reference	Reference
Between 18-25	1,479	5.46	12.99	39.54% (6.54%, 82.77%)	38.10% (7.47%, 77.45%)	0.77 (0.67, 0.90)	0.57 (0.49, 0.66)
Between 25-40	15,518	6.45	37.77	69.91% (45.38%, 98.58%)	65.55% (42.17%, 92.77%)	0.32 (0.29, 0.35)	0.25 (0.23, 0.27)
Between 40-65	54,658	10.06	137.54	176.56% (138.65%, 220.50%)	152.54% (118.19%, 192.29%)	0.14 (0.13, 0.15)	0.12 (0.11, 0.13)
Greater than 65	32,408	12.59	322.40	228.82% (182.87%, 282.24%)	198.39% (156.51%, 247.10%)	0.07 (0.07, 0.08)	0.06 (0.06, 0.07)
**Chronic Condition?**							
No	29,875	6.94	24.98	Reference	Reference	Reference	Reference
Yes	78,058	10.68	214.87	55.32% (47.18%, 63.91%)	24.82% (17.99%, 32.05%)	0.18 (0.17, 0.19)	0.26 (0.25, 0.27)
**Province**							
Eastern Cape	8,175	9.89	70.90	Reference	Reference	Reference	Reference
Free State	4,449	9.65	70.27	-2.58% (-16.17%, 13.21%)	-0.18% (-14.02%, 15.89%)	0.98 (0.86, 1.11)	0.84 (0.74, 0.96)
Gauteng	46,851	9.74	66.24	-6.42% (-14.71%, 2.68%)	-0.88% (-9.52%, 8.60%)	1.05 (0.96, 1.13)	0.76 (0.70, 0.83)
KwaZulu-Natal	17,369	8.33	71.64	-19.14% (-27.01%, -10.42%)	-11.32% (-19.85%, -1.89%)	0.81 (0.74, 0.88)	0.74 (0.67, 0.81)
Limpopo	1,981	7.31	36.02	-28.91% (-42.39%, -12.27%)	-18.05% (-31.78%, -1.55%)	1.40 (1.21, 1.63)	0.97 (0.83, 1.13)
Mpumalanga	2,816	8.80	53.68	-12.15% (-27.30%, 6.15%)	-5.67% (-20.69%, 12.21%)	1.16 (1.01, 1.33)	0.98 (0.85, 1.13)
North West	3,441	8.21	63.70	-18.76% (-29.19%, -6.78%)	-16.46% (-26.91%, -4.53%)	0.90 (0.79, 1.03)	0.75 (0.66, 0.86)
Northern Cape	1,986	8.21	70.08	-18.82% (-32.45%, -2.45%)	-16.05% (-30.38%, 1.24%)	0.81 (0.70, 0.95)	0.70 (0.60, 0.83)
Western Cape	20,865	9.59	86.56	-6.88% (-16.40%, 3.72%)	-8.83% (-17.66%, 0.95%)	0.78 (0.72, 0.85)	0.74 (0.68, 0.82)
**Total**	107,933	9.30	69.22				

Vaccination Status*: Status as time of hospital event if hospitilised, and as end of study period if never hospitilised during study period

Days/1000 Individuals/per annum**: Total days/ number of individuals/study period (2.83 years) *1000

% Difference in hospital days***: Exponent of estimated coefficents (beta1) from count component output from negative binonmial model - 1 X 100: (exp(beta1)-1)x100.

Risk Ratio for zero hospital days****: Exponent of estimated coefficents (beta1) from zero inflated component output from negative binonmial model : exp(beta1)

Hospitalization Expenditures

The overall COVID-19 related hospital expenditure (expenditure per individual per year) for the study population was USD12 with an average expenditure per stay of USD1,664 (Table 3). The expenditure for was USD18 per annum non-vaccinated individuals, USD6 for partly vaccinated individuals, and USD2 for fully vaccinated individuals. Across the other predictor variables, males (USD13), individuals aged 65 and above (USD110), those with chronic conditions (USD38) and those from the Western Cape and Kwazulu-Natal (USD16) had the highest hospitalization expenditures.

In multivariable analysis, after adjustment for all other factors, the results from the count component of the model indicated that hospital expenditure was -32.83% (95% CI -41.06%, -23.44%) lower for the

partly vaccinated and -55.29% (95% CI -61.13%, -48.57%) lower for the fully vaccinated when compared to the non-vaccinated. Hospital expenditures were higher by 9.62% (95% CI 0.37%, 18.57%) for males compared to females, 259.12% (95%CI 195.45%, 337.44%) for those aged 65 and above compared to under 18's and 19.43% (95%CI 8.29%, 31.71%) for those with chronic conditions compared to those without. The zero inflated component results indicated that compared to the non-vaccinated, the relative risk of zero hospital expenditure was 4.38 (95% CI 4.06, 4.73) for the partly vaccinated and 18.61 (95% CI 17.18, 20.16) for the fully vaccinated compared to the non-vaccinated. Across the other predictor variables, the results from the count component indicated that hospital expenditure was 9.75% (95% CI 0.86%, 19.43%) higher for males compared to females, 257.83% (95% CI 194.09%, 335.40%) higher for those aged greater than 65-year-old compared to the under 18's, 19.44% (8.31%, 31.72%) for those with chronic conditions compared to those without and 38.59% (95% CI 17.77%, 63.10%) for those from the Western Cape. The zero inflated component results indicated that the relative risk of zero hospital expenditure was 0.86 (95% CI 0.83, 0.90) for males compared to females, 0.06 (95% CI 0.05, 0.06) for those aged greater than 65-year-old compared to the under 18's, 0.25 (0.24, 0.27) for those with chronic conditions, and 0.75 (95% CI 0.69, 0.83) for those from the Western Cape compared to the Eastern Cape.

Table 3. Sample description, and univariate and multivariate analysis of impact of COVID-19 vaccination on COVID-19 hospital expenditures.

	Hospital Expenditure**			% Difference in Hospital expenditurevs Reference Group***		Risk Ratio of Zero Hospital Expenditure vs. Reference Group****	
	USD(m's)	USD/Event	USD/ Individual/ Annum***	Unadjusted	Adjusted	Unadjusted	Adjusted
**Vaccination Status***							
Not Vaccinated	17.45	1,749	18	Reference	Reference	Reference	Reference
Part Vaccinated	1.12	1,319	6	-24.83% (-33.67%, -14.81%)	-32.62% (-40.86%, -23.22%)	2.28 (2.11, 2.45)	4.38 (4.05, 4.72)
Fully vaccinated	0.75	943	2	-46.40% (-53.44%, -38.29%)	-53.77% (-59.85%, -46.78%)	5.13 (4.75, 5.55)	18.57 (17.14, 20.11)
**Gender**							
Female	9.74	1,585	12	Reference	Reference	Reference	Reference
Male	9.58	1,752	13	10.66% (0.61%, 21.72%)	9.62% (0.74%, 19.27%)	0.97 (0.93, 1.01)	0.86 (0.83, 0.90)
**Age**							
Less than 18	0.47	506	1	Reference	Reference	Reference	Reference
Between 18-25	4.58	16,917	40	60.42% (2.42%, 151.27%)	67.61% (14.78%, 144.73%)	0.73 (0.64, 0.84)	0.54 (0.47, 0.62)
Between 25-40	0.22	91	1	146.40% (100.00%, 203.57%)	141.88% (99.27%, 193.59%)	0.29 (0.27, 0.32)	0.23 (0.21, 0.25)
Between 40-65	2.97	547	7	308.38% (237.53%, 394.10%)	285.96% (222.53%, 361.86%)	0.12 (0.12, 0.13)	0.11 (0.10, 0.11)
Greater than 65	11.07	4,301	110	256.51% (191.53%, 335.98%)	259.50% (195.45%, 337.44%)	0.06 (0.06, 0.07)	0.06 (0.05, 0.06)
**Chronic Condition?**							
No	5.67	1,317	5	Reference	Reference	Reference	Reference
Yes	13.65	1,868	38	41.99% (28.83%, 56.48%)	19.43% (8.29%, 31.71%)	0.17 (0.16, 0.18)	0.25 (0.24, 0.27)
**Province**							
Eastern Cape	1.07	1,298	9	Reference	Reference	Reference	Reference
Free State	0.72	1,568	11	21.42% (-6.59%, 57.83%)	27.62% (-4.09%, 69.82%)	0.99 (0.87, 1.12)	0.85 (0.75, 0.96)
Gauteng	8.03	1,668	11	28.81% (9.12%, 52.04%)	33.79% (14.86%, 55.85%)	1.05 (0.97, 1.14)	0.77 (0.70, 0.83)
KwaZulu-Natal	3.90	1,869	16	44.39% (20.23%, 73.40%)	49.97% (26.55%, 77.72%)	0.83 (0.76, 0.91)	0.75 (0.68, 0.82)
Limpopo	0.25	914	5	-29.88% (-51.05%, 0.45%)	-23.45% (-43.88%, 4.43%)	1.45 (1.25, 1.68)	0.99 (0.85, 1.15)
Mpumalanga	0.41	1,277	8	-1.71% (-28.12%, 34.42%)	2.42% (-22.98%, 36.19%)	1.17 (1.02, 1.35)	0.99 (0.85, 1.14)
North West	0.60	1,435	11	10.62% (-11.74%, 38.65%)	4.39% (-14.90%, 28.04%)	0.92 (0.81, 1.05)	0.77 (0.67, 0.88)
Northern Cape	0.43	1,774	15	36.96% (-5.53%, 98.55%)	45.85% (-5.31%, 124.65%)	0.83 (0.71, 0.98)	0.72 (0.61, 0.85)
Western Cape	3.91	1,798	16	38.87% (16.13%, 66.05%)	39.10% (18.19%, 63.72%)	0.79 (0.72, 0.86)	0.75 (0.69, 0.83)
**Total**	19.32	1,664	12				

Vaccination Status*: Status as time of hospital event if hospitilised, and as end of study period if never hospitilised during study period

Hospital Expenditure**: South African Rands converted to US Dollary terms and discounted to 2022 value

USD/Individual/per annum***: Total USD/ number of individuals/study period (2.83 years)

% Difference in hospital expenditure***: Exponent of estimated coefficents (beta1) from count component output from negative binonmial model - 1 X 100: (exp(beta1)-1)x100.

Risk Ratio for zero hospital expenditure****: Exponent of estimated coefficents (beta1) from zero inflated component output from negative binonmial model : exp(beta1)

Discussion

With the emergence of different variants, differences in variant severity, and with reducing population susceptibility over time either due to immunity from previous infections or through vaccination, there were many moving parts during the full period of the COVID-19 pandemic. In contrast to many studies that have reported on the efficacy of the vaccine in clinical trial situations, in population sub-sets using test-negative controls[43], on specific viruses and/or vaccines, this study examined and provides insights on the impact of the COVID-19 vaccination on an aggregated basis in a "real world" population cohort of 550,332 insured individuals tracked over the entire 34 month period of the pandemic in South Africa, from 1 March 2020 to 31 December 2022. By the end of the study period, 12.2% of study individuals were partially vaccinated and 25.7% were fully vaccinated. The overall COVID-19 related hospitalisation rate for the period was 7.45.

The findings of this study indicated that COVID-19 vaccination substantially reduced the risk of COVID-19 related hospitalisation, hospital utilisation and hospital expenditures. The hospitalisation rate dropped from 10.29 for the non-vaccinated, to 4.47 for the partially vaccinated, and to 1.97 for the fully vaccinated. After adjustment for other risk factors, when compared to the non-vaccinated, the risk of hospitalisation was 94.51% lower for the partially vaccinated and 93.49% lower for the fully vaccinated. The hospital days per 1000 individuals per year dropped from 93.82 for non-vaccinated individuals to 44.11 for the partly vaccinated and to 20.52 for the fully vaccinated. After adjustment for other risk factors and when compared to the non-vaccinated, hospital utilisation was 17.70% lower for the partly vaccinated and 20.04% lower for the fully vaccinated. Compared to the non-vaccinated, the relative risk of zero hospital days was 4.34 for the partly vaccinated and 18.55 for the fully vaccinated. The hospital expenditure per individual per annum dropped from USD18 for non-vaccinated individuals to USD6 for the partly vaccinated and to USD2 for the fully vaccinated. After adjustment for other risk factors and when compared to the non-vaccinated, hospital expenditure was 32.83% lower for the partly vaccinated and 55.29% lower the fully vaccinated. Compared to the non-vaccinated, the relative risk of zero hospital expenditure was 4.38 for the partly vaccinated and 18.61 for the fully vaccinated.

The study findings on the impact of vaccination on the risk of hospitalisation are in line with the findings of other studies. An observational cohort study of >2 million adults in the Western Cape, South Africa over the period from 2020 to 2022 reported vaccine effectiveness of 45-92% for hospital admission with severe disease or death, and 25-90% for any admission or death[44]. Another South African study examining the impact of the J&J vaccine in health workers reported vaccine effectiveness of   75% (95% CI: 69%-82%) in preventing COVID-19-related hospital admissions requiring critical or intensive care, and 67% (95% CI: 62%-71%) in preventing COVID-19-related hospitalisations[17]. More broadly, at least 3 meta- analyses have reported similar results. The first, incorporating 51 studies to assess vaccine effectiveness in the “real-world” reported the COVID-19 vaccine to be 97.2% (95% CI 96.1-98.3%)

effective against hospitalisation, and 97.4% (95% CI 96.0-98.8%) effective against admission to the intensive care unit.[10] The second, a review of 58 studies, reported a single dose of vaccine to be 66% (50–81%) effective in preventing hospitalization, and 45% (42–49%) in preventing Intensive Care Unit (ICU) admissions. The review reported that two doses of the vaccine were 93% (89–96%) effective in preventing hospitalization and 96% (93–98%) effective in preventing ICU admissions[11]. The third, a systematic review of 42 studies examining the efficacy of a range of COVID-19 vaccines in a variety of populations, concluded that the Pfizer/BioNTech vaccine had >90% effectiveness against infections requiring hospitalization, after the second dose. A single dose of the J&J vaccine was >60% effective against infection requiring hospitalization[45].

The study findings in terms of the impact of COVID-19 vaccination on hospital utilisation and expenditure are also in alignment with findings of other studies. A retrospective study based on electronic health records of more than 180 for-profit hospitals across 20 states in the United States and comprising 29,732 (21,525 unvaccinated and 8,207 vaccinated) patients admitted with COVID-19 from January to December 2021 reported vaccinated patients having a 2.13 (CI 2.73–1.55 days) lower average length of stay (LoS) when compared with non-vaccinated[46]. A retrospective cohort study of 1927 hospitalized patients infected with COVID-19 in a tertiary-level public hospital in the Community of Madrid (Spain) between 1 January and 31 July 2021 found a reduction in hospital stay in vaccinated patients of 3.17 days (CI 95%: 5.88 to 0.47)[47]. A study on COVID-19 positive patients (716 fully vaccinated and 2487 unvaccinated) hospitalized in Norway between 1 February and 30 November 2021, found that, after adjustment, the length of stay of fully vaccinated patients was 38% (95% CI: 19%–52%) shorter than the non-vaccinated[48]. A retrospective cohort study of patients with COVID-19 admitted to a hospital in China (222 vaccinated, 327 unvaccinated) between January 2023 and February 2023, reported an after-adjustment reduction in the LoS of 1.2 days (14% 95 %CI -24%, 4%) [49]. In terms of evidence from LMICs, a retrospective observational study carried out in a tertiary care hospital in

western India, from April 1 to June 30 2021, reported unadjusted ward LoS of 6.21 ± 3.20 for vaccinated patients and 5.56 ± 4.55 days ($p < 0.001$) for non-vaccinated patients. The unadjusted mean length of ICU stay for vaccinated patients is reported to be 4.47 ± 2.3 days for vaccinated patients versus 6.29 ± 2.19 days ($p < 0.001$) for non-vaccinated patients [50]. A retrospective cohort study of 1407 patients (812 vaccinated, 1029 unvaccinated) admitted with COVID-19 at a hospital in Karachi, Pakistan reported an unadjusted median and (interquartile range) length of hospital stay of 7 (4–12) days for unvaccinated COVID-19 patients compared to 6 (4–11) days in the vaccinated group[51].

In the only study we able to find reporting on impact of vaccination on hospital expenditures, a retrospective cohort study carried on individuals hospitalized for COVID-19 between April 2021 to January 2022 at a large academic medical center in the United States of America, found that that the difference in the unadjusted cost of hospitalization for the vaccinated ($119,630) versus the unvaccinated ($191,146) was not statistically significant (P=0.06), after adjusting for age and comorbidities. Vaccinated patients experienced a 26% lower cost of hospitalization compared with unvaccinated patients (P = 0.004). Unvaccinated patients incurred a significantly higher cost of hospitalization per day ($29,425 vs $13,845 P < 0.0001)[52].

In contrast to many other COVID-19 vaccination impact studies that have reported on specific contexts, this study examined and provides insights on the aggregated impact of the COVID-19 vaccination in a “real world” population cohort over the period of the pandemic. The findings of the study unequivocally confirm that over the entire period - covering the emergence of the various strains of the virus, different vaccines and vaccine regimens and evolving clinical protocols - COVID-19 vaccination was very effective in reducing the risk of COVID-19 related hospitalisation, hospital utilisation and hospital expenditures. The research also contributes towards addressing some of the relative gaps in the evidence database – the impact of COVID-19 vaccination in South Africa and other LMICs, in insured populations, and the impact on hospital utilisation and expenditures. It also adds to the body of evidence based on use of

real-world data in measuring impacts. The information generated from the study can be used in other analyses to further assess the effectiveness, cost, and cost effectiveness of COVID-19 vaccination in preventing COVID-19 related morbidity during the pandemic. This study demonstrates the untapped potential for using Real World Data (RWD) in health policy decision making and planning[31].

From a broader policy perspective, the responses to the COVID-19 pandemic reflected and accentuated the underlying socio-ideological and political divides within and across societies. A key fault-line in these divides was differing views on individual versus societal freedoms and responsibilities, and the role of personal responsibility[53] (any health policy that prioritises in response to factors that are posited as being under individual control - by linking either the relative payment for treatment or the extent of treatment to these factors). The findings raise questions as to whether there was a case for applying personal responsibility for COVID-19 vaccination in the study population, as implemented in some countries during the COVID-19 pandemic[54, 55]. For example, the Singapore national health insurance system required those who remained "unvaccinated by choice" after 8 December 2021 to pay for their own COVID-19 treatment on the grounds that "unvaccinated persons make up a sizeable majority of those who require intensive inpatient care, and disproportionately contribute to the strain on our healthcare resources" [56]. The imposition of personal responsibility is however complex and contested, and it is critical that its application is fair, just and acceptable to the population as views on these issues play a major role in the social cohesiveness and functioning of societies[53, 57, 58]. While this study has confirmed that COVID-19 vaccination had a major impact on hospital admissions, utilisation and expenditure, the case for applying personal responsibility would need to be assessed more broadly, including an assessment of the fairness and practicality of the intervention. Further research is required to explore these issues.

This analysis has several limitations. Only services and vaccines for which claims were submitted were analysed. This could have resulted in under-recording of COVID-19 cases and COVID-19 vaccinations

which could have resulted in an overstatement of the reported outcome. However, while such overstatement may reduce the impact of the vaccine, it is unlikely that it would have negated the impact entirely, given the magnitude of the impact. The analysis was undertaken at a macro aggregated level with a view to providing broader policy focused insights. This aggregation comes at the expense of providing a more granular clinically focused view on the specific components of the pandemic (e.g. different variants of the virus, the different vaccines etc.) as has been done in most other studies. Evidence now indicates that although rare, there are safety and side-effects concerns regarding the COVID-19 vaccine[59, 60]. These were not taken into consideration in this study. The study was carried out on a cohort of individuals with private health insurance. While the results of the study are arguably generalizable to the insured population, generalizability to the broader South African populations is limited given the differences in demographics, socio-economic profiles, and access to care between the insured and non-insured populations. There are also differences in the clinical guidelines governing the public and private sectors in South Africa.

Conclusion

The findings of this study, based on a large cohort of insured individuals followed over the entire period of the pandemic using real world data, confirms that - all other things being equal - COVID-19 vaccination substantially reduced the risk of hospitalisation, hospital utilisation and expenditures. From a broader policy perspective, the findings raise questions as to whether there was a case for applying personal responsibility[53] for COVID-19 vaccination in the study population, an area for which further research is required. The information generated from the study could be used in other assessments of COVID-19 vaccination strategies aimed at better resource allocation, planning and prioritization strategies in the future as health care systems struggle to meet the immediate and longer-term increased health care demands in ever-more resource constrained environments.

References

1. Statement on the second meeting of the International Health Regulations (2005) Emergency Committee regarding the outbreak of novel coronavirus (2019-nCoV) [Internet]. Geneva WHO; 2020
2. WHO Director-General's opening remarks at the media briefing on COVID-19 – 11 March 2020 [Internet]. Geneva WHO; 2020
3. Thanh Le T, Andreadakis Z, Kumar A, Gomez Roman R, Tollefsen S, Saville M, et al. The COVID-19 vaccine development landscape. Nat Rev Drug Discov. 2020;19(5):305-6. doi: 10.1038/d41573-020-00073-5. PubMed PMID: 32273591.
4. Dzinamarira T, Nachipo B, Phiri B, Musuka G. COVID-19 Vaccine Roll-Out in South Africa and Zimbabwe: Urgent Need to Address Community Preparedness, Fears and Hesitancy. Vaccines. 2021;9(3). Epub 2021/04/04. doi: 10.3390/vaccines9030250. PubMed PMID: 33809002; PubMed Central PMCID: PMCPMC8000117.
5. World Health Organisation. WHO Coronavirus (COVID-19) Dashboard. https://covid19.who.int/table.
6. Bradshaw D, Dorrington R, Laubscher R, Groenewald P, Moultrie T. COVID-19 and all-cause mortality in South Africa – the hidden deaths in the first four waves. South African Journal of Science. 2022;118(5/6). doi: 10.17159/sajs.2022/13300.
7. Fathi M, Vakili K, Sayehmiri F, Mohamadkhani A, Hajiesmaeili M, Rezaei tavirani M, et al. The prognostic value of comorbidity for the severity of COVID-19: A systematic review and meta-analysis study. PLOS ONE. 2021;16:e0246190. doi: 10.1371/journal.pone.0246190.
8. Rees EM, Nightingale ES, Jafari Y, Waterlow NR, Clifford S, CA BP, et al. COVID-19 length of hospital stay: a systematic review and data synthesis. BMC Med. 2020;18(1):270. doi: 10.1186/s12916-020-01726-3. PubMed PMID: 32878619; PubMed Central PMCID: PMCPMC7467845.
9. Dessie ZG, Zewotir T. Mortality-related risk factors of COVID-19: a systematic review and meta-analysis of 42 studies and 423,117 patients. BMC Infectious Diseases. 2021;21(1):855. doi: 10.1186/s12879-021-06536-3.
10. Zheng C, Shao W, Chen X, Zhang B, Wang G, Zhang W. Real-world effectiveness of COVID-19 vaccines: a literature review and meta-analysis. International journal of infectious diseases : IJID : official publication of the International Society for Infectious Diseases. 2022;114:252-60. Epub 2021/11/21. doi: 10.1016/j.ijid.2021.11.009. PubMed PMID: 34800687; PubMed Central PMCID: PMCPMC8595975.
11. Liu Q, Qin C, Liu M, Liu J. Effectiveness and safety of SARS-CoV-2 vaccine in real-world studies: a systematic review and meta-analysis. Infectious diseases of poverty. 2021;10(1):132. Epub 2021/11/16. doi: 10.1186/s40249-021-00915-3. PubMed PMID: 34776011; PubMed Central PMCID: PMCPMC8590867.
12. Xu K, Wang Z, Qin M, Gao Y, Luo N, Xie W, et al. A systematic review and meta-analysis of the effectiveness and safety of COVID-19 vaccination in older adults. Frontiers in immunology. 2023;14:1113156. Epub 2023/03/21. doi: 10.3389/fimmu.2023.1113156. PubMed PMID: 36936964; PubMed Central PMCID: PMCPMC10020204.
13. Gao P, Liu J, Liu M. Effect of COVID-19 vaccines on reducing the risk of long COVID in the real world: a systematic review and meta-analysis. International journal of environmental research and public health. 2022;19(19):12422.
14. Ssentongo P, Ssentongo AE, Voleti N, Groff D, Sun A, Ba DM, et al. SARS-CoV-2 vaccine effectiveness against infection, symptomatic and severe COVID-19: a systematic review and meta-

analysis. BMC Infect Dis. 2022;22(1):439. Epub 2022/05/08. doi: 10.1186/s12879-022-07418-y. PubMed PMID: 35525973; PubMed Central PMCID: PMCPMC9077344.
15. Collie S, Nayager J, Bamford L, Bekker LG, Zylstra M, Gray G. Effectiveness and Durability of the BNT162b2 Vaccine against Omicron Sublineages in South Africa. The New England journal of medicine. 2022;387(14):1332-3. Epub 2022/09/15. doi: 10.1056/NEJMc2210093. PubMed PMID: 36103455; PubMed Central PMCID: PMCPMC9511610.
16. Collie S, Champion J, Moultrie H, Bekker LG, Gray G. Effectiveness of BNT162b2 Vaccine against Omicron Variant in South Africa. The New England journal of medicine. 2022;386(5):494-6. Epub 2021/12/30. doi: 10.1056/NEJMc2119270. PubMed PMID: 34965358; PubMed Central PMCID: PMCPMC8757569.
17. Bekker LG, Garrett N, Goga A, Fairall L, Reddy T, Yende-Zuma N, et al. Effectiveness of the Ad26.COV2.S vaccine in health-care workers in South Africa (the Sisonke study): results from a single-arm, open-label, phase 3B, implementation study. Lancet. 2022;399(10330):1141-53. Epub 2022/03/21. doi: 10.1016/s0140-6736(22)00007-1. PubMed PMID: 35305740; PubMed Central PMCID: PMCPMC8930006 ViiV Health Care, and Gilead. .
18. Gray G, Collie S, Goga A, Garrett N, Champion J, Seocharan I, et al. Effectiveness of Ad26.COV2.S and BNT162b2 Vaccines against Omicron Variant in South Africa. New England Journal of Medicine. 2022;386(23):2243-5. doi: 10.1056/NEJMc2202061. PubMed PMID: 35507482.
19. Liu Y, Procter SR, Pearson CAB, Montero AM, Torres-Rueda S, Asfaw E, et al. Assessing the impacts of COVID-19 vaccination programme's timing and speed on health benefits, cost-effectiveness, and relative affordability in 27 African countries. BMC Medicine. 2023;21(1):85. doi: 10.1186/s12916-023-02784-z.
20. Pearson CAB, Bozzani F, Procter SR, Davies NG, Huda M, Jensen HT, et al. COVID-19 vaccination in Sindh Province, Pakistan: A modelling study of health impact and cost-effectiveness. PLoS Med. 2021;18(10):e1003815. Epub 2021/10/05. doi: 10.1371/journal.pmed.1003815. PubMed PMID: 34606520; PubMed Central PMCID: PMCPMC8523052.
21. Mukandavire Z, Nyabadza F, Malunguza NJ, Cuadros DF, Shiri T, Musuka G. Quantifying early COVID-19 outbreak transmission in South Africa and exploring vaccine efficacy scenarios. PLOS ONE. 2020;15(7):e0236003. doi: 10.1371/journal.pone.0236003.
22. Ali HA, Hartner A-M, Echeverria-Londono S, Roth J, Li X, Abbas K, et al. Vaccine equity in low and middle income countries: a systematic review and meta-analysis. Int J Equity Health. 2022;21(1):82. doi: 10.1186/s12939-022-01678-5.
23. Gozzi N, Chinazzi M, Dean NE, Longini Jr IM, Halloran ME, Perra N, et al. Estimating the impact of COVID-19 vaccine inequities: a modeling study. Nature communications. 2023;14(1):3272. doi: 10.1038/s41467-023-39098-w.
24. Duroseau B, Kipshidze N, Limaye RJ. The impact of delayed access to COVID-19 vaccines in low- and lower-middle-income countries. Frontiers in public health. 2023;10. doi: 10.3389/fpubh.2022.1087138.
25. Kassanjee R, Davies M-A, Heekes A, Mahomed H, Hawkridge AJ, Morden E, et al. COVID-19 Vaccine Uptake and Effectiveness by Time since Vaccination in the Western Cape Province, South Africa: An Observational Cohort Study during 2020–2022. Vaccines. 2024;12(6):628. PubMed PMID: doi:10.3390/vaccines12060628.
26. World Health Organisation. Private Sector Landscape in Mixed Health Systems. Geneva: WHO, 2020.
27. Statistics South Africa. General Household Survey, 2016. Pretoria: STATS SA, 2016.
28. Ncube NB, Solanki GC, Kredo T, Lalloo R. Antibiotic prescription patterns of South African general medical practitioners for treatment of acute bronchitis. SAMJ. 2017;107(2):119-22. Epub 2017/02/22. doi: 10.7196/SAMJ.2017.v107i2.11276. PubMed PMID: 28220736.

29. Solanki G, Cornell M, Lalloo R. Uptake and cost of influenza vaccines in a private health insured South African population. S Afr J Infect Dis. 2018;33(5). doi: 10.4102/sajid.v33i5.138. PubMed PMID: 32051821; PubMed Central PMCID: PMCPMC7015168.
30. Solanki G, Fawcus S, Daviaud E. A cross sectional analytic study of modes of delivery and caesarean section rates in a private health insured South African population. PLOS ONE. 2019;14(6):e0219020. doi: 10.1371/journal.pone.0219020.
31. Solanki G, Wilkinson T, Bansal S, Shiba J, Manda S, Doherty T. COVID-19 hospitalization and mortality and hospitalization-related utilization and expenditure: Analysis of a South African private health insured population. PLOS ONE. 2022;17(5):e0268025. doi: 10.1371/journal.pone.0268025.
32. Jassat W, Mudara C, Ozougwu L, Tempia S, Blumberg L, Davies M-A, et al. Difference in mortality among individuals admitted to hospital with COVID-19 during the first and second waves in South Africa: a cohort study. The Lancet Global Health. 2021;9(9):e1216-e25. doi: 10.1016/S2214-109X(21)00289-8.
33. Council for Medical Schemes. Annual Report 2021/22. https://www.medicalschemes.co.za/cms-annual-report-2021-22/.
34. Centre for Respiratory Diseases and Meningitis. Proposed definition of COVID-19 wave in South Africa. Communicable Diseases Communiqué. November 2021, Vol. 20 (11). https://www.nicd.ac.za/wp-content/uploads/2021/11/Proposed-definition-of-COVID-19-wave-in-South-Africa.pdf.
35. National Department of Health.. Media Release: Cabinet approves changes to COVID-19 regulations. 30 December 2021. https://sacoronavirus.co.za/2021/12/30/media-release-cabinet-approves-changes-to-covid-19-regulations/.
36. Council for Medical Schemes. Prescribed Minimum Benefits. https://www.medicalschemes.co.za/resources/pmb/.
37. Council for Medical Schemes. Guidelines for the Identification of Beneficiaries with Risk Factors in Accordance with the Entry and Verification Criteria. Version 10.1. Pretoria: CMS.
38. Havers FP, Pham H, Taylor CA, Whitaker M, Patel K, Anglin O, et al. COVID-19-Associated Hospitalizations Among Vaccinated and Unvaccinated Adults 18 Years or Older in 13 US States, January 2021 to April 2022. JAMA Internal Medicine. 2022;182(10):1071-81. doi: 10.1001/jamainternmed.2022.4299.
39. Discovery Health. When are you fully vaccinated? September 2021. https://www.discovery.co.za/corporate/covid19-when-are-you-fully-vaccinated.
40. South African Medical Association. How to Code for COVID-19 and the necessity of legal disclaimers (Updated Article). Med-e-Mail. https://www.samedical.org/cmsuploader/viewArticle/1119.
41. Stats SA. PO141- Consumer Price Index (CPI). CPI History. 11 June 2024. https://www.statssa.gov.za/publications/P0141/CPIHistory.pdf?SCH=73795. .
42. South Africa. Protection of Personal Information Act No. 4 of 2013. https://www.gov.za/documents/promotion-access-information-act#:~:text=The%20Promotion%20of%20Access%20to,provide%20for%20matters%20connected%20theretith. (accessed 1 August 2022).
43. Chua H, Feng S, Lewnard JA, Sullivan SG, Blyth CC, Lipsitch M, et al. The Use of Test-negative Controls to Monitor Vaccine Effectiveness: A Systematic Review of Methodology. Epidemiology (Cambridge, Mass). 2020;31(1):43-64. Epub 2019/10/15. doi: 10.1097/ede.0000000000001116. PubMed PMID: 31609860; PubMed Central PMCID: PMCPMC6888869.
44. Kassanjee R, Davies MA, Heekes A, Mahomed H, Hawkridge AJ, Wolmarans M, et al. COVID-19 vaccine uptake and effectiveness by time since vaccination in the Western Cape province, South Africa: An observational cohort study during 2020-2022. medRxiv. 2024. Epub 2024/02/12. doi:

10.1101/2024.01.24.24301721. PubMed PMID: 38343866; PubMed Central PMCID: PMCPMC10854330.
45. Mohammed I, Nauman A, Paul P, Ganesan S, Chen K-H, Jalil SMS, et al. The efficacy and effectiveness of the COVID-19 vaccines in reducing infection, severity, hospitalization, and mortality: a systematic review. Human vaccines & immunotherapeutics. 2022;18(1):2027160.
46. Lee SW, Ma D, Davoodian A, Ayutyanont N, Werner B. COVID-19 vaccination decreased COVID-19 hospital length of stay, in-hospital death, and increased home discharge. Preventive medicine reports. 2023;32:102152. Epub 2023/02/23. doi: 10.1016/j.pmedr.2023.102152. PubMed PMID: 36811076; PubMed Central PMCID: PMCPMC9933576.
47. Moreno-Nunez P, Bueno-Cavanillas A, San Jose-Saras D, Vicente-Guijarro J, Fernández Chávez AC, Aranaz-Andrés JM, et al. How Does Vaccination against SARS-CoV-2 Affect Hospitalized Patients with COVID-19? Journal of clinical medicine. 2022;11(13). Epub 2022/07/10. doi: 10.3390/jcm11133905. PubMed PMID: 35807189; PubMed Central PMCID: PMCPMC9267443.
48. Whittaker R, Bråthen Kristofferson A, Valcarcel Salamanca B, Seppälä E, Golestani K, Kvåle R, et al. Length of hospital stay and risk of intensive care admission and in-hospital death among COVID-19 patients in Norway: a register-based cohort study comparing patients fully vaccinated with an mRNA vaccine to unvaccinated patients. Clinical microbiology and infection : the official publication of the European Society of Clinical Microbiology and Infectious Diseases. 2022;28(6):871-8. Epub 2022/02/28. doi: 10.1016/j.cmi.2022.01.033. PubMed PMID: 35219807; PubMed Central PMCID: PMCPMC8872711.
49. Fang X, Tao G, Zhou H, Zhou Y. Vaccines reduced hospital length of stay and fraction of inspired oxygen of COVID-19 patients: A retrospective cohort study. Preventive medicine reports. 2024;39:102632. Epub 2024/02/13. doi: 10.1016/j.pmedr.2024.102632. PubMed PMID: 38348219; PubMed Central PMCID: PMCPMC10859302.
50. Desai A, Desai P, Mehta J, Sachora W, Bharti N, Patel T, et al. Measuring the impact of a single dose of ChAdOx1 nCoV-19 (recombinant) coronavirus vaccine on hospital stay, ICU requirement, and mortality outcome in a tertiary care centre. International journal of infectious diseases : IJID : official publication of the International Society for Infectious Diseases. 2021;113:282-7. Epub 2021/10/25. doi: 10.1016/j.ijid.2021.10.032. PubMed PMID: 34688949; PubMed Central PMCID: PMCPMC8529545.
51. Sayeed MA, Shalim E, Farooqui F, Farman S, Khan M, Iqbal A, et al. Comparison of the Disease Severity and Outcome of Vaccinated COVID-19 Patients with Unvaccinated Patients in a Specialized COVID-19 Facility: A Retrospective Cohort Study from Karachi, Pakistan. Vaccines. 2023;11(7). Epub 2023/07/29. doi: 10.3390/vaccines11071178. PubMed PMID: 37514994; PubMed Central PMCID: PMCPMC10386639.
52. Somani ST, Firestone RL, Donnelley MA, Sanchez L, Hatfield C, Fine J, et al. Impact of Vaccination on Cost and Course of Hospitalization Associated with COVID-19 Infection. Antimicrobial stewardship & healthcare epidemiology : ASHE. 2023;3(1):e19. Epub 2023/01/31. doi: 10.1017/ash.2022.364. PubMed PMID: 36714292; PubMed Central PMCID: PMCPMC9879923.
53. Feiring E. Lifestyle, responsibility and justice. Journal of medical ethics. 2008;34(1):33-6. doi: 10.1136/jme.2006.019067. PubMed PMID: 18156519.
54. Yang YT, Chen B. Imposing a health insurance surcharge on the unvaccinated. The American journal of managed care. 2022;28(7):310-2. Epub 2022/07/20. doi: 10.37765/ajmc.2022.89169. PubMed PMID: 35852879.
55. Lydia Wheeler. Bloomberg Law. Unvaccinated to Pay More Out-of-Pocket Under New Covid Policies. 2 February 2022. https://news.bloomberglaw.com/health-law-and-business/unvaccinated-to-pay-more-out-of-pocket-under-new-covid-policies.

56. Ministry of Health Singapore. Calibrated adjustments in stabilisation phase. 8 November 2021. https://www.moh.gov.sg/news-highlights/details/calibrated-adjustments-in-stabilisation-phase_8Nov2021.
57. Cappelen AW, Norheim OF. Responsibility, fairness and rationing in health care. Health Policy. 2006;76(3):312-9. doi: 10.1016/j.healthpol.2005.06.013. PubMed PMID: 16112248.
58. Sharkey K, Gillam L. Should patients with self-inflicted illness receive lower priority in access to healthcare resources? Mapping out the debate. Journal of medical ethics. 2010;36(11):661-5. doi: 10.1136/jme.2009.032102.
59. Chirico F, Teixeira da Silva JA, Tsigaris P, Sharun K. Safety & effectiveness of COVID-19 vaccines: A narrative review. The Indian journal of medical research. 2022;155(1):91-104. Epub 2022/07/22. doi: 10.4103/ijmr.IJMR_474_21. PubMed PMID: 35859436; PubMed Central PMCID: PMCPMC9552389.
60. Beatty AL, Peyser ND, Butcher XE, Cocohoba JM, Lin F, Olgin JE, et al. Analysis of COVID-19 Vaccine Type and Adverse Effects Following Vaccination. JAMA network open. 2021;4(12):e2140364. Epub 2021/12/23. doi: 10.1001/jamanetworkopen.2021.40364. PubMed PMID: 34935921; PubMed Central PMCID: PMCPMC8696570

Supplementary Table.

Table S1. Comparison of study population versus rest of the insured in South Africa

	Study Population	Rest of the insured population **	Total Insured Population
Number of Families	196,967	3,850,675	4,047,642
Number of lives covered	448,716	8,472,995	8,921,711
Average age per life covered	30.7	33.9	33.71
Pensioner ratio (65+ years)	6.7%	9.2%	9.1%
No. of dependents per family	2.3	1.1	1.2
Average contributions (in SA Ran	20,664	23,064	22,943
Average Health care expenditure	18,320	21,000	20,865

* Per life per annum

** Excluding Study Population

Click here to access/download;Figure;Figure 1 approach to extraction pdf.pdf

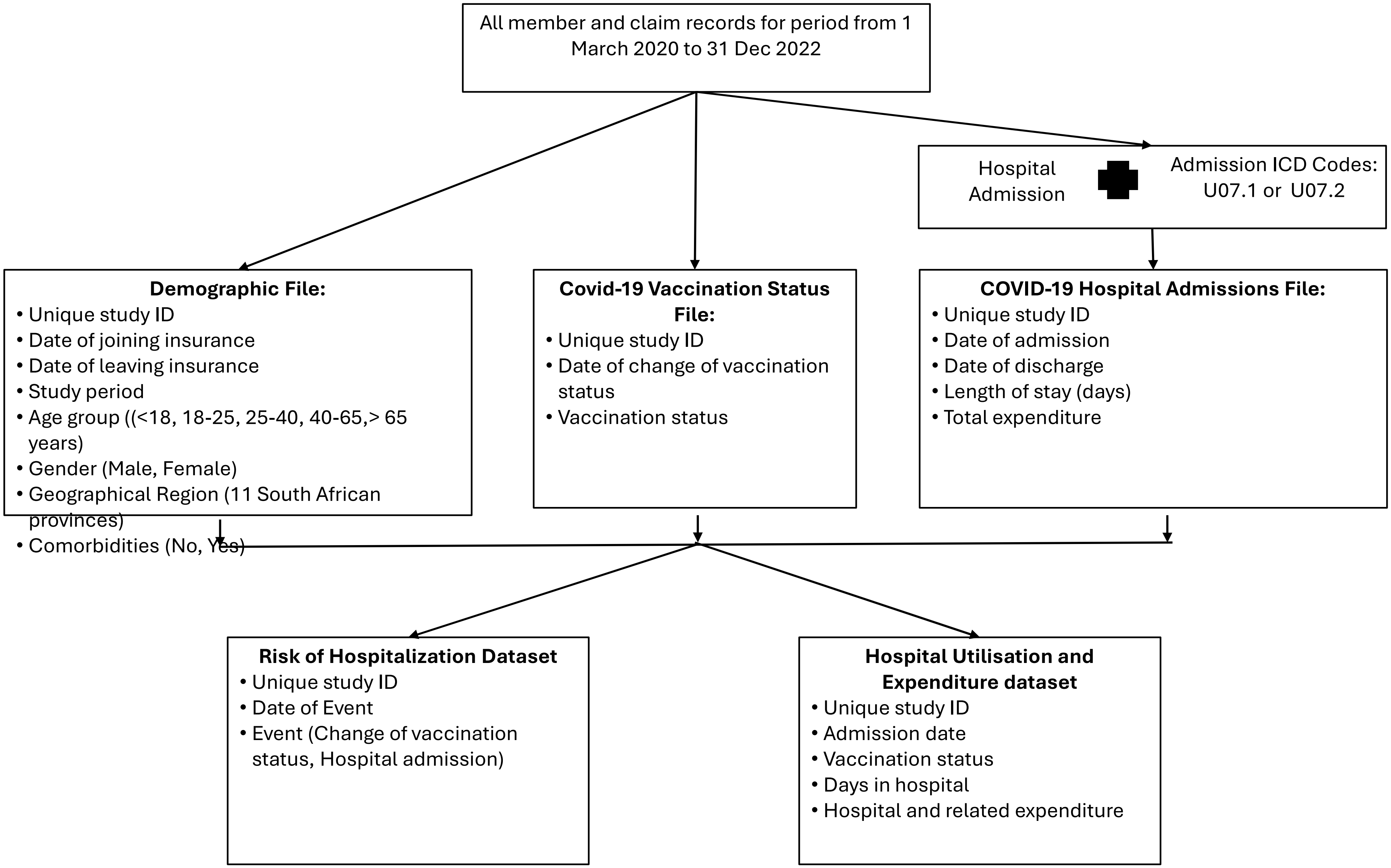

Figure

Click here to access/download;Figure;Figure 1 approach to extraction Powerpoint.pptx

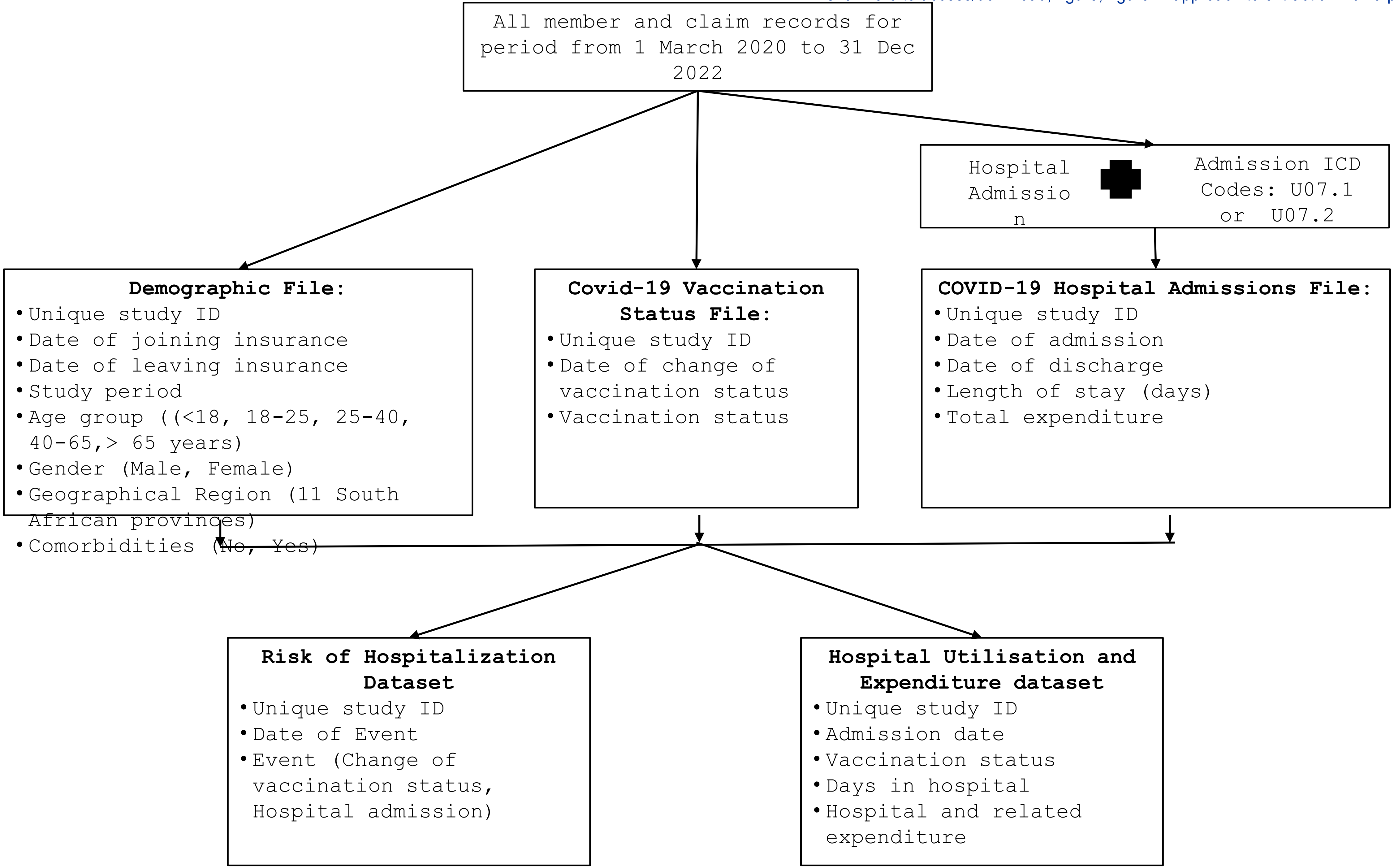

tables in excel

Click here to access/download
Supporting Information
tables Impact of COVID vac excel.xlsx

Click here to access/download
Supporting Information
Ethics Approval G Solanki.pdf
