# Supplementary material for: Impact of COVID-19 vaccination on hospitalization, hospital utilization and expenditure for COVID-19: A retrospective cohort analysis of a South African private health insured population
Source: PLoS One. 2025 Jan 24;20(1):e0317686. doi: 10.1371/journal.pone.0317686 (PMC11760578; doi:10.1371/journal.pone.0317686)
Supplement: Attachment — Submitted filename: PONE-D-24-26424_R2.pdf [file pone.0317686.s007.pdf]

PLOS ONE

Impact of COVID-19 vaccination on hospitalization, hospital utilization and expenditure for COVID-19: a retrospective cohort analysis of a South African private health insured population.

--Manuscript Draft--

**Manuscript Number:**	PONE-D-24-26424R2
**Article Type:**	Research Article
**Full Title:**	Impact of COVID-19 vaccination on hospitalization, hospital utilization and expenditure for COVID-19: a retrospective cohort analysis of a South African private health insured population.
**Short Title:**	Impact of COVID-19 vaccination hospitalisation for COVID-19 in a South African private insured population.
**Corresponding Author:**	Geetesh Solanki<br>SAMRC: South African Medical Research Council<br>Cape Town, Western Cape SOUTH AFRICA
**Keywords:**	COVID-19; COVID-19 vaccination; Hospitalization; Healthcare utilization; Healthcare expenditure; Retrospective cohort study; South Africa; Private health insurance; COVID-19 morbidity; Vaccine effectiveness; Pandemic response; Health economics; COVID-19 outcomes; Vaccination impact; Hospital cost analysis; Insurance claims data; low and middle-income countries; developing countries
**Abstract:**	This study quantifies the impact of COVID-19 vaccination on hospitalization for COVID-19 infection in a South African private health insurance population. This retrospective cohort study is based on the analysis of demographic and claims records for 550,332 individuals belonging to two health insurance funds between 1 March 2020 and 31 December 2022. A Cox Proportional Hazards model was used to estimate the impact of vaccination (non-vaccinated, partly vaccinated, fully vaccinated) on COVID-19 hospitalization risk; and zero-inflated negative binomial models were used to estimate the impact of vaccination on hospital utilization and hospital expenditure for COVID-19 infection, with adjustments for age, sex, comorbidities and province of residence. In comparison to the non-vaccinated, the hospitalization rate for COVID-19 was 94.51% (aHR 0.06, 95%CI 0.06, 0.07) and 93.49% (aHR 0.07, 95%CI 0.06, 0.07) lower for the partly and fully vaccinated respectively; hospital utilization was 17.70% (95% CI 24.78%, 9.95%) and 20.04% (95% CI 28.26%, 10.88%) lower; the relative risk of zero hospital days was 4.34 (95% CI 4.02, 4.68) and 18.55 (95% CI 17.12, 20.11) higher; hospital expenditure was 32.83% (95% CI 41.06%, 23.44%) and 55.29% (95% CI 61.13%, 48.57%) lower; and the relative risk of zero hospital expenditure was 4.38 (95% CI 4.06, 4.73) and 18.61 (95% CI 17.18, 20.16) higher for the partly and fully vaccinated respectively. Taken together, findings indicate that all measures of hospitalization for COVID-19 infection were significantly lower in the partly or fully vaccinated in comparison to the non-vaccinated. The use of real-world data and an aggregated level of analysis resulted in the study having several limitations. While the overall results may not be generalizable to other populations, the findings add to the evidence based on the impact of COVID-19 vaccination during the period of the pandemic.
**Order of Authors:**	Geetesh Solanki<br>Susan Cleary<br>Francesca Little
**Opposed Reviewers:**	
**Response to Reviewers:**	Mickael Essouma, M. D.<br>Academic Editor<br>PLOS ONE<br>Dear Mickael<br>Response to reviewer comments on Ref.: PONE-D-24-26424<br>Impact of COVID-19 vaccination on COVID-19 hospitalisation, hospital related

Powered by Editorial Manager® and ProduXion Manager® from Aries Systems Corporation

utilisation and expenditure: Analysis of a South African private health insured population.
Thank you for the reviewer comments on our manuscript. We have revised the manuscript and summarise our response to the comments below.

Additional Editor Comments:
The authors have substantially improved their articles. However, before it can be accepted for publication as it stands, they still need to make some amendments as described below.
ABSTRACT. The abstract contains 500 words. The abstract should be based on direct and to-the-point communication highlighting salient information from the full-text in a maximum of 300 words.
Response: We have reduced the abstract word count to 290 words.
INTRODUCTION. No comment.
METHODS. Did you use printed or electronic (REDCap for example) questionnaires/data extraction sheet (keywords of the "Data collection" sub-section) to record data from patients' insurance claim records? This information needs to be provided under the "Data collection" sub-section of the Methods section.
Response: We have added the following sentence under data collection to provide greater clarity on the data capture process and formats. "Electronic demographic and claims records for the insured individuals, stored on a Microsoft SQL Server in the NMG data warehouse, were extracted in comma-separated values (CSV) format and then imported into STATA/SE 16.1for data cleaning, descriptive analyses, and statistical modeling."

Because "exposure" and "outcome" are keywords in the methods section of a cohort study, it is important to specify the exposure in this study (COVID-19 vaccination) in the methods section. You could do this in the first sub-section "Study design, period and population". It is also important to have a sub-section (for example between the "Data collection" and "Statistical analysis" sub-sections) "Outcomes of interest" (in the Methods) where you should clearly state the outcomes in your study: hospitalisation, hospital utilisation, and spending for COVID-19 infection. So, the text on lines 178-181 of the current manuscript would better be edited as proposed here in the "Outcomes of interest" sub-section.
Response: We have added a "Exposure and outcomes of Interest" section and moved lines 178-186 (earlier draft) to the section. In the section, we have added wording to more clearly specify the exposures and outcomes of interest for the study
For clarity purposes, it would be better to specify "COVID-19 comorbidities" rather than just write "comorbidities" on lines 137, 139 and 140.
Response: We have specified COVID-19 comorbidities as recommended in the "Exposures and outcomes interest" section
This means the "Statistical analysis" sub-section of the Methods section should be further edited in a way that clearly highlights:
(i)whether you exported data from a software to the statistical analysis software used,
(ii) complete records of softwares used for statistical analysis,
Response: We have added wording to address this under the data collection section.
(iii)the statistical analyses carried out (assessment of the effect of COVID-19 vaccination on study outcomes), clear description of statistical tests used for that purpose, steps observed during those statistical analyses, and how did you consider test results to be statistically significant, and finally how you report results in the article (including effect magnitude estimates such as hazard ratios, rates [with 95% confidence intervals], figures, tables…). Along these lines, should the sentence "A Cox Proportional Hazards model was used to estimate the relative hazard of hospitalisation" not be edited to read "A cox proportional Hazards model was used to estimate the hazard for hospitalisation in vaccinated versus non-vaccinated subjects." (doi:10.1097/EDE.0b013e3181c1ea43.)?
Response: We have edited the statistical methods section to further clarify the methods used for analysis and reporting. We have added wording to clarify how effect sizes estimated from the models are reported in the paper.We have revised the "A Cox Proportional Hazard model…" sentence as suggested. On the matter of statistical significance, we have added wording to indicate that "No specific cut-off was used to determine statistical significance, and no p-values are reported. The focus is on effect sizes and their 95% confidence intervals."

	(iv)Did you make every effort to consider losses-to-follow up in statistical analyses?<br>Response: In Statistical analysis section we have added the following wording: “To factor in the censoring of the follow-period in the analysis, the Cox PH model was used for the primary analysis of the association between vaccination status and the risk of hospitalization. The model allows for those subjects who were never hospitalized during the follow-up period to be accommodated as censored observations.”<br><br>In the discussion section, we have added the following sentence: “Given that this is a retrospective observational cohort study, subject loss-to-follow up was not a concern. However, the observational nature of the study may have resulted in selection and confounding biases. Caution should therefore be exercised in interpreting the findings of this study.”<br>The text on lines 207 through 223 should be in a single paragraph because it is centred around a single idea. In other words, it is immediately after the sentence on lines 207-209 (that should be edited for simplicity and more clarity) that you should explain what the zero-inflated negative binomial model produces as you did on lines 214-223, but in a more concise way.<br>Response: We have removed the break to consolidate the wording into a paragraph. We have also added further details describing how the effect sizes for the association between predictor variables were determined from these models and reported in the manuscript.<br>These comments also make lead me to observe that the manuscript’s title (and therefore also keywords) needs to be edited in these likes for clarity purpose: “Impact of COVID-19 vaccination on hospitalisation, hospital utilisation and expenditure for COVID-19: a retrospective cohort analysis of a South African private health insured population”. Sentences addressing study outcomes should also be edited like this throughout the article.<br>Response: We have re-worded the manuscript title and text as suggested. We have also added to the keyword list.<br>Is it "Partly vaccinated" or "partially vaccinated" throughout the manuscript (including in Tables)?<br>Response: We reworded all instances of partially vaccinated to partly vaccinated to ensure consistency.<br>RESULTS.<br>Consider adding 95% confidence interval to rates reported in the results section (at least in the full-text manuscript).<br>Response: We have added the 95% confidence intervals for the unadjusted rates in the text. Adding them to the tables made the tables very cumbersome.<br>The text on lines 235-240 fails to mention the mean/median duration of follow-up of this retrospective cohort study.<br>Response: The cohort for the study was all individuals (550,332) who belonged to the two health insurance funds at any time between 1 March 2020 and 31 December 2022. We indicate in the methods that “Although the overall study period extended from 1 March 2020 to 31 December 2022 (34 months or 2.83 years), there are some individuals who joined the health insurance funds after 1 March 2020 and some who left before 31 December 2022. For individuals who were members of the insurance funds for the entire period, the study start date and end date were recorded as their start and end dates. For those who joined the insurance funds after 1 March 2020 or left before 31 December 2022 the actual entry and exit dates were recorded as the study start or end dates.”<br>We have amended the text and added the average duration of membership of these individuals over the study period as an indicator of the follow-up period.<br>In the discussion section, we also added text to recognise possible biases due to subjects not being part of the funds for the entire follow-up period as a limitation of the study.<br>What was the proportion of loss-to-follow up?<br>Response: Following on the above, we have indicated in the text the proportion of individuals who were members of the funds for the entire period of the study.<br>Can you complete the footnotes of Table 1 with some information on "chronic conditions" (which chronic conditions did you observe, at least the major ones? This is especially important because those chronic conditions are also predictors of COVID-19 related hospitalisations.)?<br>Response: We have listed the Covid-19 comorbidities considered in the analysis in the

	and outcomes of interest section. These included: cancers, chronic renal disease, congestive cardiac failure, chronic obstructive pulmonary disease, diabetes mellitus, HIV, hypercholesterolaemia, hypertension, hypothyroidism, ischaemic heart disease, pregnancy and tuberculosis. We did not re-list the conditions as footnotes under Table 1 as it made the table quite cumbersome.<br>It is interesting to report in the manuscript why you chose Eastern Cape as the province of reference for the statistical analysis of the association between province and COVID-19 related hospitalisation?<br>Response: The Eastern Cape was the default choice as the reference group on account of it being first in the alphabet thus making it easier to reference in the table. We have not added this in the text as it should not change the interpretation.<br>In the footnotes of Table 2, is it not “status at time of hospitalisation” rather than “Status at tim,e of hospital event”?<br>Response: We have corrected the typo.<br>Should the title of Table 2 not be something like “Results of univariate and multivariate analysis of predictive factors of hospitalisation for COVID-19”?<br>Response: We have reworded the titles of the tables to read:<br>Table 1. Characteristics of study population<br>Table 2. Univariate and multivariate analysis of impact of COVID-19 vaccination on risk of hospitalisation for COVID-19 infection<br>Table 3. Univariate and multivariate analysis of impact of COVID-19 vaccination on hospital utilisation for COVID-19 infection<br>Table 4. Univariate and multivariate analysis of impact of COVID-19 vaccination on hospital expenditure for COVID-19 infection.<br>Is this punctuation “?” necessary after the phrase “Chronic condition”. And once more, it would be interesting to know the chronic conditions that were associated with hospitalisation for COVID-19.<br>Response: We have removed the “?”. As indicated above, we have listed the chronic conditions that were considered for the analysis in the text in the section on “Exposures and outcomes of interest”.<br>Would it not be better to replace “chronic conditions” by “Comorbidities” in Table 2 and “COVID-19 comorbidities” in Table 1?<br>Response: We have reworded as suggested.<br>I think it would be more right to write “hospitalisation for COVID-19” rather than COVID-19 related hospitalisation in Table 2 and throughout the manuscript. Same remarks for Tables 3, 4, S2 and S3.<br>Response: We have reworded as suggested.<br>Should the title of Table 1 not be “Characteristics of the study population”?<br>Response: We reworded as suggested.<br>Is it not "versus the rest of the insured" (instead of "versus rest of the rest of the insured" in the title of Table S1?<br>Response: We have corrected the wording.<br>Should it not read “Hospital utilisation for COVID-19 infection” on line 263? Same remark for the sub-title on line 288. Similarly, should it not be “The overall rate of hospital utilisation for COVID-19 infection was…” on line 264? Same remark for the sentence on line 289.<br>Response: We have reworded as suggested.<br>It would be better to report the costs in US dollars like this: $ the amount and then US, e.g., $12 US<br>Response: We have changed the notation of cost in US dollars to $ and specified in the methods section that the “$” refers to US dollars.<br>DISCUSSION. Line 321: 7.45 per?<br>Response: We have reworded the sentence.<br>Caution should be exercised when choosing words and interpreting findings in the discussion. For example, did you really assess risk factors (doi:10.1097/EDE.0b013e3181c1ea43.)?<br>Response: We have edited and reworded to make the wording softer.<br>To avoid creating confusion in the discussion section, I would advise to start with a statement of main study findings (as you did) in a paragraph, discuss each of the assessed ouctomes of interest (hospitalisation due to COVID-19 infection, hospital utilization due to COVID-19 infection, and direct costs due to COVID-19 infection) in a separate paragraph (shorter than current paragraphs), and finally state study strengths, limitations and implications (for policies, clinical practice, and future research using the same insurance records and other records) in one to two paragraphs.

Response: We have created a separate paragraph for each of the outcomes of interest and edited to reduce script length and number of references.
When stating study limitations in the discussion section, it is very important to address the generalizability of your findings (this is a reason why you should provide us with 95% confidence intervals of the estimated rates; so, the text on lines 399-403 should be in the limitations statement.) and unavoidable study limitations related to the study design (cohort in this case; so what about selection bias, unmeasured confounding?
Response: We have included the 95% confidence intervals for the findings of the study in the discussion section, reduced the number of other studies referred to and ensured that the 95% confidence intervals reported by the included studies are provided. We have also amended the wording to indicate that the Cox Proportional Hazards model and hazards ratios were used and why. We have reworded the text to more clearly acknowledge the limitations of the study, including the potential for selection and confounding bias and limitations regarding generalisability.
Line 340: "in most other studies". Which ones (you mentioned in the introduction that there is a lack of studies similar to this one)?
Response: We have edited the sentence to remove the reference to other studies.
Same remark on line 344 ("other studies"). And indeed, you do not report any effect magnitude estimate (RR, HR or even OR) from any of the studies cited on lines 343-360.
Response: We have edited the sentence to remove the reference to other studies.
If there are no studies comparable to yours (in terms of methodology) with effect magnitude estimates but only incidence estimates for hospitalization due to COVID-19 after exposure to COVID-19 vaccination, then discuss incidence rates and call for further studies to further assess effect magnitude estimates of hospitalisation due to COVID-19 infection after exposure to COVID-19 vaccination.
Response: We have revised the text to indicate the magnitude of the effect (together with 95% CI) of the cited studies. We used standard methods to analyse data of this nature. It is standard to use Cox PH to estimate hazard ratios in the presence of censoring. It is also common to use zero-inflated models for outcomes that are a mixture of a zero and non-zero component.
Not even a small comment about patient survival when you used a Cox proportional Hazards model (why was it not even a an (secondary at least?) outcome of interest in the first place? I guess patients were hospitalised for severe/critical COVID-19 and may be hospital utilisation for COVID-19 infection included hospital utilisation for COVID-19 cases not hospitalised (those with mild to moderate COVID_19 infection) and COVID-19 cases (those with severe/critical COVID-19 infection). However, there is no such specification in any part of the article not even in the discussion when addressing limitations. If the information on COVID-19 severity is available, it would also be great to specify which COVID-19 infection severity classification scheme was used in your study. Same remark for expenditure for COVID-19. I guess you are addressing direct costs in this article.
Response: We have amended the text to acknowledge the failure to examine the severity of disease or assessments of mortality as a limitation of the study. We have added the following sentence as a limitation of the study: "In addition, the available demographic and claims data did not enable an analysis of severity of COVID-19 infection. Survival analysis or COVID-19 related mortality related assessments were also not possible due to limitations of the mortality data."
Can you detail what was included in that spending (at least in a table uploaded as a supplemental material)? These bits of information will increase the validity and strength of your findings and interpretations.
Response: We indicate in the paper that " The total hospital and related expenditures were from an insurer perspective and reflected the amounts paid by the health insurance funds to cover the claims submitted by health care providers for services rendered to the hospitalized individuals."
The discussion section always requires a great editing effort so as to be as exhaustive as possible using as few words as possible.
Response: We have edited the discussion to reduce the word and reference count, and to align with the suggested format.
CONCLUSION. It should be revised to provide a direct and to-the-point communication on the main results readers should keep in mind when reading your study, as well as the most important research perspective for future studies on this topic. I advise avoiding strong words such as "confirm" because this is not an adjudicating data synthesis.

	Response: We have revised the conclusion as suggested and softened the wording.<br><br>DECLARATIONS. I do not see them (e.g., Conflicts of interests, Author contributions, and Acknowledgments after the CONCLUSION section and before “REFERENCES”).<br>Response: The declarations were directly entered onto the Editorial manager. We have now also included those declarations in the draft as recommended.<br>REFERENCES. I am unable to open the link to reference 17. So, make sure all the links provided work well.<br>Response: We have reviewed and corrected the references.<br>Additional Request -sent through 19 November 2024<br>The Academic Editor supervising your manuscript's peer review reached out to let us know that they forgot to include some final comments in the decision letter you received on November 14th. They asked that we provide you with the missing comments directly so that you can reply to them in your Response to Reviewers document and incorporate them into your revised manuscript as needed.<br>The specific comments from your manuscript's Academic Editor are as follows:<br>"Can you add information about patients' racial/ethnic groups? Did you assess whether race/ethnicity also affected the outcomes of interest? If you do not have information about race/ethnicity, at least make a comment on these issues in the limitations statement of the Discussion section."<br>Response: Information on race/ethnicity of the individuals was not available and therefore could not be considered in the analysis. We have added a sentence indicating the limitations of the data.
**Additional Information:**	
**Question**	**Response**
**Financial Disclosure**<br><br>Enter a financial disclosure statement that describes the sources of funding for the work included in this submission. Review the submission guidelines for detailed requirements. View published research articles from *PLOS ONE* for specific examples.<br><br>This statement is required for submission and **will appear in the published article** if the submission is accepted. Please make sure it is accurate.<br><br>**Funded studies**<br>Enter a statement with the following details:<br>• Initials of the authors who received each award<br>• Grant numbers awarded to each author<br>• The full name of each funder<br>• URL of each funder website<br>• Did the sponsors or funders play any role in the study design, data collection and analysis, decision to publish, or preparation of the manuscript?	Yes

Did you receive funding for this work?	
Please add funding details.<br>as follow-up to "**Financial Disclosure**<br><br>Enter a financial disclosure statement that describes the sources of funding for the work included in this submission. Review the submission guidelines for detailed requirements. View published research articles from *PLOS ONE* for specific examples.<br><br>This statement is required for submission and **will appear in the published article** if the submission is accepted. Please make sure it is accurate.<br><br>**Funded studies**<br>Enter a statement with the following details:<br>• Initials of the authors who received each award<br>• Grant numbers awarded to each author<br>• The full name of each funder<br>• URL of each funder website<br>• Did the sponsors or funders play any role in the study design, data collection and analysis, decision to publish, or preparation of the manuscript?<br><br>Did you receive funding for this work?"	The South African Medical Research Council provided support in the form of salaries for authors Geetesh Solanki, and the University of Cape Town for Francesca Little and Susan Cleary. None of these institutions had any additional role in the study design, data collection and analysis, decision to publish, or preparation of the manuscript. The specific roles of these authors are articulated in the 'author contributions' section."<br><br>Geetesh Solanki is also employed on a contractual basis by NMG Consultants and Actuaries, an independent consulting firm providing consulting and actuarial services to South African private health insurance funds. NMG provided access to the data used for the study but did not have any other role or involvement in the study design, data collection and analysis, decision to publish, or preparation of the manuscript. Geetesh Solanki's commercial affiliation to NMG therefore played no role in influencing the study.
Please select the country of your main research funder (please select carefully as in some cases this is used in fee calculation).<br>as follow-up to "**Financial Disclosure**<br><br>Enter a financial disclosure statement that describes the sources of funding for the work included in this submission. Review the submission guidelines for detailed requirements. View published research articles from *PLOS ONE* for specific examples.<br><br>This statement is required for submission and **will appear in the published article** if the submission is accepted. Please make	SOUTH AFRICA - ZA

sure it is accurate.<br><br>**Funded studies**<br>Enter a statement with the following details:<br>• Initials of the authors who received each award<br>• Grant numbers awarded to each author<br>• The full name of each funder<br>• URL of each funder website<br>• Did the sponsors or funders play any role in the study design, data collection and analysis, decision to publish, or preparation of the manuscript?<br><br>Did you receive funding for this work?"	
**Competing Interests**<br><br>Use the instructions below to enter a competing interest statement for this submission. On behalf of all authors, disclose any competing interests that could be perceived to bias this work—acknowledging all financial support and any other relevant financial or non-financial competing interests.<br><br>This statement is required for submission and **will appear in the published article** if the submission is accepted. Please make sure it is accurate and that any funding sources listed in your Funding Information later in the submission form are also declared in your Financial Disclosure statement.<br><br>View published research articles from *PLOS ONE* for specific examples.	Geetesh Solanki is employed on a contractual basis by the South African Medical Research Council and NMG Consultants and Actuaries, an independent consulting firm providing consulting and actuarial services to South African private health insurance funds. NMG provided access to the data used for the study but did not have any other role or involvement in the study design, data collection and analysis, decision to publish, or preparation of the manuscript and Geetesh Solanki's commercial affiliation to NMG therefore played no role in influencing the study. Geetesh Solanki's association with NMG does not alter our adherence to PLOS One policies on sharing data and materials.

**NO authors have competing interests**<br><br>Enter: *The authors have declared that no competing interests exist.*<br><br>**Authors with competing interests**<br><br>Enter competing interest details beginning with this statement:<br><br>*I have read the journal's policy and the authors of this manuscript have the following competing interests: [insert competing interests here]*<br><br>* typeset	
**Ethics Statement**<br><br>Enter an ethics statement for this submission. This statement is required if the study involved:<br><br>• Human participants<br>• Human specimens or tissue<br>• Vertebrate animals or cephalopods<br>• Vertebrate embryos or tissues<br>• Field research<br><br>Write "N/A" if the submission does not require an ethics statement.<br><br>General guidance is provided below. Consult the submission guidelines for detailed instructions. **Make sure that all information entered here is included in the Methods section of the manuscript.**	Ethics approval for the use of the secondary database used to carry out this study was granted by the Faculty of Health Sciences Human Research Ethics Committee of the University of Cape Town – approval number HREC Ref: 606/2023. No individual consent was required as the research was based on anonymised secondary claims data.<br>Data for the study were made available as part of NMG Consultants and Actuaries (NMG) commitment to support research initiatives with broader public health significance. The data were accessed in terms of and under the conditions set out in the agreement between NMG and the client schemes and between NMG and the researcher. The terms set out in these agreements ensured that the access to and use of the data is in full compliance with the requirements of the Protection of Personal Information (PoPI) Act in South Africa[42]. All the data were provided in a de-identified format and aggregated at an individual level and the research team had no access to information that would enable the identification of any individual. All findings are presented at an aggregate level and no confidential member, health care provider or scheme information is disclosed. Individual consent for the data was therefore not required.

**Format for specific study types**<br><br>**Human Subject Research (involving human participants and/or tissue)**<br>• Give the name of the institutional review board or ethics committee that approved the study<br>• Include the approval number and/or a statement indicating approval of this research<br>• Indicate the form of consent obtained (written/oral) or the reason that consent was not obtained (e.g. the data were analyzed anonymously)<br><br>**Animal Research (involving vertebrate animals, embryos or tissues)**<br>• Provide the name of the Institutional Animal Care and Use Committee (IACUC) or other relevant ethics board that reviewed the study protocol, and indicate whether they approved this research or granted a formal waiver of ethical approval<br>• Include an approval number if one was obtained<br>• If the study involved *non-human primates*, add *additional details* about animal welfare and steps taken to ameliorate suffering<br>• If anesthesia, euthanasia, or any kind of animal sacrifice is part of the study, include briefly which substances and/or methods were applied<br><br>**Field Research**<br><br>Include the following details if this study involves the collection of plant, animal, or other materials from a natural setting:<br>• Field permit number<br>• Name of the institution or relevant body that granted permission	
**Data Availability**<br><br>Authors are required to make all data underlying the findings described fully available, without restriction, and from the time of publication. PLOS allows rare exceptions to address legal and ethical concerns. See the PLOS Data Policy and FAQ for detailed information.	Yes - all data are fully available without restriction

A Data Availability Statement describing where the data can be found is required at submission. Your answers to this question constitute the Data Availability Statement and **will be published in the article**, if accepted.<br><br>**Important:** Stating 'data available on request from the author' is not sufficient. If your data are only available upon request, select 'No' for the first question and explain your exceptional situation in the text box.<br><br>Do the authors confirm that all data underlying the findings described in their manuscript are fully available without restriction?	
**Describe where the data may be found in full sentences. If you are copying our sample text, replace any instances of XXX with the appropriate details.**<br><br>• If the data are **held or will be held in a public repository**, include URLs, accession numbers or DOIs. If this information will only be available after acceptance, indicate this by ticking the box below. For example: *All XXX files are available from the XXX database (accession number(s) XXX, XXX.).*<br>• If the data are all contained **within the manuscript and/or Supporting Information files**, enter the following: *All relevant data are within the manuscript and its Supporting Information files.*<br>• If neither of these applies but you are able to provide **details of access elsewhere**, with or without limitations, please do so. For example:<br><br>*Data cannot be shared publicly because of [XXX]. Data are available from the XXX Institutional Data Access / Ethics Committee (contact via XXX) for researchers who meet the criteria for access to confidential data.*<br><br>*The data underlying the results presented in the study are available from (include the name of the third party*	All relevant data are within the manuscript and its Supporting Information files.

and contact information or URL).<br>• This text is appropriate if the data are owned by a third party and authors do not have permission to share the data.<br><br>* typeset	
Additional data availability information:	

The Editor
PLOS|ONE
27 December 2024

Dear Editor,
Re: Submission of original article for consideration by PLOS|ONE

Thank you for reviewing and providing feedback on research article submission “Impact of COVID-19 vaccination on COVID-19 hospitalisation, hospital related utilisation and expenditure: A retrospective cohort analysis of a South African private health insured population”. We found the feedback most helpful and hope that concerns raised have been addressed in the revised paper.

For the earlier revision, we were requested to provide an updated funding statement and competing interests statement in our cover letter. For completeness, please find them below.

Amended funding statement:

The South African Medical Research Council provided support in the form of salaries for authors Geetesh Solanki, and the University of Cape Town for Francesca Little and Susan Cleary. None of these institutions had any additional role in the study design, data collection and analysis, decision to publish, or preparation of the manuscript. The specific roles of these authors are articulated in the ‘author contributions’ section.”

Geetesh Solanki is also employed on a contractual basis by NMG Consultants and Actuaries, an independent consulting firm providing consulting and actuarial services to South African private health insurance funds. NMG provided access to the data used for the study but did not have any other role or involvement in the study design, data collection and analysis, decision to publish, or preparation of the manuscript. Geetesh Solanki’s commercial affiliation to NMG therefore played no role in influencing the study.

Amended competing interests statement:

Geetesh Solanki is employed on a contractual basis by the South African Medical Research Council and NMG Consultants and Actuaries, an independent consulting firm providing consulting and actuarial services to South African private health insurance funds. NMG provided access to the data used for the study but did not have any other role or involvement in the study design, data collection and analysis, decision to publish, or preparation of the manuscript and Geetesh Solanki’s commercial affiliation to NMG therefore played no role in influencing the study. Geetesh Solanki’s association with NMG does not alter our adherence to PLOS One policies on sharing data and materials.

Please also note that as part of the revised submission, we have re-uploaded as supplementary files the two final datasets: (1) Hospitalisation risk and (2) Hospital utilisation and expenditures that were used for the analysis.

We trust that concerns that were raised in the review process have been addressed in the revised paper.

Thank you for your consideration of this manuscript. Please feel free to contact us if you require additional information.

Yours sincerely
Geetesh Solanki, Susan Cleary, Francesca Little

The Editor
PLOS|ONE
31 December 2024

Dear Editor,
Re: Submission of original article for consideration by PLOS|ONE

Thank you for reviewing and providing feedback on research article submission "Impact of COVID-19 vaccination on COVID-19 hospitalisation, hospital related utilisation and expenditure: A retrospective cohort analysis of a South African private health insured population". We found the feedback most helpful and hope that concerns raised have been addressed in the revised paper.

For the earlier revision, we were requested to provide an updated funding statement and competing interests statement in our cover letter. For completeness, please find them below.

Amended funding statement:

The South African Medical Research Council provided support in the form of salaries for authors Geetesh Solanki, and the University of Cape Town for Francesca Little and Susan Cleary. None of these institutions had any additional role in the study design, data collection and analysis, decision to publish, or preparation of the manuscript. The specific roles of these authors are articulated in the 'author contributions' section."

Geetesh Solanki is also employed on a contractual basis by NMG Consultants and Actuaries, an independent consulting firm providing consulting and actuarial services to South African private health insurance funds. NMG provided access to the data used for the study but did not have any other role or involvement in the study design, data collection and analysis, decision to publish, or preparation of the manuscript. Geetesh Solanki's commercial affiliation to NMG therefore played no role in influencing the study.

Amended competing interests statement:

Geetesh Solanki is employed on a contractual basis by the South African Medical Research Council and NMG Consultants and Actuaries, an independent consulting firm providing consulting and actuarial services to South African private health insurance funds. NMG provided access to the data used for the study but did not have any other role or involvement in the study design, data collection and analysis, decision to publish, or preparation of the manuscript and Geetesh Solanki's commercial affiliation to NMG therefore played no role in influencing the study. Geetesh Solanki's association with NMG does not alter our adherence to PLOS One policies on sharing data and materials.

Please also note that as part of the revised submission, we have re-uploaded as supplementary files the two final datasets: (1) Hospitalisation risk and (2) Hospital

utilisation and expenditures that were used for the analysis. No further changes have been to the datasets and they are exactly the same as accepted as part of the first review.

We trust that concerns that were raised in the review process have been addressed in the revised paper.

Thank you for your consideration of this manuscript. Please feel free to contact us if you require additional information.

Yours sincerely
Geetesh Solanki, Susan Cleary, Francesca Little

Manuscript
Click here to access/download;Manuscript;Revised Manuscript 2024_Dec_27 without track changes.docx

Impact of COVID-19 vaccination on hospitalization, hospital utilization and expenditure for COVID-19: a retrospective cohort analysis of a South African private health insured population.

Geetesh Solanki[1, 2, 3], Susan Cleary[2,] Francesca Little[4],

Author affiliations

[1] Health Systems Research Unit, South African Medical Research Council

[2] Health Economics Unit, School of Public Health, University of Cape Town, Cape Town, South Africa

[3] NMG Consultants and Actuaries, Cape Town, South Africa

[4] Department of Statistical Sciences, University of Cape Town

Corresponding author: Geetesh Solanki

Email: geetesh.solanki@mrc.ac.za

South African Medical Research Council, Francie van Zijl Drive, Parow Valley | Cape Town | Western Cape, South Africa.

Abstract

This study quantifies the impact of COVID-19 vaccination on hospitalization for COVID-19 infection in a South African private health insurance population. This retrospective cohort study is based on the analysis of demographic and claims records for 550,332 individuals belonging to two health insurance funds between 1 March 2020 and 31 December 2022. A Cox Proportional Hazards model was used to estimate the impact of vaccination (non-vaccinated, partly vaccinated, fully vaccinated) on COVID-19 hospitalization risk; and zero-inflated negative binomial models were used to estimate the impact of vaccination on hospital utilization and hospital expenditure for COVID-19 infection, with adjustments for age, sex, comorbidities and province of residence. In comparison to the non-vaccinated, the hospitalization rate for COVID-19 was 94.51% (aHR 0.06, 95%CI 0.06, 0.07) and 93.49% (aHR 0.07, 95%CI 0.06, 0.07) lower for the partly and fully vaccinated respectively; hospital utilization was 17.70% (95% CI 24.78%, 9.95%) and 20.04% (95% CI 28.26%, 10.88%) lower; the relative risk of zero hospital days was 4.34 (95% CI 4.02, 4.68) and 18.55 (95% CI 17.12, 20.11) higher; hospital expenditure was 32.83% (95% CI 41.06%, 23.44%) and 55.29% (95% CI 61.13%, 48.57%) lower; and the relative risk of zero hospital expenditure was 4.38 (95% CI 4.06, 4.73) and 18.61 (95% CI 17.18, 20.16) higher for the partly and fully vaccinated respectively. Taken together, findings indicate that all measures of hospitalization for COVID-19 infection were significantly lower in the partly or fully vaccinated in comparison to the non-vaccinated. The use of real-world data and an aggregated level of analysis resulted in the study having several limitations. While the overall results may not be generalizable to other populations, the findings add to the evidence based on the impact of COVID-19 vaccination during the period of the pandemic.

Introduction

The World Health Organization (WHO) declared ~~COVID-19~~ a Public Health Emergency of International Concern and a pandemic on 30 January and 11 March 2020 respectively [1, 2], triggering an international response to develop a COVID-19 vaccine with four vaccine candidates entering human evaluation by March 2020[3]. South Africa commenced its national COVID-19 vaccination program on 17 February 2021[4]. By 31 December 2023, based on the WHO COVID-19 dashboard, South Africa had a total of 4.1 million confirmed COVID-19 cases and 70 vaccination doses per 100 population with 41 per 100 population completing the primary series and 7 per 100 population receiving at least one additional dose or booster [5]. While it is likely that COVID-19 deaths were underreported, between December 2019 and January 2022, there were 295,135 excess natural deaths in South Africa with evidence suggesting that most were due to COVID-19 [6].

While there is now substantial global evidence on the impact of COVID-19 vaccines, research in African regions remains limited. Societies in these regions have relatively poor access to health care services, varying levels of vaccine awareness and sub-optimal vaccine coverage with vaccine hesitancy emerging as an obstacle to the uptake of COVID-19 vaccines during the pandemic [7]. In South Africa, evidence confirms that the risk of COVID-19 related morbidity and mortality varied across waves and COVID-19 variants, by age, sex, level of comorbidity, , and whether dependent on public or private health care[8, 9]. Studies have also confirmed the efficacy of specific COVID-19 vaccines against specific COVID-19 variants [10-13] and the effectiveness of the vaccine in “real world” situations [12, 14]. However, gaps in knowledge remain regarding the overall “real world” impact of COVID-19 vaccination as a strategy to manage COVID-19 related morbidity and health care expenditure during the pandemic.

Using Real World Data (RWD), the aim of this paper is to quantify the impact of COVID-19 vaccination on hospitalization, hospital utilization and hospital expenditure for COVID-19 infection, based on data from

a South African private health insured population cohort over the entire period of the COVID-19 pandemic.

Methods

Study design, population and period

Strengthening and Reporting of Observational Studies in Epidemiology (STROBE) guidelines were used to conduct and report on this study[15]. The study is a retrospective cohort analysis evaluating the impact of COVID-19 vaccination on hospitalization, utilization and expenditure for COVID-19 infection. The study population included 550,332 individuals who were enrolled in one of two large health insurance funds during the study period of 1 March 2020 to 31 December 2022, corresponding to the first reported case of COVID-19 in South Africa on 5 March 2020 [16] and the end of the fourth wave and removal or COVID-19 restrictions on 30 December 2022[17]. For individuals who were members of these insurance funds for the entire pandemic, their individual study period corresponded to the overall study period; while the actual date of entry or exit was used for those who joined or left one of the funds during the pandemic. This population represents approximately 5% of the South African private health insurance population and is comparable to the broader insured population in terms of family size, health insurance contributions and health care expenditure (Table S1)[18].

Exposures and outcomes of interest

The exposure of interest was defined as non-vaccinated, partly vaccinated or fully vaccinated. The South African government authorized the Johnson and Johnson (J&J) and the Pfizer BioNtech vaccines. In line with the literature [19] as well as the approach used in South Africa [20], individuals were initially categorized as non-vaccinated, becoming partly vaccinated the day after their first vaccine dose (J&J or Pfizer BioNtech), and fully vaccinated 28 days after their first J&J or 14 days after the second dose of Pfizer BioNtech. Individuals receiving booster vaccinations remained in the fully vaccinated category.

Outcomes were defined as the risk of COVID-19 hospitalization (individual having a hospital admission or not), the extent of COVID-19 hospital utilization (number of days in hospital) and the extent of COVID-19 hospital expenditure. COVID-19 hospital admissions were identified using ICD-10 code (10th revision of the International Statistical Classification of Diseases and Related Health Problems) U07.1 or U07.2[21]. For these admissions, hospital utilization was defined using data on the date of admission and date of discharge or death in hospital. Total hospital and related expenditures took an insurer perspective and reflected the amounts paid by the health insurance funds to cover the claims submitted by health care providers for services rendered to the hospitalized individuals. These direct costs included health care providers, hospital bed days, as well as any pharmaceuticals, medical or surgical consumables dispensed. All expenditures in South African Rands (ZAR) were converted to United States Dollar ($) values using the December $/ZAR exchange rate for the given year and then inflated/deflated to be expressed in 2022 values using the South African Consumer Price Index[22].

In addition to the above exposures and outcomes, the analysis incorporated sex (male, female), age (less than 18, between 18 and 25, between 25 and 40, between 40 and 65 and greater than 65), COVID-19

comorbidities (yes, no) and province of residence (Eastern Cape, Free State, Gauteng, KwaZulu-Natal, Limpopo, Mpumalanga, North West, Northern Cape, Western Cape) as predictor variables. Comorbidities for COVID 19 considered in the analysis included cancers, chronic renal disease, congestive cardiac failure, chronic obstructive pulmonary disease, diabetes mellitus, ~~HIV~~, hypercholesterolaemia, hypertension, hypothyroidism, ischaemic heart disease, pregnancy and tuberculosis, given that these have been identified as being important comorbidities for COVID-19 [23]. The individuals with these COVID-19 comorbidities were identified using Council for Medical Schemes (CMS) Guidelines for identifying these conditions using claims records [24].

Data Collection

Data for the study were extracted from the data warehouse of NMG Consultants and Actuaries (NMG), an independent consulting firm providing consulting and actuarial services to the two health insurance funds. Electronic demographic and claims records for the insured individuals, stored on a Microsoft SQL Server in the NMG data warehouse, were extracted in comma-separated values (CSV) format and then imported into STATA/SE 16.1[25] for data cleaning, descriptive analyses, and statistical modeling.

The approach used to extract and classify the data is schematically summarized in Figure 1. From all the data for the period, a 3-step approach was used to extract and compile the data into various datasets.

Fig 1. Approach to data extraction

In the first step, three data files were created. The first “demographic” file consisted of a record per individual who belonged to either of the two health insurance funds at any time over the study period

(unique study ID, date of joining and exiting the fund, age, sex, province of residence, and comorbidities). The second “COVID-19 vaccination status” data file consisted of a record for every vaccination event in an individual (unique study ID, date of change of vaccination status, vaccination status). Individuals received their vaccinations at various points in time over the study period and their vaccination status therefore changed over time.

The “COVID-19 hospital admission” data file consisted of a record for each hospital admission for COVID-19 infection (unique study ID, date of admission, date of discharge, length of stay and total hospital and related expenditure). Individuals with multiple COVID-19 admissions had separate records for each admission. For those who were hospitalized, vaccination status (not, partly and fully vaccinated) on the date of admission was assumed.

For the second step, two separate datasets were created using information from the three files described above. Each dataset included at least one record for the 550,332 individuals included in the study population. The first dataset enabled the analysis of the risk of COVID-19 hospitalization events. It included “time stamped” records for every hospitalization for COVID-19 infection or COVID-19 vaccination, together with records for all individuals who had none of these events. Multiple records were created for individuals with more than one COVID-19 hospitalization or COVID-19 vaccination event. The time stamp was created by determining the number of days from study start date (1 March 2020) for the individual to the date of the event. The second dataset enabled the analysis of hospital utilization and expenditure for COVID-19 infection. Data included the number of days spent in hospital and expenditure per hospitalization (zero if no hospital event).

Statistical analysis

For the analysis of the impact of vaccination on risk of hospitalization, summary statistics reported include frequencies and percentages for the number of individuals, and hospitalization frequencies and rates (number of hospital events per 1000 individuals per annum). For the multivariate analysis, survival analysis was used to model the impact of vaccination on the risk of hospitalization for COVID-19 infection to account for censoring due to no relevant hospitalization during the observation period. A Cox Proportional Hazards model was used to estimate the relative hazard of hospitalization for COVID-19 infection in partly or fully vaccinated versus non-vaccinated individuals. ~~To factor in the censoring of the follow-period in the analysis, the Cox PH model was used for the primary analysis of the association between vaccination status and the risk of hospitalization, The model allows for those subjects who were never hospitalized during the follow-up period to be accommodated as censored [illegible]ervations.~~

Time-varying vaccination status was incorporated through repeated measures per individual relating to the periods with different vaccination statuses. The large dataset did not allow for estimation of shared frailty models. The repeated observations per individual (individuals with multiple vaccination events and multiple hospital events) was taken into consideration by incorporating the data clustering option by individual in the modelling and hence the use of robust estimators, allowing errors to be correlated within subjects but independent between subjects. The proportional hazards assumption was assessed through Kaplan-Meier curves and found to be justifiable. The association between vaccination status and risk of hospitalization are reported as unadjusted Hazard Ratios (UHR) and adjusted Hazard Ratios (AHR) with 95% confidence intervals.

For the analysis of the impact of COVID-19 vaccination on hospital utilization for COVID-19, the summary statistics included the total number of days in hospital, number of days per hospitalization event and the number of hospital days per 1000 individuals per annum. For the expenditure analysis, the summary statistics include the total expenditure in millions ($), expenditure per hospitalization event and the expenditure per individual per annum.

For the multivariate analysis of the impact of vaccination on utilization and expenditure, zero-inflated negative binomial models were used as the exploratory analyses indicated a highly skewed distribution not only because of the zeros, but also a skewed distribution on the non-zero values. The zero-inflated negative binomial model produces two sets of outputs: the count component and the zero-inflated component. The count component examines the effects of predictor variables on the expected non-zero count of the outcome variable, while the zero-inflated component explores factors influencing the probability of the presence of excess zeros. The association between vaccination and utilization and expenditure as estimated by these models are presented as percentage increase or decrease in the number of average days/ expenditures depending on whether the coefficient from the count component of the model (e.g., beta1) is positive or negative using the formula, (exp(beta1)-1) x100, and (ii) as the risk ratio for zero hospital days in the vaccinated versus the non-vaccinated, or relative to the reference group for other predictor variables, using the formula exp(beta) where beta refers to the coefficients of the zero-inflated component of the model and their 95% confidence intervals. Both unadjusted estimates from univariate models and adjusted estimates from multivariable models are presented. The lack of independence due to repeated observations per individual (individuals with multiple hospital events) was dealt with through the estimation of robust standard errors allowing correlated errors within subject and uncorrelated errors between subjects. No specific cut-off was used to determine statistical significance, and no p-values are reported. The focus is on effect sizes and their 95% confidence intervals. STATA/SE 16.1 was used for all the analyses.

Ethical considerations

The data for the study were accessed in terms of and under the conditions set out in the agreement between NMG and the client insurance funds and between NMG and the researcher. The terms set out in these agreements ensured that the access and use of the data is in full compliance with the requirements of the Protection of Personal Information (PoPI) Act in South Africa[26]. Ethics approval for the use of the database to carry out this study was granted by the Human Research Ethics Committee of the Faculty of Health Sciences, University of Cape Town (reference number 606/2023).

Results

Characteristics of the study population

The study cohort included 550,332 individuals who belonged to the two health insurance funds at any time between 1 March 2020 and 31 December 2022 (1029 days). The average duration of membership for these individuals was 864 days, with 70% of the individuals being members of the funds for the full study period. At the end of the study period, 62.1% were non vaccinated, 12.2% were partly vaccinated, and 25.7% were fully vaccinated. Of all the individuals, 53.6% were female, 34.4% were less than 18 years old, 23.3% had one or more comorbidity, and the highest proportion (45.4%) of individuals resided in the Gauteng province (Table 1).

Table 1. Characteristics of study population

Variable	Number of individuals	
	N	%
**Vaccination Status***		
Non vaccinated	341 972	62.1%
Partly vaccinated	66 885	12.2%
Fully vaccinated	141 475	25.7%
**Sex**		
Female	295 116	53.6%
Male	255 216	46.4%
**Age**		
Less than 18	189 405	34.4%
Between 18-25	40 190	7.3%
Between 25-40	145 004	26.3%
Between 40-65	140 255	25.5%
Greater than 65	35 478	6.4%
**COVID-19 Comorbidities**		
No	422 116	76.7%
Yes	128 216	23.3%
**Province**		
Eastern Cape	40 694	7.4%
Free State	22 347	4.1%
Gauteng	249 650	45.4%
KwaZulu-Natal	85 572	15.5%
Limpopo	19 410	3.5%
Mpumalanga	18 514	3.4%
North West	19 064	3.5%
Northern Cape	10 002	1.8%
Western Cape	85 079	15.5%
**Total**	550 332	100.0%

Vaccination Status*: Status as at end of study.

Hospitalization Risk for COVID-19 infection

As shown in Table 2, the hospitalisation rate for COVID-19 infection (events per 1000 individuals per year) was 7.45 (95%CI 7.31, 7.58) in the study population; 10.29 (95%CI 10.09, 10.49) for the non-vaccinated; 4.47 (95%CI 4.17, 4.78) for the partly vaccinated and 1.97 (95%CI 1.84, 2.11) for the fully vaccinated. Across the other predictor variables, males (7.56, 95%CI 7.36, 7.76), individuals aged 65 and above (25.62, 95%CI 24.63, 26.61), those with 1 or more comorbidity (20.12, 95%CI 19.66, 20.58) and those from the Western Cape (9.02, 95%CI 8.64, 9.40) had higher hospitalization rates.

In multivariate analysis, after adjustment for all other factors, the risk of COVID-19 hospitalization was 94.51% (aHR 0.06, 95%CI 0.06, 0.07) lower for the partly vaccinated and 93.49% (aHR 0.07, 95%CI 0.06, 0.07) lower for the fully vaccinated when compared with the non-vaccinated. The risk of hospitalization was higher for males (aHR 1.17, 95%CI 1.13, 1.12), those aged 65 and above (aHR 17.27, 95% CI 15.78, 18.89), and those with 1 or more comorbidity (aHR 3.33, 95%CI 3.18, 3.50). Individuals from all other provinces had a higher risk of COVID-19 hospitalization than individuals from the Eastern Cape, the reference group.

Table 2. Univariate and multivariate analysis of impact of COVID-19 vaccination on risk of hospitalization for COVID-19 infection

Variable	# of Hosp Events		Hazard Ratio for Hospitalization	
	N	Events/1000 Individuals/ per annum**	UHR (95% CI)	AHR (95% CI)
**Vaccination Status***				
Non vaccinated	9,972	10.29	Reference	Reference
Partly vaccinated	848	4.47	0.17 (0.15, 0.18)	0.06 (0.06, 0.07)
Fully vaccinated	791	1.97	0.20 (0.19, 0.22)	0.07 (0.06, 0.07)
**Sex**				
Female	6,145	7.35	Reference	Reference
Male	5,466	7.56	1.05 (1.01, 1.09)	1.17 (1.13, 1.22)
**Age**				
Less than 18	927	1.73	Reference	Reference
Between 18-25	271	2.38	1.33 (1.16, 1.53)	1.99 (1.73, 2.29)
Between 25-40	2,407	5.86	3.49 (3.23, 3.78)	5.04 (4.66, 5.46)
Between 40-65	5,431	13.67	7.64 (7.11, 8.22)	10.32 (9.55, 11.14)
Greater than 65	2,575	25.62	13.93 (12.84, 15.11)	17.27 (15.78, 18.89)
**COVID-19 Comorbidities**				
No	4,303	3.60	Reference	Reference
Yes	7,308	20.12	4.94 (4.74, 5.15)	3.33 (3.18, 3.50)
**Province**				
Eastern Cape	827	7.17	Reference	Reference
Free State	461	7.28	1.08 (0.95, 1.22)	1.26 (1.12, 1.43)
Gauteng	4,812	6.80	0.95 (0.88, 1.03)	1.33 (1.22, 1.44)
KwaZulu-Natal	2,084	8.60	1.21 (1.11, 1.32)	1.30 (1.19, 1.42)
Limpopo	271	4.93	0.74 (0.64, 0.85)	1.04 (0.90, 1.21)
Mpumalanga	320	6.10	0.90 (0.78, 1.03)	1.02 (0.88, 1.17)
North-West	419	7.76	1.14 (1.00, 1.30)	1.33 (1.17, 1.52)
Northern Cape	242	8.54	1.27 (1.09, 1.48)	1.42 (1.22, 1.66)
Western Cape	2,175	9.02	1.26 (1.15, 1.37)	1.32 (1.21, 1.44)
**Total**	11,611	7.45		

Vaccination Status*: Status at time of hospital event.

Events/1000 Individuals/per annum**: Total events/ number of individuals/study period (2.83 years) *1000

Hospital Utilization for COVID-19 infection

Overall hospital utilization for COVID-19 infection was 69.22 (95%CI 66.87, 71.50) days per 1000 individuals per year (Table 3). The utilization rate was 93.82 (95%CI 90.31,96.72) for the non-vaccinated, 44.11 (95%CI 37.06, 48.21) for the partly vaccinated and 20.52 (95%CI 17.25, 24.69) for the fully vaccinated. Across the other predictor variables, males (73.78, 95%CI: 70.21, 77.26), individuals aged 65 and above (322.4, 95%CI 295.21, 344.52), those with comorbidities (214.87, 95%CI 204.78, 222.94) and those from the Western Cape (86.56, 95%CI 79.41, 93.82) had the highest hospital utilization rates.

In multivariate analysis, after adjustment for all other factors, the results from the count component of the model indicated that compared with the non-vaccinated, hospital utilization was 17.70% (95% CI 24.78%, 9.95%) lower for the partly vaccinated and 20.04% (95% CI 28.26%, 10.88%) lower for the fully-vaccinated. The zero inflated component results indicated that, compared with the non-vaccinated, the relative risk of zero hospital days was 4.34 (95% CI 4.02, 4.68) for the partly vaccinated increasing to 18.55 (95% CI 17.12, 20.11) for the fully vaccinated. Across the other predictor variables, the results from the count component indicated that hospital utilization was 10.87% (95% CI 5.38%, 16.64%) higher for males compared with females, 198.39% (95% CI 156.51%, 247.10%) higher for those greater than 65-year-old compared with under 18's, and 24.82% (95% CI 17.99%, 32.05%) higher for those with comorbidities compared with those without. The zero inflated component results indicated that the relative risk of zero hospital days was 0.87 (95% CI 0.84, 0.91) for males compared with females, 0.06 (95% CI 0.06, 0.07) for those greater than 65-year-old compared with the under 18's, 0.26 (95% CI 0.25, 0.27) for those with a comorbidity compared to those without and 0.74 (95% CI 0.68, 0.82) for those from the Western Cape relative to the Eastern Cape.

Table 3. Univariate and multivariate analysis of impact of COVID-19 vaccination on hospital utilization for COVID-19 infection

	Hospital Days			% Difference in Hospital Days vs Reference Group***		Risk Ratio of Zero Hospital Day vs. Reference	
	Number of days	Days/ Event	Days/ 1000 individuals/ year**	Unadjusted	Adjusted	Unadjusted	Adjusted
**Vaccination Status***							
Non vaccinated	91 738	9.19	93.82	Reference	Reference	Reference	Reference
Partly vaccinated	7 976	9.41	44.11	-0.54% (-9.26%, 9.02%)	-17.70% (-24.78%, -9.95%)	2.29 (2.12, 2.47)	4.34 (4.02, 4.68)
Fully vaccinated	8 219	10.39	20.52	0.17% (-10.39%, 11.98%)	-20.04% (-28.26%, -10.88%)	5.19 (4.80, 5.62)	18.55 (17.12, 20.11)
**Sex**							
Female	54 582	8.88	65.28	Reference	Reference	Reference	Reference
Male	53 351	9.76	73.78	11.90% (6.27%, 17.83%)	10.87% (5.38%, 16.64%)	0.98 (0.94, 1.02)	0.87 (0.84, 0.91)
**Age**							
Less than 18	3 870	4.17	7.21	Reference	Reference	Reference	Reference
Between 18-25	1 479	5.46	12.99	39.54% (6.54%, 82.77%)	38.10% (7.47%, 77.45%)	0.77 (0.67, 0.90)	0.57 (0.49, 0.66)
Between 25-40	15 518	6.45	37.77	69.91% (45.38%, 98.58%)	65.55% (42.17%, 92.77%)	0.32 (0.29, 0.35)	0.25 (0.23, 0.27)
Between 40-65	54 658	10.06	137.54	176.56% (138.65%, 220.50%)	152.54% (118.19%, 192.29%)	0.14 (0.13, 0.15)	0.12 (0.11, 0.13)
Greater than 65	32 408	12.59	322.40	228.82% (182.87%, 282.24%)	198.39% (156.51%, 247.10%)	0.07 (0.07, 0.08)	0.06 (0.06, 0.07)
**COVID-19 Comorbidities**							
No	29 875	6.94	24.98	Reference	Reference	Reference	Reference
Yes	78 058	10.68	214.87	55.32% (47.18%, 63.91%)	24.82% (17.99%, 32.05%)	0.18 (0.17, 0.19)	0.26 (0.25, 0.27)
**Province**							
Eastern Cape	8 175	9.89	70.90	Reference	Reference	Reference	Reference
Free State	4 449	9.65	70.27	-2.58% (-16.17%, 13.21%)	-0.18% (-14.02%, 15.89%)	0.98 (0.86, 1.11)	0.84 (0.74, 0.96)
Gauteng	46 851	9.74	66.24	-6.42% (-14.71%, 2.68%)	-0.88% (-9.52%, 8.60%)	1.05 (0.96, 1.13)	0.76 (0.70, 0.83)
KwaZulu-Natal	17 369	8.33	71.64	-19.14% (-27.01%, -10.42%)	-11.32% (-19.85%, -1.89%)	0.81 (0.74, 0.88)	0.74 (0.67, 0.81)
Limpopo	1 981	7.31	36.02	-28.91% (-42.39%, -12.27%)	-18.05% (-31.78%, -1.55%)	1.40 (1.21, 1.63)	0.97 (0.83, 1.13)
Mpumalanga	2 816	8.80	53.68	-12.15% (-27.30%, 6.15%)	-5.67% (-20.69%, 12.21%)	1.16 (1.01, 1.33)	0.98 (0.85, 1.13)
North West	3 441	8.21	63.70	-18.76% (-29.19%, -6.78%)	-16.46% (-26.91%, -4.53%)	0.90 (0.79, 1.03)	0.75 (0.66, 0.86)
Northern Cape	1 986	8.21	70.08	-18.82% (-32.45%, -2.45%)	-16.05% (-30.38%, 1.24%)	0.81 (0.70, 0.95)	0.70 (0.60, 0.83)
Western Cape	20 865	9.59	86.56	-6.88% (-16.40%, 3.72%)	-8.83% (-17.66%, 0.95%)	0.78 (0.72, 0.85)	0.74 (0.68, 0.82)
**Total**	107 933	9.30	69.22				

Vaccination Status*: Status as time of hospital event if hospitilised, and as end of study period if never hospitilised during study period

Days/1000 Individuals/per annum**: Total days/ number of individuals/study period (2.83 years) *1000

% Difference in hospital days***: Exponent of estimated coefficents (beta1) from count component output from negative binonmial model - 1 X 100: (exp(beta1)-1)x100.

Risk Ratio for zero hospital days****: Exponent of estimated coefficents (beta1) from zero inflated component output from negative binonmial model : exp(beta1)

Hospital Expenditure for COVID-19 infection

For the overall study population, hospital expenditure per individual for COVID-19 infection was $12.39 (95%CI $11.15, $13.61) (Table 4), with $17.85 (95%CI $16.81, $18.77) per annum for the non-vaccinated, $6.19 (95%CI $5.20, $6.76) for the partly vaccinated, and $1.86 (95%CI $1.62, $2.18) for fully vaccinated individuals. Across the other predictor variables, males ($13.25, 95%CI $12.26, $14.23), individuals aged 65 and above ($110.17, 95%CI $109.18, $111.15), those with comorbidities ($37.57, 95%CI $36.59, $38.55) and those from the Western Cape ($16.22, 95%CI $15.23, $17.21) had the highest hospital expenditure for COVID-19 infection.

In multivariate analysis, after adjustment for all other factors, the results from the count component of the model indicated that hospital expenditure was 32.83% (95% CI 41.06%, 23.44%) lower for the partly vaccinated and 55.29% (95% CI 61.13%, 48.57%) lower for the fully vaccinated when compared with the non-vaccinated. Hospital expenditure was 9.62% (95% CI 0.37%, 18.57%) higher for males compared with females, 259.12% (95%CI 195.45%, 337.44%) higher for those aged 65 and above compared with under 18's and 19.43% (95%CI 8.29%, 31.71%) higher for those with comorbidities compared to those without. The zero inflated component results indicated that, compared with the non-vaccinated, the relative risk of zero hospital expenditure was 4.38 (95% CI 4.06, 4.73) for the partly vaccinated and 18.61 (95% CI 17.18, 20.16) for the fully vaccinated compared with the non-vaccinated. Across the other predictor variables, the results from the count component indicated that hospital expenditure was 9.75% (95% CI 0.86%, 19.43%) higher for males compared with females, 257.83% (95% CI 194.09%, 335.40%) higher for those aged greater than 65-year-old compared with the under 18's, 19.44% (8.31%, 31.72%) higher for those with comorbidities compared to those without and 38.59% (95% CI 17.77%, 63.10%) higher for those from the Western Cape compared to those from the Eastern Cape. The zero inflated component results indicated that the relative risk of zero hospital expenditure was 0.86 (95% CI 0.83, 0.90) for males compared with females, 0.06 (95% CI 0.05, 0.06) for those aged greater than 65-year-old compared with the under 18's, 0.25 (0.24, 0.27) for those with comorbidities compared to those without, and 0.75 (95% CI 0.69, 0.83) for those from the Western Cape compared with the Eastern Cape.

Table 4. Univariate and multivariate analysis of impact of COVID-19 vaccination on hospital expenditure for COVID-19 infection.

	Hospital Expenditure**			% Difference in Hospital expenditure vs Reference Group***		Risk Ratio of Zero Hospital Expenditure vs.	
	US Dollars(m's)	US Dollars /Event	US Dollars/ Individual/ Annum***	Unadjusted	Adjusted	Unadjusted	Adjusted
**Vaccination Status***							
Non vaccinated	17.45	1 749	17.85	Reference	Reference	Reference	Reference
Partly vaccinated	1.12	1 319	6.19	-25.30% (-34.09%, -15.33%)	-32.83% (-41.06%, -23.44%)	2.28 (2.11, 2.46)	4.38 (4.06, 4.73)
Fully vaccinated	0.75	943	1.86	-48.35% (-55.11%, -40.57%)	-55.29% (-61.13%, -48.57%)	5.15 (4.76, 5.56)	18.61 (17.18, 20.16)
**Sex**							
Female	9.74	1 585	11.65	Reference	Reference	Reference	Reference
Male	9.58	1 752	13.25	10.77% (0.69%, 21.86%)	9.75% (0.86%, 19.43%)	0.97 (0.93, 1.01)	0.86 (0.83, 0.90)
**Age**							
Less than 18	0.47	506	0.87	Reference	Reference	Reference	Reference
Between 18-25	4.58	16 917	40.26	60.42% (2.42%, 151.26%)	68.23% (15.25%, 145.57%)	0.73 (0.64, 0.84)	0.54 (0.47, 0.62)
Between 25-40	0.22	91	0.53	146.40% (100.00%, 203.56%)	142.25% (99.61%, 194.00%)	0.29 (0.27, 0.32)	0.23 (0.21, 0.25)
Between 40-65	2.97	547	7.48	308.13% (237.32%, 393.80%)	286.26% (222.83%, 362.17%)	0.12 (0.12, 0.13)	0.11 (0.10, 0.11)
Greater than 65	11.07	4 301	110.17	254.45% (189.76%, 333.59%)	257.83% (194.09%, 335.40%)	0.06 (0.06, 0.07)	0.06 (0.05, 0.06)
**COVID-19 Comorbidities**							
No	5.67	1 317	4.74	Reference	Reference	Reference	Reference
Yes	13.65	1 868	37.57	41.64% (28.51%, 56.12%)	19.44% (8.31%, 31.72%)	0.17 (0.16, 0.18)	0.25 (0.24, 0.27)
**Province**						Reference	
Eastern Cape	1.07	1 298	9.31	Reference	Reference		Reference
Free State	0.72	1 568	11.42	21.42% (-6.59%, 57.83%)	27.82% (-3.88%, 69.96%)	0.99 (0.87, 1.12)	0.85 (0.75, 0.96)
Gauteng	8.03	1 668	11.35	28.58% (8.92%, 51.79%)	33.42% (14.57%, 55.38%)	1.05 (0.97, 1.14)	0.77 (0.71, 0.83)
KwaZulu-Natal	3.90	1 869	16.07	44.15% (20.02%, 73.14%)	49.79% (26.43%, 77.46%)	0.83 (0.76, 0.91)	0.75 (0.69, 0.82)
Limpopo	0.25	914	4.50	-29.88% (-51.05%, 0.45%)	-23.55% (-43.95%, 4.28%)	1.45 (1.25, 1.68)	0.99 (0.85, 1.15)
Mpumalanga	0.41	1 277	7.79	-1.71% (-28.12%, 34.42%)	2.38% (-23.02%, 36.15%)	1.17 (1.02, 1.35)	0.99 (0.85, 1.14)
North West	0.60	1 435	11.13	10.62% (-11.74%, 38.64%)	4.30% (-14.95%, 27.90%)	0.92 (0.81, 1.05)	0.77 (0.67, 0.88)
Northern Cape	0.43	1 774	15.15	36.96% (-5.53%, 98.55%)	45.66% (-5.35%, 124.16%)	0.83 (0.71, 0.98)	0.72 (0.61, 0.85)
Western Cape	3.91	1 798	16.22	38.38% (15.70%, 65.50%)	38.59% (17.77%, 63.10%)	0.79 (0.72, 0.86)	0.75 (0.69, 0.83)
**Total**	19.32	1 664	12.39				

Vaccination Status*: Status as time of hospital event if hospitilised, and as end of study period if never hospitilised during study period

Hospital Expenditure**: South African Rands converted to US Dollary terms and discounted to 2022 value

USD/Individual/per annum***: Total USD/ number of individuals/study period (2.83 years)

% Difference in hospital expenditure***: Exponent of estimated coefficents (beta1) from count component output from negative binonmial model - 1 X 100: (exp(beta1)-1)x100.

Risk Ratio for zero hospital expenditure****: Exponent of estimated coefficents (beta1) from zero inflated component output from negative binonmial model : exp(beta1)

Discussion

This study has estimated the impact of COVID-19 vaccination on hospitalization for COVID-19 infection based on a retrospective cohort analysis of 550,332 individuals enrolled in two private health insurance schemes during the period of the pandemic. In contrast to many studies that have reported on the efficacy of the vaccine in clinical trial situations, in population sub-sets using test-negative controls[27],

on specific COVID-19 variants and/or vaccines, this study provides insights on the impact of COVID-19 vaccination on an aggregated basis in a “real world” population.

By the end of the study period, 12.2% of the study cohort was partly vaccinated and 25.7% was fully vaccinated. The overall hospitalization rate for COVID-19 infection was 7.45 events per 1000 individuals per annum, hospital utilization was 69.22 days per 1000 individuals per annum, and hospital expenditure was $12 per individual per annum.

Across all analyses and after adjustment for other risk factors, the study found significantly lower hospitalization events, hospital utilization and hospital expenditure for COVID-19 infection in the partly or fully vaccinated in comparison to the non-vaccinated. Each outcome will be discussed in turn.

First, the study finds that hospitalization risk for COVID-19 infection was 94.51% (aHR 0.06, 95%CI 0.06, 0.07) lower for the partly vaccinated and 93.49% (aHR 0.07, 95%CI 0.06, 0.07) lower for the fully vaccinated. These findings are in line with existing literature. A South African study examining the impact of the J&J vaccine in health workers reported vaccine effectiveness of 67% (95% CI: 62%-71%) in preventing COVID-19-related hospitalization[12]. More broadly, at least 3 meta- analyses have reported similar results. The first, including 51 studies assessing vaccine effectiveness in the “real-world” found that COVID-19 vaccines were 97.2% (95% CI 96.1-98.3%) effective in preventing hospitalization [28]. The second, a review of 58 studies, reported a single dose of vaccine to be 66% (50%–81%) effective while two doses were 93% (89%–96%) effective in preventing hospitalization[29]. The third, a systematic review of 42 studies examining the efficacy of a range of COVID-19 vaccines in a variety of populations, concluded that the Pfizer/BioNTech vaccine had >90% effectiveness against infections requiring hospitalization after the second dose. A single dose of the J&J vaccine was >60% effective against infections requiring hospitalization[30].

Second, in terms of hospital utilization for COVID-19 infection, findings indicate that utilization was 17.70% (95% CI -24.78%, -9.95%) lower for the partly vaccinated and 20.04% (95% CI -28.26%, -10.88%) lower for the fully vaccinated; while the relative risk of zero hospital days was 4.34 (95% CI 4.02, 4.68) higher for the partly vaccinated and 18.55 (95% CI 17.12, 20.11) higher for the fully vaccinated. This compares to a reported reduction in the length of hospital stay of 38% (95% CI: 19%–52%) in a Norwegian study [31], and the 14% (95 %CI -24%, 4%) reduction reported in a Chinese study [32].

Third, in terms of hospital expenditure for COVID-19 infection, findings indicate that expenditure was 32.83%(95% CI 41.06%, 23.44%) lower for the partly vaccinated and 55.29% (95% CI 61.13%, 48.57%) lower for the fully vaccinated; and the relative risk of zero hospital expenditure was 4.38 (95% CI 4.06, 4.73) higher for the partly vaccinated and 18.61 (95% CI 17.18, 20.16) higher for the fully vaccinated. In the only study we were able to find reporting on the impact of vaccination on hospital expenditure, carried out at a large academic medical center in the United States of America, vaccinated patients experienced a 26% lower cost of hospitalization compared with the non-vaccinated (P = 0.004). [33].

This study has several limitations. While the results of the study are arguably generalizable to the insured population, generalizability to the broader South African population is limited given differences in demographics, socio-economic profiles, access to care and clinical guidelines between the insured and non-insured populations. The analysis uses routine “real world” demographic and claims data collected by the health insurance funds as part of their business, not data collected for research purposes ~~pe~~ se. Only services and vaccines for which claims were submitted were analysed. The implications are that both COVID-19 vaccination events and hospitalization for COVID-19 infection could be over or underreported. While it is hard to predict the impact of these biases, it seems plausible that they would not negate the findings entirely, given the magnitude of the impact. In addition, the available demographic and claims data did not enable an analysis of severity of COVID-19 infection. ~~Survival analysis or COVID-19 related mortality related assessments were also not possible~~ due to limitations of

the mortality data. Although rare, evidence now indicates concerns around safety and side-effects of COVID-19 vaccines[34, 35]. These were not taken into consideration in this study. Given that this is a retrospective observational cohort study, subject loss-to-follow up was not a concern. However, the observational nature of the study may have resulted in selection and confounding biases. Caution should therefore be exercised in interpreting the findings of this study.

Conclusion

This study, based on a large cohort of insured individuals followed over the entire period of the pandemic, found that - all other things being equal - the risk of hospitalization, hospital utilization and expenditure for COVID-19 infection was substantially lower amongst those who had vaccinated for COVID-19. The study provides evidence on the overall "real-world" impact of COVID-19 vaccination, particularly in insured populations and those in Sub-Saharan Africa. Given the differences in demographics, socio-economic profiles, and access to care between insured and non-insured populations, caution should be exercised in generalizing the findings of this study.

Financial disclosure

The South African Medical Research Council provided support in the form of salaries for authors Geetesh Solanki, and the University of Cape Town for Francesca Little and Susan Cleary. None of these institutions had any additional role in the study design, data collection and analysis, decision to publish, or preparation of the manuscript. The specific roles of these authors are articulated in the 'author contributions' section." Geetesh Solanki is also employed on a contractual basis by NMG Consultants and Actuaries, an independent consulting firm providing consulting and actuarial services to South African private health insurance funds. NMG provided access to the data used for the study but did not have any other role or involvement in the study design, data collection and analysis, decision to publish, or preparation of the manuscript. Geetesh Solanki's commercial affiliation to NMG therefore played no role in influencing the study.

Competing Interests

Geetesh Solanki is employed on a contractual basis by the South African Medical Research Council and NMG Consultants and Actuaries, an independent consulting firm providing consulting and actuarial services to South African private health insurance funds. NMG provided access to the data used for the study but did not have any other role or involvement in the study design, data collection and analysis, decision to publish, or preparation of the manuscript and Geetesh Solanki's commercial affiliation to NMG therefore played no role in influencing the study. Geetesh Solanki's association with NMG does not alter our adherence to PLOS One policies on sharing data and materials.

Ethics Statement

Ethics approval for the use of the secondary database used to carry out this study was granted by the Faculty of Health Sciences Human Research Ethics Committee of the University of Cape Town – approval number HREC Ref: 606/2023. No individual consent was required as the research was based on anonymised secondary claims data. Data for the study were made available as part of NMG Consultants and Actuaries (NMG) commitment to support research initiatives with broader public health significance. The data were accessed in terms of and under the conditions set out in the agreement between NMG and the client schemes and between NMG and the researcher. The terms set out in these agreements ensured that the access to and use of the data is in full compliance with the requirements of the Protection of Personal Information (PoPI) Act in South Africa [42]. All the data were provided in a de-identified format and aggregated at an individual level and the research team had no access to information that would enable the identification of any individual. All findings are presented at an aggregate level and no confidential member, health care provider or scheme information is disclosed. Individual consent for the data was therefore not required.

Author contributions

Geetesh Solanki designed the study, Francesca Little lead the data analysis and Susan Cleary provided critical review oversight. Geetesh Solanki provided guidance in extracting the data from the NMG database. Geetesh Solanki wrote the first draft of the manuscript which was then critically reviewed and revised by the other authors. The corresponding author attests that all listed authors meet authorship criteria and that no others meeting the criteria have been omitted. Geetesh Solanki is the guarantor.

Data Availability

Yes - all data are fully available without restriction. All relevant data are within the manuscript and its Supporting Information files.

Acknowledgements

The authors would like to acknowledge the institutional support to the study authors provided by the South African Medical Research Council (SAMRC). We also acknowledge NMG Consultant and Actuaries for assisting in providing access to the data, to Priya Makanjee for extracting the data, and to Jud Cornell for assistance in fine editing the script.

References

1. Statement on the second meeting of the International Health Regulations (2005) Emergency Committee regarding the outbreak of novel coronavirus (2019-nCoV) [Internet]. Geneva WHO; 2020
2. WHO Director-General's opening remarks at the media briefing on COVID-19 – 11 March 2020 [Internet]. Geneva WHO; 2020
3. Thanh Le T, Andreadakis Z, Kumar A, Gomez Roman R, Tollefsen S, Saville M, et al. The COVID-19 vaccine development landscape. Nat Rev Drug Discov. 2020;19(5):305-6. doi: 10.1038/d41573-020-00073-5. PubMed PMID: 32273591.
4. Dzinamarira T, Nachipo B, Phiri B, Musuka G. COVID-19 Vaccine Roll-Out in South Africa and Zimbabwe: Urgent Need to Address Community Preparedness, Fears and Hesitancy. Vaccines. 2021;9(3). Epub 2021/04/04. doi: 10.3390/vaccines9030250. PubMed PMID: 33809002; PubMed Central PMCID: PMCPMC8000117.
5. World Health Organisation. WHO Coronavirus (COVID-19) Dashboard. https://covid19.who.int/table.
6. Bradshaw D, Dorrington R, Laubscher R, Groenewald P, Moultrie T. COVID-19 and all-cause mortality in South Africa – the hidden deaths in the first four waves. South African Journal of Science. 2022;118(5/6). doi: 10.17159/sajs.2022/13300.
7. Ackah BBB, Woo M, Stallwood L, Fazal ZA, Okpani A, Ukah UV, et al. COVID-19 vaccine hesitancy in Africa: a scoping review. Global Health Research and Policy. 2022;7(1):21. doi: 10.1186/s41256-022-00255-1.
8. Jassat W, Mudara C, Ozougwu L, Tempia S, Blumberg L, Davies M-A, et al. Difference in mortality among individuals admitted to hospital with COVID-19 during the first and second waves in South Africa: a cohort study. The Lancet Global Health. 2021;9(9):e1216-e25. doi: 10.1016/S2214-109X(21)00289-8.
9. Solanki G, Wilkinson T, Bansal S, Shiba J, Manda S, Doherty T. COVID-19 hospitalization and mortality and hospitalization-related utilization and expenditure: Analysis of a South African private health insured population. PLOS ONE. 2022;17(5):e0268025. doi: 10.1371/journal.pone.0268025.
10. Collie S, Nayager J, Bamford L, Bekker LG, Zylstra M, Gray G. Effectiveness and Durability of the BNT162b2 Vaccine against Omicron Sublineages in South Africa. The New England journal of medicine. 2022;387(14):1332-3. Epub 2022/09/15. doi: 10.1056/NEJMc2210093. PubMed PMID: 36103455; PubMed Central PMCID: PMCPMC9511610.
11. Collie S, Champion J, Moultrie H, Bekker LG, Gray G. Effectiveness of BNT162b2 Vaccine against Omicron Variant in South Africa. The New England journal of medicine. 2022;386(5):494-6. Epub 2021/12/30. doi: 10.1056/NEJMc2119270. PubMed PMID: 34965358; PubMed Central PMCID: PMCPMC8757569.
12. Bekker LG, Garrett N, Goga A, Fairall L, Reddy T, Yende-Zuma N, et al. Effectiveness of the Ad26.COV2.S vaccine in health-care workers in South Africa (the Sisonke study): results from a single-arm, open-label, phase 3B, implementation study. Lancet. 2022;399(10330):1141-53. Epub 2022/03/21. doi: 10.1016/s0140-6736(22)00007-1. PubMed PMID: 35305740; PubMed Central PMCID: PMCPMC8930006 ViiV Health Care, and Gilead. .
13. Gray G, Collie S, Goga A, Garrett N, Champion J, Seocharan I, et al. Effectiveness of Ad26.COV2.S and BNT162b2 Vaccines against Omicron Variant in South Africa. New England Journal of Medicine. 2022;386(23):2243-5. doi: 10.1056/NEJMc2202061. PubMed PMID: 35507482.
14. Kassanjee R, Davies M-A, Heekes A, Mahomed H, Hawkridge AJ, Morden E, et al. COVID-19 Vaccine Uptake and Effectiveness by Time since Vaccination in the Western Cape Province, South Africa:

An Observational Cohort Study during 2020–2022. Vaccines. 2024;12(6):628. PubMed PMID: doi:10.3390/vaccines12060628.

15. Vandenbroucke JP, von Elm E, Altman DG, Gøtzsche PC, Mulrow CD, Pocock SJ, et al. Strengthening the Reporting of Observational Studies in Epidemiology (STROBE): Explanation and Elaboration. PLOS Medicine. 2007;4(10):e297. doi: 10.1371/journal.pmed.0040297.

16. Centre for Respiratory Diseases and Meningitis. Proposed definition of COVID-19 wave in South Africa. Communicable Diseases Communiqué. November 2021, Vol. 20 (11). https://www.nicd.ac.za/wp-content/uploads/2021/11/Proposed-definition-of-COVID-19-wave-in-South-Africa.pdf.

17. National Department of Health.. Media Release: Cabinet approves changes to COVID-19 regulations. 30 December 2021. https://sacoronavirus.co.za/2021/12/30/media-release-cabinet-approves-changes-to-covid-19-regulations/.

18. Council for Medical Schemes. Annual Report 2021/22. https://www.medicalschemes.co.za/cms-annual-report-2021-22/.

19. Havers FP, Pham H, Taylor CA, Whitaker M, Patel K, Anglin O, et al. COVID-19-Associated Hospitalizations Among Vaccinated and Unvaccinated Adults 18 Years or Older in 13 US States, January 2021 to April 2022. JAMA Internal Medicine. 2022;182(10):1071-81. doi: 10.1001/jamainternmed.2022.4299.

20. Discovery Health. When are you fully vaccinated? September 2021. https://www.discovery.co.za/corporate/covid19-when-are-you-fully-vaccinated.

21. South African Medical Association. How to Code for COVID-19 and the necessity of legal disclaimers (Updated Article). Med-e-Mail. https://www.samedical.org/cmsuploader/viewArticle/1119.

22. Stats SA. PO141- Consumer Price Index (CPI). CPI History. 11 June 2024. https://www.statssa.gov.za/publications/P0141/CPIHistory.pdf?SCH=73795. .

23. Fathi M, Vakili K, Sayehmiri F, Mohamadkhani A, Hajiesmaeili M, Rezaei tavirani M, et al. The prognostic value of comorbidity for the severity of COVID-19: A systematic review and meta-analysis study. PLOS ONE. 2021;16:e0246190. doi: 10.1371/journal.pone.0246190.

24. Council for Medical Schemes. Guidelines for the Identification of Beneficiaries with Risk Factors in Accordance with the Entry and Verification Criteria. Version 10.1. Pretoria: CMS.

25. StataCorp. 2023. Stata Statistical Software: Release 16.1. College Station, TX: StataCorp LLC.

26. South Africa. Protection of Personal Information Act No. 4 of 2013. https://www.gov.za/documents/promotion-access-information-act.

27. Chua H, Feng S, Lewnard JA, Sullivan SG, Blyth CC, Lipsitch M, et al. The Use of Test-negative Controls to Monitor Vaccine Effectiveness: A Systematic Review of Methodology. Epidemiology (Cambridge, Mass). 2020;31(1):43-64. Epub 2019/10/15. doi: 10.1097/ede.0000000000001116. PubMed PMID: 31609860; PubMed Central PMCID: PMCPMC6888869.

28. Zheng C, Shao W, Chen X, Zhang B, Wang G, Zhang W. Real-world effectiveness of COVID-19 vaccines: a literature review and meta-analysis. International journal of infectious diseases : IJID : official publication of the International Society for Infectious Diseases. 2022;114:252-60. Epub 2021/11/21. doi: 10.1016/j.ijid.2021.11.009. PubMed PMID: 34800687; PubMed Central PMCID: PMCPMC8595975.

29. Liu Q, Qin C, Liu M, Liu J. Effectiveness and safety of SARS-CoV-2 vaccine in real-world studies: a systematic review and meta-analysis. Infectious diseases of poverty. 2021;10(1):132. Epub 2021/11/16. doi: 10.1186/s40249-021-00915-3. PubMed PMID: 34776011; PubMed Central PMCID: PMCPMC8590867.

30. Mohammed I, Nauman A, Paul P, Ganesan S, Chen KH, Jalil SMS, et al. The efficacy and effectiveness of the COVID-19 vaccines in reducing infection, severity, hospitalization, and mortality: a systematic review. Hum Vaccin Immunother. 2022;18(1):2027160. Epub 20220203. doi: 10.1080/21645515.2022.2027160. PubMed PMID: 35113777; PubMed Central PMCID: PMCPMC8862168.

31. Whittaker R, Bråthen Kristofferson A, Valcarcel Salamanca B, Seppälä E, Golestani K, Kvåle R, et al. Length of hospital stay and risk of intensive care admission and in-hospital death among COVID-19 patients in Norway: a register-based cohort study comparing patients fully vaccinated with an mRNA vaccine to unvaccinated patients. Clinical microbiology and infection : the official publication of the European Society of Clinical Microbiology and Infectious Diseases. 2022;28(6):871-8. Epub 2022/02/28. doi: 10.1016/j.cmi.2022.01.033. PubMed PMID: 35219807; PubMed Central PMCID: PMCPMC8872711.

32. Fang X, Tao G, Zhou H, Zhou Y. Vaccines reduced hospital length of stay and fraction of inspired oxygen of COVID-19 patients: A retrospective cohort study. Preventive medicine reports. 2024;39:102632. Epub 2024/02/13. doi: 10.1016/j.pmedr.2024.102632. PubMed PMID: 38348219; PubMed Central PMCID: PMCPMC10859302.

33. Somani ST, Firestone RL, Donnelley MA, Sanchez L, Hatfield C, Fine J, et al. Impact of Vaccination on Cost and Course of Hospitalization Associated with COVID-19 Infection. Antimicrobial stewardship & healthcare epidemiology : ASHE. 2023;3(1):e19. Epub 2023/01/31. doi: 10.1017/ash.2022.364. PubMed PMID: 36714292; PubMed Central PMCID: PMCPMC9879923.

34. Chirico F, Teixeira da Silva JA, Tsigaris P, Sharun K. Safety & effectiveness of COVID-19 vaccines: A narrative review. The Indian journal of medical research. 2022;155(1):91-104. Epub 2022/07/22. doi: 10.4103/ijmr.IJMR_474_21. PubMed PMID: 35859436; PubMed Central PMCID: PMCPMC9552389.

35. Beatty AL, Peyser ND, Butcher XE, Cocohoba JM, Lin F, Olgin JE, et al. Analysis of COVID-19 Vaccine Type and Adverse Effects Following Vaccination. JAMA network open. 2021;4(12):e2140364. Epub 2021/12/23. doi: 10.1001/jamanetworkopen.2021.40364. PubMed PMID: 34935921; PubMed Central PMCID: PMCPMC8696570

Supplementary Tables.

Table S1. Comparison of study population versus rest of the insured in South Africa

Table S2. Hospitalisation risk dataset

Table S3. Hospital utilization and expenditure dataset

Figure Click here to access/download;Figure;Figure 1 approach to extraction Powerpoint 14_Oct_2024.pptx

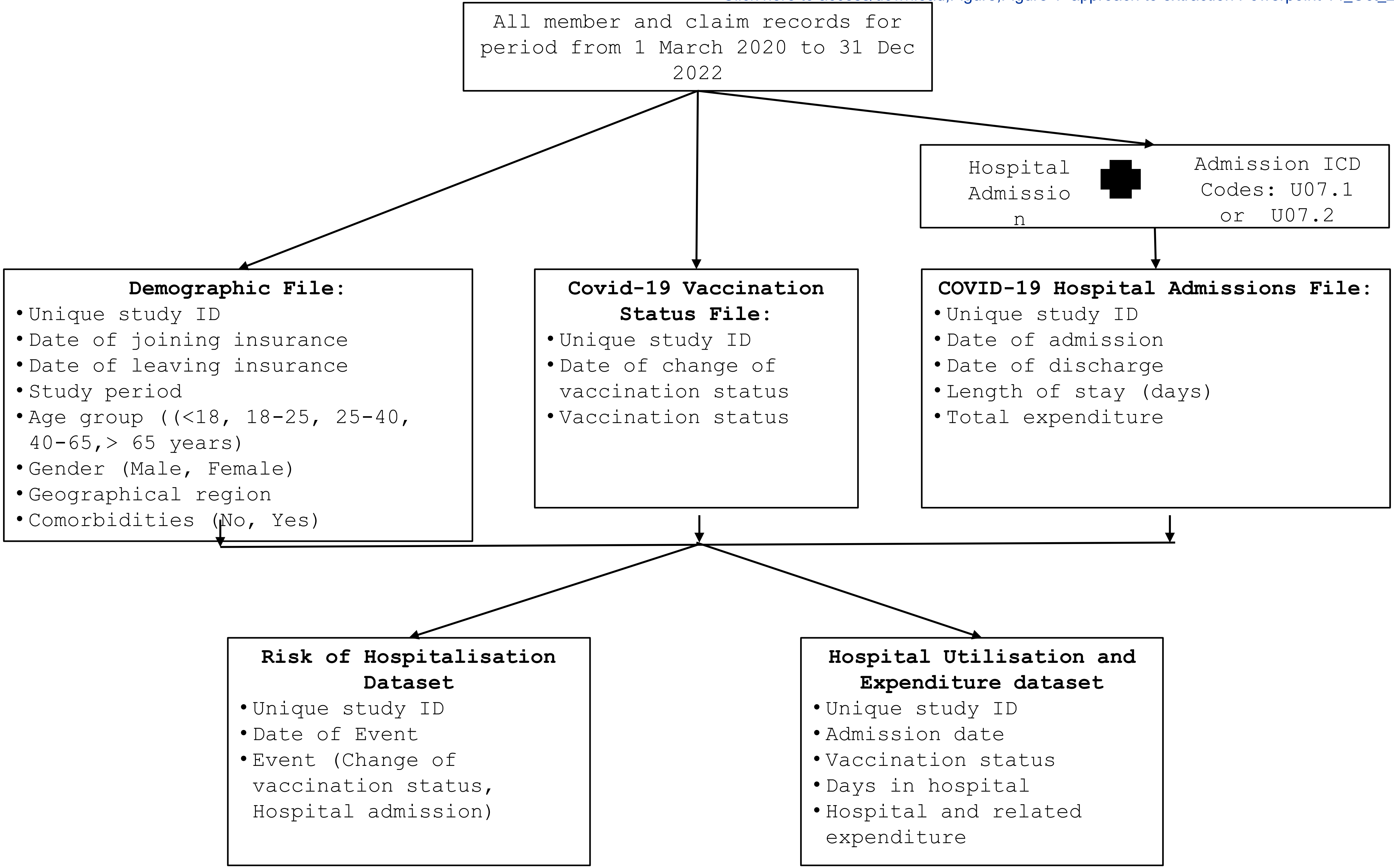

Click here to access/download;Figure;Figure 1 approach to extraction Powerpoint 14_Oct_2024.pdf

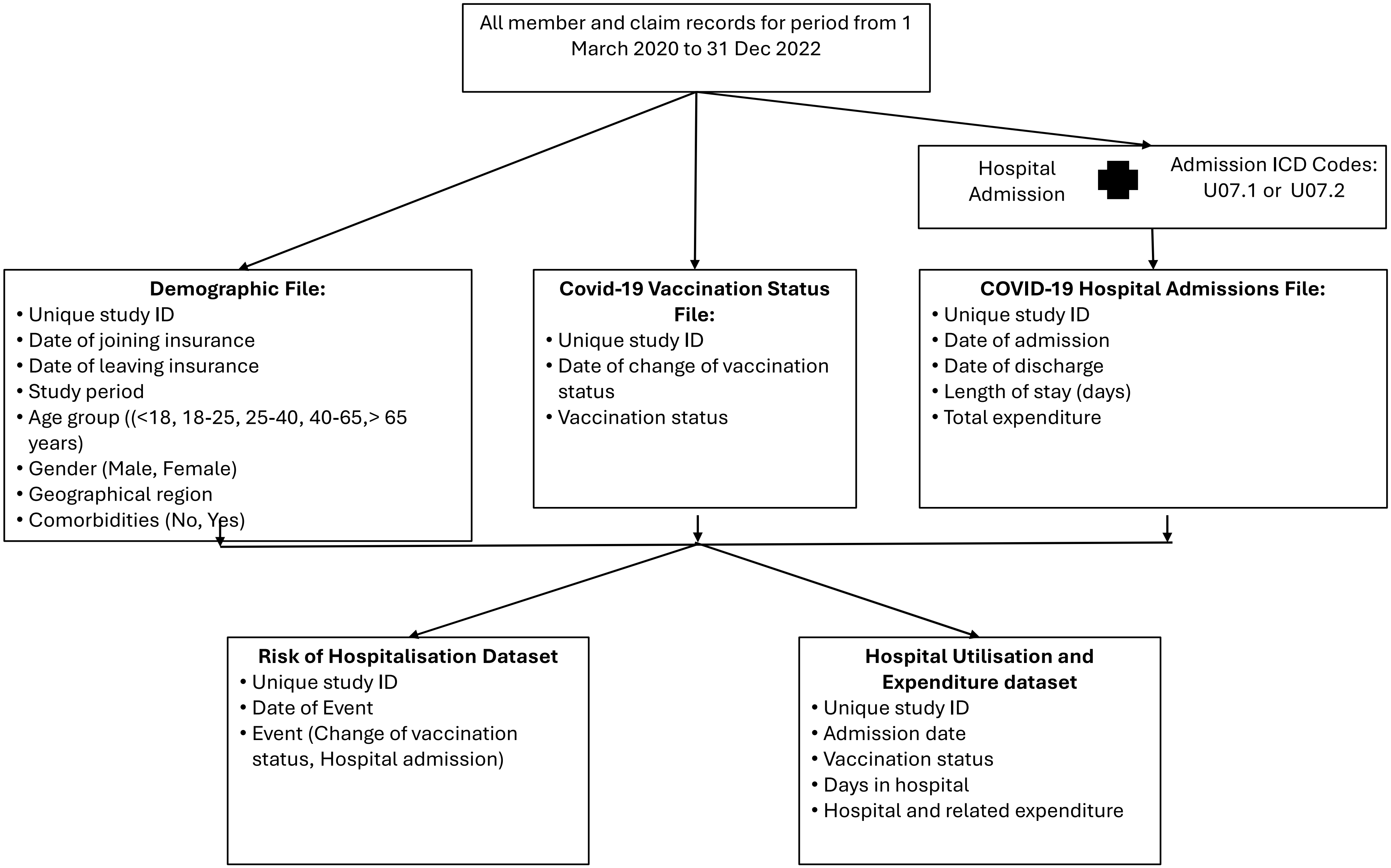

Tables in excel

Click here to access/download
Supporting Information
tables Impact of COVID vac Dec 27 2024.xlsx

Table S1

Click here to access/download

Supporting Information

S1 Comp vs rest SA insured population dec 27 dec 2024.xlsx

Click here to access/download
Supporting Information - Compressed/ZIP File Archive
S2 RiskHospitilisation_dataset.zip

Click here to access/download
Supporting Information - Compressed/ZIP File Archive
S3 HospUtilExpendData.zip

Impact of COVID-19 vaccination on ~~COVID-19~~ ~~hospitalisation~~hospitalization, hospital ~~related utilisation~~ utilization and expenditure for COVID-19: a~~A~~ retrospective cohort analysis of a South African private health insured population.

Geetesh Solanki[1, 2, 3], Susan Cleary[2,] Francesca Little[4],

Author affiliations

[1] Health Systems Research Unit, South African Medical Research Council

[2] Health Economics Unit, School of Public Health, University of Cape Town, Cape Town, South Africa

[3] NMG Consultants and Actuaries, Cape Town, South Africa

[4] Department of Statistical Sciences, University of Cape Town

Corresponding author: Geetesh Solanki

Email: geetesh.solanki@mrc.ac.za

South African Medical Research Council, Francie van Zijl Drive, Parow Valley | Cape Town | Western Cape, South Africa.

Abstract

Evidence on the impact of COVID-19 vaccination on COVID-19 hospitalization, utilisation and expenditures in the African context, in privately insured populations and in real world contexts is limited. This study quantifies the impact of COVID-19 vaccination on on hospitalization for COVID-19 infection these outcomes in a South African private health health insuredance population cohort. over the period of the pandemic using real world data. This is a retrospective cohort study is, based on an the epidemiologic and economic analysis of demographic and claims records of for 550,332 individuals belonging to two health insurance funds- between 1 March 2020 and 31 December 2022. In addition to the variable of interest, COVID-19 vaccination status (not vaccinated, partly vaccinated, fully vaccinated), gender, age, chronic conditions, and province were incorporated as predictor variables. A Cox Proportional Hazards model was used to estimate the impact of vaccination (not vaccinatednon-vaccinated, partly vaccinated, fully vaccinated) on COVID-19 hospitalisationhospitalization risk; and zero-inflated negative binomial models were used to estimate the impact of vaccination on hospital utilisation utilization and hospital expenditure for COVID-19 infection, with adjustments for age, . The COVID-19 related hospitalization rate (events per 1000 individuals per year) was 10.29, 4.47 and 1.97 for the non-vaccinated, 4.47 for the partly vaccinated and 1.97 for the fully vaccinated respectively. After adjustmentsex, comorbidities and province of residence. In comparison to the non-vaccinated, , the COVID-19 hospitalisationhospitalization rate for COVID-19 was 94.51% (aHR 0.06, 95%CI 0.06, 0.07) lower for the partly vaccinated and 93.49% -(aHR 0.07, 95%CI 0.06, 0.07) lower lower for the partly and fully vaccinated respectively; hospital utilization was 17.70% (95% CI 24.78%, 9.95%) and 20.04% (95% CI 28.26%, 10.88%) lower; the in comparison with the non-vaccinated. The hospital utilization rate (days per 1000 individuals per year) was 93.82, 44.11 and 20.52 for the non-vaccinated, 44.11 for the partly vaccinated and 20.52 for the fully vaccinated respectively. After adjustment, Hhospital utilisation was

17.70% (95% CI -24.78%, -9.95%) and 20.04% (95% CI -28.26%, -10.88%) lower for the partly and fully vaccinated compared with the non-vaccinated. The relative risk of zero hospital days was 4.34 (95% CI 4.02, 4.68) for the partly vaccinated increasing and to 18.55 (95% CI 17.12, 20.11) higher; for hthe fully vaccinated compared with the non-vaccinated. The per annum COVID-19 related hospital expenditure for non-vaccinated individuals was USD18, for the partly vaccinated USD6 and for the fully vaccinated USD2. After adjustment, Hhospital expenditure was 32.83% (95% CI -41.06%, -23.44%) and 55.29% (95% CI -61.13%, -48.57%) lower for the partly and fully vaccinated; and the relative risk of zero hospital expenditure was 4.38 (95% CI 4.06, 4.73) and 18.61 (95% CI 17.18, 20.16) higher for the partly and fully vaccinated respectively.

Taken together, when compared with the non-vaccinated. Compared with the non-vaccinated, the relative risk of zero hospital expenditure was 4.38 (95% CI 4.06, 4.73) and 18.61 (95% CI 17.18, 20.16) for partly and fully vaccinated respectively. The findings of this study findings indicateconfirm suggests that all measures of hospitalization for COVID-19 COVID-19 vaccination substantially reduced hospitalisation risk, hospital utilisation and expenditure for COVID-19 infection waswere significantly lower in the partly or fully vaccinated in comparison to the non-vaccinated substantially lower amongst those who had vaccinated. The use of real-world data and an aggregated level of analysis resulted in the study having several limitations. While the overall results may not be generalizable to other populations, the findings add to the evidence based on the impact of COVID-19 vaccination during the period of the pandemicThe study has several limitations. Only services and vaccines for which claims were submitted were analysed. The macro aggregated level at which the analysis was carried out comes at the expense of providing a more granular view of the impact of the virus and vaccine variants. Given the differences in insured and non-insured populations, caution should be exercised in generalizing the findings of this study. Despite this, the results of this study help fill the knowledge gap on the overall "real-world"

impact of COVID-19 vaccination across the entire pandemic period, particularly in insured populations and those in Sub-Saharan Africa.

Introduction

The World Health Organization (WHO) declared COVID-19 ~~was declared~~ a Public Health Emergency of International Concern ~~on 30 January 2020,~~ and a pandemic on 30 January and 11 March 2020 respectively~~by the World Health Organization (WHO)~~ [1, 2], triggering an international response to develop a ~~preventive~~ COVID-19 vaccine with four vaccine candidates entering human evaluation by March 2020[3]. South Africa commenced its national COVID-19 vaccination program on 17 February 2021[4]. By 31 December 2023, based on the ~~World Health Organisation (~~WHO~~)~~ COVID-19 dashboard, South Africa had a total of 4.1 million confirmed COVID-19 ~~confirmed~~ cases~~;~~ and 70 vaccination doses ~~had been administered~~ per 100 population with~~,~~ 41 ~~persons~~ per 100 population ~~had completed~~completing the primary series ~~vaccination,~~ and 7 ~~persons~~ per 100 population ~~had completed the primary series and~~ receiving at least one additional dose or booster ~~vaccination~~ [5]. While it is likely that COVID-19 deaths were underreported, between~~Studies indicate that over the period from~~ December 2019 ~~to~~ and January 2022,~~-~~ there were 295,135 excess natural deaths in South Africa with evidence suggesting that ~~(~~most ~~likely~~ were due to COVID-19~~)~~ [6].

While there is now ~~a~~ substantial global evidence on the impact of COVID-19 vaccines, research in African regions remains limited. Societies in these regions ~~are highly segmented, with~~have relatively poor access ~~significant disparities in access~~ to health care services, ~~and during the COVID-19 pandemic,~~ varying levels of vaccine awareness~~,~~ and ~~differences~~ sub-optimal ~~in~~ vaccine coverage with ~~.v V~~accine hesitancy ~~also emerged~~emerging as an ~~major~~ obstacle to ~~implementing~~ the uptake of COVID-19 vaccines ~~strategies in several regions~~during the pandemic ~~in Africa~~ [7]~~. The overall impact of vaccination across the different social strata in these countries can therefore be expected to be different and merits further exploration~~. In ~~The available~~ South Africa,~~n~~ evidence confirms that the risk of COVID-19 related morbidity and mortality ~~risk~~ varied ~~amongst other factors, from wave to wave~~across waves and COVID-19 variants, by

age, ~~gender~~sex, level of comorbidity, , and whether dependent on ~~the~~ public or private health care[8, 9]. ~~Following on the introduction of the COVID-19 vaccine, a small number of s~~Studies have also confirmed the efficacy of specific COVID-19 vaccines against specific COVID-19 variants [10-13] and the effectiveness of the vaccine in "real world" situations [12, 14]. However, ~~there remain~~ gaps in knowledge remain ~~in the knowledge base with respect to~~ regarding the overall "real world" impact of COVID-19 vaccination as a strategy to manage COVID-19 related morbidity~~, mortality~~ and ~~health related~~health care expenditure during the pandemic.

Using Real World Data (RWD), t~~T~~he aim of this paper is to ~~assess and~~ quantify the impact of COVID-19 ~~vaccination~~ vaccination on ~~COVID-19 hospitalisation~~hospitalization, hospital ~~related utilisation~~ utilization and hospital expenditure for COVID-19 infection, ~~for COVID-19~~based on data from ~~in~~ a South African private health insured population cohort over the entire period of the COVID-19 pandemic~~ in South Africa using Real World Data (RWD)~~. ~~The results from this study contribute to addressing the gap in knowledge on the aggregate "real life" impact of COVID-19 vaccination in an insured population cohort over the entire period of the pandemic in South Africa. The study will also generate information to assess the effectiveness, cost and cost effectiveness of COVID-19 vaccination in preventing COVID-19 related morbidity.~~

Methods

Study design, population and period ~~and population~~

~~The~~ Strengthening and Reporting of Observational Studies in Epidemiology (STROBE) guidelines were used to conduct and report on this study[15]. The study ~~was designed as~~is a retrospective cohort analysis ~~to evaluate~~evaluating the impact of COVID-19 vaccination on hospitalization, ~~healthcare~~ utilization~~,~~ and expenditure for COVID-19 infection~~ for COVID-19. The study is This is a retrospective~~

~~cohort study, based on an epidemiologic and economic analysis of the demographic and medical claims records of individuals belonging to two large health insurance funds~~. The study population included 550,332 individuals who were enrolled in one of two large health insurance funds during the study period of 1 March 2020 to 31 December 2022, corresponding to the first reported case of COVID-19 in South Africa on 5 March 2020 [16] and the end of the ~~of the~~ fourth wave and removal or COVID-19 restrictions on 30 December 2022[17]. For individuals who were members of these insurance funds for the entire pandemic, their individual study period corresponded to the overall study period; while the actual date of entry or exit was used for those who joined or left one of the funds during the pandemic. This population represents approximately 5% of the South African private health insurance population and is comparable to the broader insured population in terms of family size, health insurance contributions and health care expenditure (Table S1)[18].

Exposures and outcomes of interest

Formatted: Heading 2

The exposure of interest was defined as non-vaccinated, partly vaccinated or fully vaccinated. The South African government authorized the Johnson and Johnson (J&J) and the Pfizer BioNtech vaccines. In line with the literature [19] as well as the approach used in South Africa [20], individuals were initially categorized as non-vaccinated, becoming partly vaccinated the day after their first vaccine dose (J&J or Pfizer BioNtech), and fully vaccinated 28 days after their first J&J or 14 days after the second dose of Pfizer BioNtech. Individuals receiving booster vaccinations remained in the fully vaccinated category.

Outcomes were defined as the risk of COVID-19 ~~hospitalisation~~hospitalization (individual having a hospital admission or not), the extent of COVID-19 hospital ~~utilisation~~ utilization (number of days in hospital) and the extent of COVID-19 hospital expenditure. COVID-19 hospital admissions were identified using ICD-10 code (10th revision of the International Statistical Classification of Diseases and Related Health Problems) U07.1 or U07.2[21]. For these admissions, hospital ~~utilisation~~ utilization was defined using data on the date of admission and date of discharge or death in hospital. Total hospital and related expenditures took an insurer perspective and reflected the amounts paid by the health insurance funds to cover the claims submitted by health care providers for services rendered to the hospitalized individuals. These direct costs included health care providers, hospital bed days, as well as any pharmaceuticals, medical or surgical consumables dispensed. All expenditures in South African Rands (ZAR) were converted to United States Dollar ($) values using the December $/ZAR exchange rate for the given year and then inflated/deflated to be expressed in 2022 values using the South African Consumer Price Index[22].

In addition to the above exposures and outcomes, the analysis incorporated sex (male, female), age (less than 18, between 18 and 25, between 25 and 40, between 40 and 65 and greater than 65), COVID-19 comorbidities (yes, no) and province of residence (Eastern Cape, Free State, Gauteng, KwaZulu-Natal, Limpopo, Mpumalanga, North West, Northern Cape, Western Cape) as predictor variables. Comorbidities ~~included~~ for COVID 19 considered in the analysis included cancers, chronic renal disease, congestive cardiac failure, chronic obstructive pulmonary disease, diabetes mellitus, HIV, hypercholesterolaemia, hypertension, hypothyroidism, ischaemic heart disease, pregnancy and tuberculosis, given that these have been identified as being important comorbidities for COVID-19 [23]. The individuals with these COVID-19 comorbidities were identified using Council for Medical Schemes (CMS) Guidelines for identifying these conditions using claims records [24].

~~The study period extended from a start date of 1 March 2020 to an end date of 31 December 2022. This study period was chosen to cover the four waves of the COVID-19 pandemic in South Africa with the first wave starting on 5 March 2020[16] (first reported case of COVID-19 in South Africa) and the fourth wave "ending" with the lifting of restrictions related to the wave on 30 December 2022[17].~~

~~The study population consisted of all individuals who belonged to either of the two health insurance funds at any time over the study period. The study sample consisted of 550,332 individuals representing around 5% of South Africa's privately insured population. The average family size, average contributions and health care expenditure of the study population were compared with those of the overall health insured population and found to be broadly comparable, thus ensuring that the findings of this study are generalisable to the broader South African population with private health insurance (Table S1)[18].~~

Data Collection

Data for the study were extracted from the data warehouse of NMG Consultants and Actuaries (NMG), an independent consulting firm providing consulting and actuarial services to the two health insurance funds. Electronic demographic and claims records for the insured individuals, stored on a Microsoft SQL Server in the NMG data warehouse, were extracted in comma-separated values (CSV) format and then imported into STATA/SE 16.1[25] –for data cleaning, descriptive analyses, and statistical modeling.

For each insured individual, the data contains the following variables: a unique individual identifier; date of birth; sex; and provinceAlthough the overall study period extended from 1 March 2020 to 31 December 2022 (34 months or 2.83 years), there are some individuals who joined the health insurance funds after 1 March 2020 and some who left before 31 December 2022. For individuals who were members of the insurance funds for the entire period, the study start date and end date were recorded as their start and end dates. For those who joined the insurance funds after 1 March 2020 or left before 31 December 2022 the actual entry and exit dates were recorded as the study start or end dates.. For each claim submitted to the administrator for reimbursement of services rendered or items dispensed to an insured individual while hospitalised, the data contains the following variables: a unique individual identifier; dates of commencement and completion of the service; a code and description for each service rendered/item dispensed; an ICD-10 (10th revision of the International Statistical Classification of Diseases and Related Health Problems) code for the diagnosis of the condition being treated; a Current Procedural Terminology (CPT) code for the procedure carried out; a National Pharmaceutical Product Index (NAPPI) code for any surgical, medical or consumable item dispensed; the amount claimed (amount charged by provider) and the expenditure incurred (amount paid by insurer).

The approach used to extract and classify the data is schematically summarized in Figure 1. From all the data for the period, a 3-step approach was used to extract and compile the data into various datasets.

Fig 1. Approach to data extraction

~~For the first step~~In the first step, three data files were created. The first “demographic” file consisted of a record per individual who belonged to either of the two health insurance funds at any time over the study period (unique study ID, date of joining and exiting the fund, age, sex, province of residence, and comorbidities). ~~Each record captured the demographic information for the individual (unique study ID, date of joining and exiting the fund, study period, age group, sex, geographical region, and co-morbidities). Although the overall study period extended from 1 March 2020 to 31 December 2022 (34 months or 2.83 years), there are some individuals who joined the health insurance funds after 1 March 2020 and some who left before 31 December 2022. For individuals who were members of the insurance funds for the entire period, the study start date and end date were recorded as their start and end dates. For those who joined the insurance funds after 1 March 2020 or left before 31 December 2022 the actual entry and exit dates were recorded as the study start or end dates. For the comorbidity variable, t~~The 26 chronic disease list conditions listed by the South African Council for Medical

Schemes(CMS) as part of the Prescribed Minimum Benefits were considered for defining the Covid-19 comorbidity status[26] variable. Of the 26 conditions, Cancer, Chronic Renal Disease, Congestive Cardiac Failure, Chronic Obstructive Pulmonary Disease, The Diabetes Mellitus, HIV, Hypercholesterolaemia, Hypertension, Hypothyroidism, Ischaemic Heart Disease, Pregnancy, Tuberculosis have been identified as being important comorbidities for COVID-19. The individuals with these Covid-19 comorbidities were identified using the CMS Guidelines for identifying members with medical conditions using claims records [24].

The second "COVID-19 vaccination status" data file consisted of a record for every change in the vaccination status of an individualvaccination event in an individual (unique study ID, date of change of vaccination status, vaccination status). The South African government authorized the Pfizer BioNtech and Johnson and Johnson (J&J) vaccines. For the purposes of this study, in line with approaches used in studies elsewhere[19] and the categorization used in South Africa[20], individuals were categorized as fully vaccinated 28 days after receiving the J&J vaccine or 14 days after receiving the second dose of Pfizer BioNtech. Individuals receiving further booster vaccinations remained in the fully vaccinated category. Individuals were categorized as partiallypartly vaccinated the day after the first vaccine dose (J&J or Pfizer BioNtech) until meeting the criteria for complete vaccination. Individuals were recorded as non-vaccinated if they had received no vaccinations. Individuals received their vaccinations at various points in time over the study period and their vaccination status therefore changed over time. For those who were hospitalized, the vaccination status at the time of admission for each hospital event was recorded. For those who were not hospitalized, the vaccination status at the end point of the study was recorded.

The "COVID-19 hospital admission" data file consisted of a record for each COVID-19 related hospital admission for COVID-19 infection (unique study ID, date of admission, date of discharge, length of stay and total hospital and related expenditure). These admissions were identified using admission ICD codes

U07.1 or U07.2[21]. Individuals with multiple COVID-19 admissions had separate records for each admission. The total hospital and related expenditures were from an insurer perspective and reflected the amounts claimed paid by the health insurance funds to cover the claims submitted by health care providers from the health insurance funds for services rendered to the hospitalized individuals. For those who were hospitalized, vaccination status (not, partly and fully vaccinated) on the date of admission was assumed.

For the second step, two separate datasetsdata sets were created using information from the three files described above. Each dataset included at least one record for the 550,332 individuals included in the study population. The first dataset enabled the analysis of the risk of COVID-19 hospitalization events. It included The first dataset, created to analyze the risk of hospitalisation, consisted of "time stamped" records for every hospitalization for COVID-19 hospitalisation infection or change of COVID-19 vaccination status event, together with records for all individuals who had none of these events. Multiple records were created for individuals with more than one COVID-19 hospitalisationhospitalization or COVID-19 vaccination event. The time stamp was created by determining the number of days from study entry start date (1 March 2020) for the individual to the date of the event. The second dataset enabled the analysis of Each record included demographic information of the individual, hospitalisation and vaccination status indicators and the time stamp for the event.

The second dataset, created to analyse hospital utilisation utilization and expenditure for COVID-19 infection. Data included the number of days spent in hospital and expenditure per hospitalization (zero if no hospital event)s, consisted of a record for every individual who had no hospital event over the period and a record for every hospital event for individuals hospitalised over the period. Each record included demographic information of the individual, a hospitalisation indicator, a vaccination status indicator, and number of hospital days and expenditure per hospital event (zero if no hospital event).

For each hospital event the vaccination status indicator reflected the vaccination status as at the time of hospitalisation. For those who had no hospital events, the vaccination status indicator reflected the vaccination status of the individual at the end of the study period.

Exposure and outcomes of interest

The impact of COVID-19 vaccination was assessed on three COVID-19 related outcomes – the risk of hospitalisation (individual having a hospital event or not), extent of hospital utilisation (number of days in hospital) and extent of hospital expenditure (total hospital and related expenditure in 2022 USD prices).

, partly vaccinated, fully vaccinated), the analysis incorporated sex (male, female), age (less than 18, between 18 and 25, between 25 and 40, between 40 and 65 and greater than 65), COVID-19 comorbidities (yes, no) and province (Eastern Cape, Free State, Gauteng, KwaZulu-Natal, Limpopo, Mpumalanga, North West, Northern Cape, Western Cape) as predictor variables.

Statistical analysis

~~The impact of COVID-19 vaccination was assessed on three COVID-19 related outcomes – the risk of hospitalisation (individual having a hospital event or not), extent of hospital utilisation (number of days in hospital) and extent of hospital expenditure (total hospital and related expenditure in 2022 USD prices).~~

~~In addition to the variable of interest, COVID-19 vaccination status (not vaccinated, partly vaccinated, fully vaccinated), the analysis incorporated sex (male, female), age (less than 18, between 18 and 25, between 25 and 40, between 40 and 65 and greater than 65), chronic condition (yes, no) and province (Eastern Cape, Free State, Gauteng, KwaZulu-Natal, Limpopo, Mpumalanga, North West, Northern Cape, Western Cape) as predictor variables.~~

For the analysis of the impact of vaccination on risk of ~~hospitalisation~~hospitalization, summary statistics reported include frequencies and percentages for the number of individuals, and ~~hospitalisation~~hospitalization frequencies and rates (number of hospital events per 1000 individuals per annum). For the multivariate analysis, survival analysis was used to model the impact of vaccination on the risk of ~~hospitalisation~~hospitalization for COVID-19 ~~infeciton~~infection to account for censoring due to no relevant ~~hospitalisation~~hospitalization during the observation period. A Cox Proportional Hazards model was used to estimate the relative hazard of hospitalization for COVID-19 infection in partly or fully vaccinated versus non-vaccinated ~~subjects~~individuals. To factor in the censoring of the follow-period in the analysis, the Cox PH model was used for the primary analysis of the association between vaccination status and the risk of hospitalization, The model allows for those subjects who were never hospitalized during the follow-up period to be accommodated as censored observations. ~~.~~

Time-varying vaccination status was incorporated through repeated measures per individual relating to the periods with different vaccination statuses. The large dataset did not allow for estimation of shared frailty models. The repeated observations per individual (individuals with multiple vaccination events and multiple hospital events) was taken into consideration by incorporating the data clustering option by individual in the modelling and hence the ~~estimation~~ use of robust ~~standard errors~~ estimators, allowing errors to be correlated within subjects but independent between subjects. The proportional hazards assumption was assessed through Kaplan-Meier curves and found to be justifiable. The association between vaccination status and risk of hospitalization are reported as u~~U~~nadjusted Hazard Ratios (UHR) and a~~A~~djusted Hazard Ratios (AHR) with 95% confidence intervals. ~~are presented.~~

For the analysis of the impact of COVID-19 vaccination on hospital ~~utilisation~~utilization for COVID-19, the summary statistics included the total number of days in hospital, number of days per ~~hospitalisation~~hospitalization event and the number of hospital days per 1000 individuals per annum. ~~For the expenditure analysis, all the expenditures in South African Rands (ZAR) were converted to US Dollar (USD$) values using the December USD$/ZAR exchange rate for the given year and then inflated/deflated to be expressed in 2022 values using the South African Consumer Price Index~~[22].~~.~~ For the expenditure analysis, ~~T~~the summary statistics include the total expenditure in millions (~~USD~~$), expenditure per ~~hospitalisation~~hospitalization event and the expenditure per individual per annum.

For the multivariate analysis of the impact of vaccination on ~~utilisation~~ utilization and expenditure, zero-inflated negative binomial models were used as the exploratory analyses indicated a highly skewed distribution not only because of the zeros, but also a skewed distribution on the non-zero values. The zero-inflated negative binomial model produces two sets of outputs: the count component and the zero-inflated component. The count component examines the effects of predictor variables on the expected

non-zero count of the outcome variable, while the zero-inflated component explores factors influencing the probability of the presence of excess zeros. The association between vaccination and utilization and expenditure as estimated by these models are presented as percentage increase or decrease in the number of average days/ expenditures depending on whether the coefficient from the count component of the model (e.g., beta1) is positive or negative using the formula, (exp(beta1)-1) x100, and (ii) as the risk ratio for zero hospital days in the vaccinated versus the non-vaccinated, or relative to the reference group for other predictor variables, using the formula exp(beta) where beta refers to the coefficients of the zero-inflated component of the model and their 95% confidence intervals. Both unadjusted estimates from univariate models and adjusted estimates from multivariable models are presented. The lack of independence due to repeated observations per individual (individuals with multiple hospital events) ~~would ideally have been dealt with by the inclusion of random effects in the model. However, as STATA does not allow for the incorporation of random effects in zero inflated models,~~ was dealt with through the estimation of robust standard errors allowing correlated errors within subject and uncorrelated errors between subjects. ~~the data clustering option by subject was used to derive robust standard errors.~~

~~The zero-inflated negative binomial model produces two sets of outputs: the count component and the zero-inflated component. The count component examines the effects of predictor variables on the expected non-zero count of the outcome variable, while the zero-inflated component explores factors influencing the probability of the presence of excess zeros.~~ ~~The unadjusted and adjusted estimated coefficients (e.g. beta1) from the count component of the model output were expressed as percentage increase or decrease in the number of average days/ expenditures depending on whether the coefficient is positive or negative using the formula, (exp(beta1)-1) x100. The unadjusted and adjusted estimated coefficients from the zero inflated component were expressed as the risk ratio for zero hospital days compared with the reference group using the formula exp(beta).~~ No specific cut-off was used to

determine statistical significance, and no p-values are reported. The focus is on effect sizes and their 95% confidence intervals. STATA/SE 16.1 was used for all the analyses.

Ethical considerations

The data for the study were accessed in terms of and under the conditions set out in the agreement between NMG and the client insurance funds and between NMG and the researcher. The terms set out in these agreements ensured that the access ~~to~~ and use of the data is in full compliance with the requirements of the Protection of Personal Information (PoPI) Act in South Africa[26]. Ethics approval for the use of the database to carry out this study was granted by the Human Research Ethics Committee of the Faculty of Health Sciences, University of Cape Town (~~project registration~~reference number ~~HREC Ref:~~ 606/2023).

Results

Characteristics of the study population

The study cohort ~~total dataset comprised the information on~~~~was made up of~~included 550,332 individuals who belonged to the two health insurance funds at any time between 1 March 2020 and 31 December 2022 (1029 days). The average duration of membership for these individuals was 864 days, with 70% of the individuals being members of the funds for the full study period. At the end of the study period, 62.1% were ~~not vaccinated~~non vaccinated~~non-vaccinated~~, 12.2% were partly vaccinated, and 25.7% were fully vaccinated. Of all the individuals, 53.6% were female, 34.4% were less than 18 years old, 23.3% had one or more ~~chronic conditio~~comorbidity~~n(s)~~, and the ~~largest~~ highest proportion (45.4%) of individuals ~~was from~~ resided in the Gauteng province (Table 1).

Table 1. Characteristics of study population

Variable	Number of individuals	
	N	%
**Vaccination Status***		
Non vaccinated	341 972	62.1%
Partly vaccinated	66 885	12.2%
Fully vaccinated	141 475	25.7%
**Sex**		
Female	295 116	53.6%
Male	255 216	46.4%
**Age**		
Less than 18	189 405	34.4%
Between 18-25	40 190	7.3%
Between 25-40	145 004	26.3%
Between 40-65	140 255	25.5%
Greater than 65	35 478	6.4%
**COVID-19 Comorbidities**		
No	422 116	76.7%
Yes	128 216	23.3%
**Province**		
Eastern Cape	40 694	7.4%
Free State	22 347	4.1%
Gauteng	249 650	45.4%
KwaZulu-Natal	85 572	15.5%
Limpopo	19 410	3.5%
Mpumalanga	18 514	3.4%
North West	19 064	3.5%
Northern Cape	10 002	1.8%
Western Cape	85 079	15.5%
**Total**	550 332	100.0%

Vaccination Status*: Status as at end of study.

Non-Variable	Number of individuals	
	N	%
**Vaccination Status***		
Not vaccinatedNon vaccinated	341,972	62.1%
Partly vaccinated	66,885	12.2%
Fully vaccinated	141,475	25.7%

Formatted: Font: Bold

Formatted Table

**Sex**	-	-
Female	295,116	53.6%
Male	255,216	46.4%
**Age**	-	-
Less than 18	189,405	34.4%
Between 18-25	40,190	7.3%
Between 25-40	145,004	26.3%
Between 40-65	140,255	25.5%
Greater than 65	35,478	6.4%
**Chronic Condition?**	-	-
No	422,116	76.7%
Yes	128,216	23.3%
**Province**	-	-
Eastern Cape	40,694	7.4%
Free State	22,347	4.1%
Gauteng	249,650	45.4%
KwaZulu-Natal	85,572	15.5%
Limpopo	19,410	3.5%
Mpumalanga	18,514	3.4%
North-West	19,064	3.5%
Northern Cape	10,002	1.8%
Western Cape	85,079	15.5%
**Total**	550,332	100.0%

Vaccination Status*: Status as at end of study.

~~Hospitalisation~~Hospitalization Risk for COVID-19 infection

Formatted: Line spacing: Double

As shown in Table 2, t~~T~~he ~~overall~~ hospit~~a~~ilisation rate ~~for COVID-19 infection~~ ~~related hospitalisation rate~~ for COVID-19 infection (events per 1000 individuals per year) ~~for the study population~~ was 7.45 (95%CI 7.31, 7.58) in the study population; ~~(Table 2). The hospitalisation rate was~~ 10.29 (95%CI 10.09, 10.49) for the non-vaccinated;~~,~~ 4.47 (95%CI 4.17, 4.78) ~~-~~for the partly vaccinated and 1.97 (95%CI 1.84, 2.11) for the fully vaccinated. Across the other predictor variables, males (7.56, 95%CI 7.36, 7.76), individuals aged 65 and above (25.62, 95%CI 24.63, 26.61), those with 1 or more ~~chronic conditions~~comorbidity (20.12, 95%CI 19.66, 20.58) and those from the Western Cape (9.02, 95%CI 8.64, 9.40) had ~~the highest~~higher ~~hospitalisation~~hospitalization rates.

In multivariate analysis, after adjustment for all other factors, the risk of COVID-19 ~~hospitalisation~~hospitalization was 94.51% (aHR 0.06, 95%CI 0.06, 0.07) lower for the partly vaccinated and 93.49% (aHR 0.07, 95%CI 0.06, 0.07) lower for the fully vaccinated when compared with the non-vaccinated. The risk of ~~hospitalisation~~hospitalization was higher for males (aHR 1.17, 95%CI 1.13, 1.12), those aged 65 and above (aHR 17.27, 95% CI 15.78, 18.89), and those with 1 or more ~~chronic conditions~~comorbidity (aHR 3.33, 95%CI 3.18, 3.50). Individuals from all ~~the~~ other provinces had a higher risk of COVID-19 ~~utilisation~~ ~~hospitalisation~~hospitalization than individuals from the Eastern Cape, the reference group.

Table 2. Univariate and multivariate analysis of impact of COVID-19 vaccination on risk of ~~COVID-19~~ ~~related~~ ~~hospitalisation~~hospitalization for COVID-19 infection

Variable	# of Hosp Events		Hazard Ratio for ~~Hospitalisation~~Hospitalization	
	N	Events/1000 Individuals/ per annum**	UHR (95% CI)	AHR (95% CI)
**Vaccination Status***				
No~~t~~n vaccinated	9,972	10.29	Reference	Reference
Partly vaccinated	848	4.47	0.17 (0.15, 0.18)	0.06 (0.06, 0.07)
Fully vaccinated	791	1.97	0.20 (0.19, 0.22)	0.07 (0.06, 0.07)
**Sex**				
Female	6,145	7.35	Reference	Reference
Male	5,466	7.56	1.05 (1.01, 1.09)	1.17 (1.13, 1.22)
**Age**				
Less than 18	927	1.73	Reference	Reference
Between 18-25	271	2.38	1.33 (1.16, 1.53)	1.99 (1.73, 2.29)
Between 25-40	2,407	5.86	3.49 (3.23, 3.78)	5.04 (4.66, 5.46)
Between 40-65	5,431	13.67	7.64 (7.11, 8.22)	10.32 (9.55, 11.14)
Greater than 65	2,575	25.62	13.93 (12.84, 15.11)	17.27 (15.78, 18.89)
**COVID-19 Comorbidities** ~~**Chronic Condition?Comorbidities**~~				
No	4,303	3.60	Reference	Reference
Yes	7,308	20.12	4.94 (4.74, 5.15)	3.33 (3.18, 3.50)
**Province**				
Eastern Cape	827	7.17	Reference	Reference
Free State	461	7.28	1.08 (0.95, 1.22)	1.26 (1.12, 1.43)
Gauteng	4,812	6.80	0.95 (0.88, 1.03)	1.33 (1.22, 1.44)
KwaZulu-Natal	2,084	8.60	1.21 (1.11, 1.32)	1.30 (1.19, 1.42)
Limpopo	271	4.93	0.74 (0.64, 0.85)	1.04 (0.90, 1.21)
Mpumalanga	320	6.10	0.90 (0.78, 1.03)	1.02 (0.88, 1.17)
North-West	419	7.76	1.14 (1.00, 1.30)	1.33 (1.17, 1.52)
Northern Cape	242	8.54	1.27 (1.09, 1.48)	1.42 (1.22, 1.66)
Western Cape	2,175	9.02	1.26 (1.15, 1.37)	1.32 (1.21, 1.44)
**Total**	11,611	7.45		

Vaccination Status*: Status at time of hospital event.

Events/1000 Individuals/per annum**: Total events/ number of individuals/study period (2.83 years) *1000

Hospital ~~Utilisation~~ Utilization for COVID-19 infection

~~The o~~Overall ~~hospitilisation~~hospital ~~utilisation~~utilization ~~rate~~ for COVID-19 infection ~~related hospital utilisation rate~~ was 69.22 (95%CI 66.87, 71.50) days per 1000 individuals per year (Table 3). The ~~utilisation~~ utilization rate was 93.82 (95%CI 90.31,96.72) for the non-vaccinated, 44.11 (95%CI 37.06, 48.21) for the partly vaccinated and 20.52 (95%CI 17.25, 24.69) for the fully vaccinated. Across the other predictor variables, males (73.78, 95%CI: 70.21, 77.26), individuals aged 65 and above (322.4, 95%CI 295.21, 344.52), those with ~~chronic conditions~~comorbidities (214.87, 95%CI 204.78, 222.94) and those from the Western Cape (86.56, 95%CI 79.41, 93.82) had the highest hospital ~~hospitalisation utilisation~~ utilization rates.

In multivariate analysis, after adjustment for all other factors, the results from the count component of the model indicated that compared with the non-vaccinated, hospital ~~utilisation~~ utilization was 17.70% (95% CI -24.78%, -9.95%) lower for the partly vaccinated and 20.04% (95% CI -28.26%, -10.88%) lower for the fully- vaccinated. The zero inflated component results indicated that, compared with the non-vaccinated, the relative risk of zero hospital days was 4.34 (95% CI 4.02, 4.68) for the partly vaccinated increasing to 18.55 (95% CI 17.12, 20.11) for the fully vaccinated. Across the other predictor variables, the results from the count component indicated that hospital ~~utilisation~~ utilization was 10.87% (95% CI 5.38%, 16.64%) higher for males compared with females, 198.39% (95% CI 156.51%, 247.10%) higher for those greater than 65-year-old compared with under 18's, and 24.82% (95% CI 17.99%, 32.05%) higher for those with ~~chronic conditions~~comorbidities compared with those without. The zero inflated component results indicated that the relative risk of zero hospital days was 0.87 (95% CI 0.84, 0.91) for males compared with females, 0.06 (95% CI 0.06, 0.07) for those greater than 65-year-old compared with the under 18's, 0.26 (95% CI 0.25, 0.27) for those with a ~~chronic condition~~comorbidity compared

~~with~~ to those without and 0.74 (95% CI 0.68, 0.82) for those from the Western Cape relative to the Eastern Cape.

Table 3. Univariate and multivariate analysis of impact of COVID-19 vaccination on ~~COVID-19~~ hospital ~~utilisation~~ utilization for COVID-19 infection

	Hospital Days			% Difference in Hospital Days vs Reference Group***		Risk Ratio of Zero Hospital Day vs. Reference	
	Number of days	Days/ Event	Days/ 1000 individuals/ year**	Unadjusted	Adjusted	Unadjusted	Adjusted
**Vaccination Status***							
Non vaccinated	91 738	9.19	93.82	Reference	Reference	Reference	Reference
Partly vaccinated	7 976	9.41	44.11	-0.54% (-9.26%, 9.02%)	-17.70% (-24.78%, -9.95%)	2.29 (2.12, 2.47)	4.34 (4.02, 4.68)
Fully vaccinated	8 219	10.39	20.52	0.17% (-10.39%, 11.98%)	-20.04% (-28.26%, -10.88%)	5.19 (4.80, 5.62)	18.55 (17.12, 20.11)
**Sex**							
Female	54 582	8.88	65.28	Reference	Reference	Reference	Reference
Male	53 351	9.76	73.78	11.90% (6.27%, 17.83%)	10.87% (5.38%, 16.64%)	0.98 (0.94, 1.02)	0.87 (0.84, 0.91)
**Age**							
Less than 18	3 870	4.17	7.21	Reference	Reference	Reference	Reference
Between 18-25	1 479	5.46	12.99	39.54% (6.54%, 82.77%)	38.10% (7.47%, 77.45%)	0.77 (0.67, 0.90)	0.57 (0.49, 0.66)
Between 25-40	15 518	6.45	37.77	69.91% (45.38%, 98.58%)	65.55% (42.17%, 92.77%)	0.32 (0.29, 0.35)	0.25 (0.23, 0.27)
Between 40-65	54 658	10.06	137.54	176.56% (138.65%, 220.50%)	152.54% (118.19%, 192.29%)	0.14 (0.13, 0.15)	0.12 (0.11, 0.13)
Greater than 65	32 408	12.59	322.40	228.82% (182.87%, 282.24%)	198.39% (156.51%, 247.10%)	0.07 (0.07, 0.08)	0.06 (0.06, 0.07)
**COVID-19 Comorbidities**							
No	29 875	6.94	24.98	Reference	Reference	Reference	Reference
Yes	78 058	10.68	214.87	55.32% (47.18%, 63.91%)	24.82% (17.99%, 32.05%)	0.18 (0.17, 0.19)	0.26 (0.25, 0.27)
**Province**							
Eastern Cape	8 175	9.89	70.90	Reference	Reference	Reference	Reference
Free State	4 449	9.65	70.27	-2.58% (-16.17%, 13.21%)	-0.18% (-14.02%, 15.89%)	0.98 (0.86, 1.11)	0.84 (0.74, 0.96)
Gauteng	46 851	9.74	66.24	-6.42% (-14.71%, 2.68%)	-0.88% (-9.52%, 8.60%)	1.05 (0.96, 1.13)	0.76 (0.70, 0.83)
KwaZulu-Natal	17 369	8.33	71.64	-19.14% (-27.01%, -10.42%)	-11.32% (-19.85%, -1.89%)	0.81 (0.74, 0.88)	0.74 (0.67, 0.81)
Limpopo	1 981	7.31	36.02	-28.91% (-42.39%, -12.27%)	-18.05% (-31.78%, -1.55%)	1.40 (1.21, 1.63)	0.97 (0.83, 1.13)
Mpumalanga	2 816	8.80	53.68	-12.15% (-27.30%, 6.15%)	-5.67% (-20.69%, 12.21%)	1.16 (1.01, 1.33)	0.98 (0.85, 1.13)
North West	3 441	8.21	63.70	-18.76% (-29.19%, -6.78%)	-16.46% (-26.91%, -4.53%)	0.90 (0.79, 1.03)	0.75 (0.66, 0.86)
Northern Cape	1 986	8.21	70.08	-18.82% (-32.45%, -2.45%)	-16.05% (-30.38%, 1.24%)	0.81 (0.70, 0.95)	0.70 (0.60, 0.83)
Western Cape	20 865	9.59	86.56	-6.88% (-16.40%, 3.72%)	-8.83% (-17.66%, 0.95%)	0.78 (0.72, 0.85)	0.74 (0.68, 0.82)
**Total**	107 933	9.30	69.22				

Vaccination Status*: Status as time of hospital event if hospitilised, and as end of study period if never hospitilised during study period

Days/1000 Individuals/per annum**: Total days/ number of individuals/study period (2.83 years) *1000

% Difference in hospital days***: Exponent of estimated coefficents (beta1) from count component output from negative binonmial model - 1 X 100: (exp(beta1)-1)x100.

Risk Ratio for zero hospital days****: Exponent of estimated coefficents (beta1) from zero inflated component output from negative binonmial model : exp(beta1)

	Hospital Days			% Difference in Hospital Days vs Reference Group***		Risk Ratio of Zero Hospital Day vs. Reference	
	Number of days	Days/ Event	Days/ 1000 individuals/ year**	Unadjusted	Adjusted	Unadjusted	Adjusted
**Vaccination Status***							
Not vaccinated	91,738	9.19	93.82	Reference	Reference	Reference	Reference
Partly vaccinated	7,976	9.41	44.11	-0.54% (-9.26%, 9.02%)	-17.70% (-24.78%, -9.95%)	2.29 (2.12, 2.47)	4.34 (4.02, 4.68)
Fully vaccinated	8,219	10.39	20.52	0.17% (-10.39%, 11.98%)	-20.04% (-28.26%, -10.88%)	5.19 (4.80, 5.62)	18.55 (17.12, 20.11)
**Sex**							
Female	54,582	8.88	65.28	Reference	Reference	Reference	Reference
Male	53,351	9.76	73.78	11.90% (6.27%, 17.83%)	10.87% (5.38%, 16.64%)	0.98 (0.94, 1.02)	0.87 (0.84, 0.91)
**Age**							
Less than 18	3,870	4.17	7.21	Reference	Reference	Reference	Reference
Between 18-25	1,479	5.46	12.99	39.54% (6.54%, 82.77%)	38.10% (7.47%, 77.45%)	0.77 (0.67, 0.90)	0.57 (0.49, 0.66)
Between 25-40	15,518	6.45	37.77	69.91% (45.38%, 98.58%)	65.55% (42.17%, 92.77%)	0.32 (0.29, 0.35)	0.25 (0.23, 0.27)
Between 40-65	54,658	10.06	137.54	176.56% (138.65%, 220.50%)	152.54% (118.19%, 192.29%)	0.14 (0.13, 0.15)	0.12 (0.11, 0.13)
Greater than 65	32,408	12.59	322.40	228.82% (182.87%, 282.24%)	198.39% (156.51%, 247.10%)	0.07 (0.07, 0.08)	0.06 (0.06, 0.07)
**Chronic Condition?**							
No	29,875	6.94	24.98	Reference	Reference	Reference	Reference
Yes	78,058	10.68	214.87	55.32% (47.18%, 63.91%)	24.82% (17.99%, 32.05%)	0.18 (0.17, 0.19)	0.26 (0.25, 0.27)
**Province**							
Eastern Cape	8,175	9.89	70.90	Reference	Reference	Reference	Reference
Free State	4,449	9.65	70.27	-2.58% (-16.17%, 13.21%)	-0.18% (-14.02%, 15.89%)	0.98 (0.86, 1.11)	0.84 (0.74, 0.96)
Gauteng	46,851	9.74	66.24	-6.42% (-14.71%, 2.68%)	-0.88% (-9.52%, 8.60%)	1.05 (0.96, 1.13)	0.76 (0.70, 0.83)
KwaZulu-Natal	17,369	8.33	71.64	-19.14% (-27.01%, -10.42%)	-11.32% (-19.85%, -1.89%)	0.81 (0.74, 0.88)	0.74 (0.67, 0.81)
Limpopo	1,981	7.31	36.02	-28.91% (-42.39%, -12.27%)	-18.05% (-31.78%, -1.55%)	1.40 (1.21, 1.63)	0.97 (0.83, 1.13)
Mpumalanga	2,816	8.80	53.68	-12.15% (-27.30%, 6.15%)	-5.67% (-20.69%, 12.21%)	1.16 (1.01, 1.33)	0.98 (0.85, 1.13)
North West	3,441	8.21	63.70	-18.76% (-29.19%, -6.78%)	-16.46% (-26.91%, -4.53%)	0.90 (0.79, 1.03)	0.75 (0.66, 0.86)
Northern Cape	1,986	8.21	70.08	-18.82% (-32.45%, -2.45%)	-16.05% (-30.38%, 1.24%)	0.81 (0.70, 0.95)	0.70 (0.60, 0.83)
Western Cape	20,865	9.59	86.56	-6.88% (-16.40%, 3.72%)	-8.83% (-17.66%, 0.95%)	0.78 (0.72, 0.85)	0.74 (0.68, 0.82)
**Total**	107,933	9.30	69.22				

Vaccination Status*: Status as time of hospital event if hospitilised, and as end of study period if never hospitilised during study period

Days/1000 Individuals/per annum**: Total days/ number of individuals/study period (2.83 years) *1000

% Difference in hospital days***: Exponent of estimated coefficents (beta1) from count component output from negative binonmial model - 1 X 100: (exp(beta1)-1)x100.

Risk Ratio for zero hospital days****: Exponent of estimated coefficents (beta1) from zero inflated component output from negative binonmial model : exp(beta1)

Hospital Expenditure for COVID-19 infection

For the overall study population, ~~The overall~~ hospital expenditure per individual ~~(expenditure per individual per year)~~ for COVID-19 infection ~~related hospital expenditure (expenditure per individual per year) for the study population~~ was ~~USD~~$12.39 (95%CI $11.15, $13.61) (Table 4), with ~~. The expenditure was USD~~$17.85~~8~~ (95%CI $16.81, $18.77) per annum for the non-vaccinated ~~individuals~~, ~~USD~~$6.19 (95%CI $5.20, $6.76) for the partly vaccinated ~~individuals~~, and ~~USD~~$1.86 ~~2~~(95%CI $1.62, $2.18) for fully vaccinated individuals. Across the other predictor variables, males (~~USD~~$13.25, 95%CI $12.26, $14.23), individuals aged 65 and above (~~USD~~$110.17, 95%CI $109.18, $111.15), those with ~~chronic conditions~~comorbidities (~~USD~~$37.57, 95%CI $36.59,$38.55~~8~~) and those from the Western Cape

(~~USD~~$16.22, 95%CI $15.23, $17.21) had the highest ~~hospitalisation~~ hospital expenditure for COVID-19 infection.

In multivariate analysis, after adjustment for all other factors, the results from the count component of the model indicated that hospital expenditure was 32.83% (95% CI -41.06%, -23.44%) -lower for the partly vaccinated and 55.29% (95% CI -61.13%, -48.57%) lower for the fully vaccinated when compared with the non-vaccinated. Hospital expenditure~~s~~ ~~were~~ was 9.62% (95% CI 0.37%, 18.57%) higher for males compared with females, 259.12% (95%CI 195.45%, 337.44%) higher for those aged 65 and above compared with under 18's and 19.43% (95%CI 8.29%, 31.71%) higher for those with ~~chronic conditions~~comorbidities compared ~~with~~ to those without. The zero inflated component results indicated that, compared with the non-vaccinated, the relative risk of zero hospital expenditure was 4.38 (95% CI 4.06, 4.73) for the partly vaccinated and 18.61 (95% CI 17.18, 20.16) for the fully vaccinated compared with the non-vaccinated. Across the other predictor variables, the results from the count component indicated that hospital expenditure was 9.75% (95% CI 0.86%, 19.43%) higher for males compared with females, 257.83% (95% CI 194.09%, 335.40%) higher for those aged greater than 65-year-old compared with the under 18's, 19.44% (8.31%, 31.72%) higher for those with ~~chronic conditions~~comorbidities compared ~~with~~ to those without and 38.59% (95% CI 17.77%, 63.10%) higher for those from the Western Cape compared to those from the Eastern Cape. The zero inflated component results indicated that the relative risk of zero hospital expenditure was 0.86 (95% CI 0.83, 0.90) for males compared with females, 0.06 (95% CI 0.05, 0.06) for those aged greater than– 65-year-old compared with- the under 18's, 0.25 (0.24, 0.27) for those with ~~chronic conditions~~comorbidities compared to those without, and 0.75 (95% CI 0.69, 0.83) for those from the Western Cape compared with the Eastern Cape.

Table 4. Univariate and multivariate analysis of impact of COVID-19 vaccination on ~~COVID-19~~ hospital expenditure for COVID-19 infections.~~xpenditures.~~

	Hospital Expenditure**			% Difference in Hospital expenditure vs Reference Group***		Risk Ratio of Zero Hospital Expenditure vs.	
	US Dollars(m's)	US Dollars /Event	US Dollars/ Individual/ Annum***	Unadjusted	Adjusted	Unadjusted	Adjusted
**Vaccination Status***							
Non vaccinated	17.45	1 749	17.85	Reference	Reference	Reference	Reference
Partly vaccinated	1.12	1 319	6.19	-25.30% (-34.09%, -15.33%)	-32.83% (-41.06%, -23.44%)	2.28 (2.11, 2.46)	4.38 (4.06, 4.73)
Fully vaccinated	0.75	943	1.86	-48.35% (-55.11%, -40.57%)	-55.29% (-61.13%, -48.57%)	5.15 (4.76, 5.56)	18.61 (17.18, 20.16)
**Sex**							
Female	9.74	1 585	11.65	Reference	Reference	Reference	Reference
Male	9.58	1 752	13.25	10.77% (0.69%, 21.86%)	9.75% (0.86%, 19.43%)	0.97 (0.93, 1.01)	0.86 (0.83, 0.90)
**Age**							
Less than 18	0.47	506	0.87	Reference	Reference	Reference	Reference
Between 18-25	4.58	16 917	40.26	60.42% (2.42%, 151.26%)	68.23% (15.25%, 145.57%)	0.73 (0.64, 0.84)	0.54 (0.47, 0.62)
Between 25-40	0.22	91	0.53	146.40% (100.00%, 203.56%)	142.25% (99.61%, 194.00%)	0.29 (0.27, 0.32)	0.23 (0.21, 0.25)
Between 40-65	2.97	547	7.48	308.13% (237.32%, 393.80%)	286.26% (222.83%, 362.17%)	0.12 (0.12, 0.13)	0.11 (0.10, 0.11)
Greater than 65	11.07	4 301	110.17	254.45% (189.76%, 333.59%)	257.83% (194.09%, 335.40%)	0.06 (0.06, 0.07)	0.06 (0.05, 0.06)
**COVID-19 Comorbidities**							
No	5.67	1 317	4.74	Reference	Reference	Reference	Reference
Yes	13.65	1 868	37.57	41.64% (28.51%, 56.12%)	19.44% (8.31%, 31.72%)	0.17 (0.16, 0.18)	0.25 (0.24, 0.27)
**Province**						Reference	
Eastern Cape	1.07	1 298	9.31	Reference	Reference		Reference
Free State	0.72	1 568	11.42	21.42% (-6.59%, 57.83%)	27.82% (-3.88%, 69.96%)	0.99 (0.87, 1.12)	0.85 (0.75, 0.96)
Gauteng	8.03	1 668	11.35	28.58% (8.92%, 51.79%)	33.42% (14.57%, 55.38%)	1.05 (0.97, 1.14)	0.77 (0.71, 0.83)
KwaZulu-Natal	3.90	1 869	16.07	44.15% (20.02%, 73.14%)	49.79% (26.43%, 77.46%)	0.83 (0.76, 0.91)	0.75 (0.69, 0.82)
Limpopo	0.25	914	4.50	-29.88% (-51.05%, 0.45%)	-23.55% (-43.95%, 4.28%)	1.45 (1.25, 1.68)	0.99 (0.85, 1.15)
Mpumalanga	0.41	1 277	7.79	-1.71% (-28.12%, 34.42%)	2.38% (-23.02%, 36.15%)	1.17 (1.02, 1.35)	0.99 (0.85, 1.14)
North West	0.60	1 435	11.13	10.62% (-11.74%, 38.64%)	4.30% (-14.95%, 27.90%)	0.92 (0.81, 1.05)	0.77 (0.67, 0.88)
Northern Cape	0.43	1 774	15.15	36.96% (-5.53%, 98.55%)	45.66% (-5.35%, 124.16%)	0.83 (0.71, 0.98)	0.72 (0.61, 0.85)
Western Cape	3.91	1 798	16.22	38.38% (15.70%, 65.50%)	38.59% (17.77%, 63.10%)	0.79 (0.72, 0.86)	0.75 (0.69, 0.83)
**Total**	19.32	1 664	12.39				

Vaccination Status*: Status as time of hospital event if hospitilised, and as end of study period if never hospitilised during study period

Hospital Expenditure**: South African Rands converted to US Dollary terms and discounted to 2022 value

USD/Individual/per annum***: Total USD/ number of individuals/study period (2.83 years)

% Difference in hospital expenditure***: Exponent of estimated coefficents (beta1) from count component output from negative binonmial model - 1 X 100: (exp(beta1)-1)x100.

Risk Ratio for zero hospital expenditure****: Exponent of estimated coefficents (beta1) from zero inflated component output from negative binonmial model : exp(beta1)

	Hospital Expenditure**			% Difference in Hospital expenditure vs Reference Group***		Risk Ratio of Zero Hospital Expenditure vs.	
	USD(m's)	USD/Event	USD/ Individual/ Annum***	Unadjusted	Adjusted	Unadjusted	Adjusted
**Vaccination Status***							
Not vaccinated	17.45	1,749	18	Reference	Reference	Reference	Reference
Partly vaccinated	1.12	1,319	6	-25.30% (-34.09%, -15.33%)	-32.83% (-41.06%, -23.44%)	2.28 (2.11, 2.46)	4.38 (4.06, 4.73)
Fully vaccinated	0.75	943	2	-48.35% (-55.11%, -40.57%)	-55.29% (-61.13%, -48.57%)	5.15 (4.76, 5.56)	18.61 (17.18, 20.16)
**Sex**							
Female	9.74	1,585	12	Reference	Reference	Reference	Reference
Male	9.58	1,752	13	10.77% (0.69%, 21.86%)	9.75% (0.86%, 19.43%)	0.97 (0.93, 1.01)	0.86 (0.83, 0.90)
**Age**							
Less than 18	0.47	506	1	Reference	Reference	Reference	Reference
Between 18-25	4.58	16,917	40	60.42% (2.42%, 151.26%)	68.23% (15.25%, 145.57%)	0.73 (0.64, 0.84)	0.54 (0.47, 0.62)
Between 25-40	0.22	91	1	146.40% (100.00%, 203.56%)	142.25% (99.61%, 194.00%)	0.29 (0.27, 0.32)	0.23 (0.21, 0.25)
Between 40-65	2.97	547	7	308.13% (237.32%, 393.80%)	286.26% (222.83%, 362.17%)	0.12 (0.12, 0.13)	0.11 (0.10, 0.11)
Greater than 65	11.07	4,301	110	254.45% (189.76%, 333.59%)	257.83% (194.09%, 335.40%)	0.06 (0.06, 0.07)	0.06 (0.05, 0.06)
**Chronic Condition?**							
No	5.67	1,317	5	Reference	Reference	Reference	Reference
Yes	13.65	1,868	38	41.64% (28.51%, 56.12%)	19.44% (8.31%, 31.72%)	0.17 (0.16, 0.18)	0.25 (0.24, 0.27)
**Province**						Reference	
Eastern Cape	1.07	1,298	9	Reference	Reference		Reference
Free State	0.72	1,568	11	21.42% (-6.59%, 57.83%)	27.82% (-3.88%, 69.96%)	0.99 (0.87, 1.12)	0.85 (0.75, 0.96)
Gauteng	8.03	1,668	11	28.58% (8.92%, 51.79%)	33.42% (14.57%, 55.38%)	1.05 (0.97, 1.14)	0.77 (0.71, 0.83)
KwaZulu-Natal	3.90	1,869	16	44.15% (20.02%, 73.14%)	49.79% (26.43%, 77.46%)	0.83 (0.76, 0.91)	0.75 (0.69, 0.82)
Limpopo	0.25	914	5	-29.88% (-51.05%, 0.45%)	-23.55% (-43.95%, 4.28%)	1.45 (1.25, 1.68)	0.99 (0.85, 1.15)
Mpumalanga	0.41	1,277	8	-1.71% (-28.12%, 34.42%)	2.38% (-23.02%, 36.15%)	1.17 (1.02, 1.35)	0.99 (0.85, 1.14)
North West	0.60	1,435	11	10.62% (-11.74%, 38.64%)	4.30% (-14.95%, 27.90%)	0.92 (0.81, 1.05)	0.77 (0.67, 0.88)
Northern Cape	0.43	1,774	15	36.96% (-5.53%, 98.55%)	45.66% (-5.35%, 124.16%)	0.83 (0.71, 0.98)	0.72 (0.61, 0.85)
Western Cape	3.91	1,798	16	38.38% (15.70%, 65.50%)	38.59% (17.77%, 63.10%)	0.79 (0.72, 0.86)	0.75 (0.69, 0.83)
**Total**	19.32	1,664	12				

Vaccination Status*: Status as time of hospital event if hospitilised, and as end of study period if never hospitilised during study period

Hospital Expenditure**: South African Rands converted to US Dollary terms and discounted to 2022 value

USD/Individual/per annum***: Total USD/ number of individuals/study period (2.83 years)

% Difference in hospital expenditure***: Exponent of estimated coefficents (beta1) from count component output from negative binonmial model - 1 X 100: (exp(beta1)-1)x100.

Risk Ratio for zero hospital expenditure****: Exponent of estimated coefficents (beta1) from zero inflated component output from negative binonmial model : exp(beta1)

Discussion

This study has estimated the impact of COVID-19 vaccination on hospitalization for COVID-19 infection based on a retrospective cohort analysis of 550,332 individuals enrolled in two private health insurance schemes during the period of the pandemic. In contrast to many studies that have reported on the efficacy of the vaccine in -clinical trial situations, in population sub-sets using test-negative controls[27], on specific virusesCOVID-19 variants and/or vaccines, this study examined and provides insights on the impact of the COVID-19 vaccination on an aggregated basis in- a- "real world" population.

Field Code Changed

By the end of the study period, 12.2% of the study cohort was partiallypartly vaccinated and 25.7% was fully vaccinated. The overall COVID-19 related hospitalisationhospitalization rate for COVID-19 infection for the period was 7.45 events per 1000 individuals per yearannum, the hospital utilisation utilization rate was 69.22 days per 1000 individuals per yearannum, and hospital expenditure was USD$12 per individual per annum.

While the results of the study are arguably generalizable to the insured population, generalizability to the broader South African population is limited given differences in demographics, socio-economic profiles, access to care and clinical guidelines between the insured and non-insured populations.

Formatted: Not Highlight

Across all analyses and after adjustment for other risk factors, the study found significantly lower hospitalization events, hospital utilization and hospital expenditure for COVID-19 infection in the partly or fully vaccinated in comparison to the non-vaccinated. Each outcome will be discussed in turn.

The findings of this study indicate thatThis study found COVID-19 vaccination substantiallythe reduced the risk of hospitalization for COVID-19 infection to be substantially lower amongst those who had vaccinated for COVID-19. related hospitalisation After adjustment for other risk factors and when compared with the non-vaccinated, theFirst, the study finds that hospitalization risk for COVID-19 infection was 94.51% (aHR 0.06, 95%CI 0.06, 0.07) lower for the partly vaccinated and 93.49% (aHR 0.07, 95%CI 0.06, 0.07) lower for the fully vaccinated. The These findings are in line with support the existing literaturestudy findings on the impact of vaccination on hospitalisation risk are comparable to are in line with the findings of other studies. . A An observational cohort study of >2 million adults in the Western Cape Province of South Africa over the period 2020 to 2022 reported vaccine effectiveness of 45-92% for hospital admission with severe disease or death, and 25-90% for any admission or

death[29]. Another South African study examining the impact of the J&J vaccine in health workers reported vaccine effectiveness of of 75% (95% CI: 69%-82%) in preventing COVID-19-related hospital admissions requiring critical or intensive care, and 67% (95% CI: 62%-71%) in preventing COVID-19-related hospitalisationhospitalization[12]. More broadly, at least 3 meta- analyses have reported similar results. The first, including , incorporating 51 studies to assess assessing vaccine effectiveness in the "real-world" found that reported the COVID-19 vaccines were to be 97.2% (95% CI 96.1-98.3%) effective in preventing against hospitalisationhospitalization and 97.4% (95% CI 96.0-98.8%) effective against admission to the intensive care unit [28]. The second, a review of 58 studies, reported a single dose of vaccine to be 66% (50%–81%) effective in preventing hospitalisationhospitalizationwhile ., and 45% (42%–49%) effective in preventing Intensive Care Unit (ICU) admissions. The review reported that two doses of the vaccine werewere 93% (89%–96%) effective in preventing hospitalisationhospitalization and 96% (93%–98%) effective in preventing ICU admissions[29]. The third, a systematic review of 42 studies examining the efficacy of a range of COVID-19 vaccines in a variety of populations, concluded that the Pfizer/BioNTech vaccine had >90% effectiveness against infections requiring hospitalisationhospitalization after the second dose. A single dose of the J&J vaccine was >60% effective against infections requiring hospitalisationhospitalization[30].[30].

Second, in terms of hospital utilisation utilization for COVID-19 infection, This study found hospital utilisation for COVID-19 infection to be substantially lower amongst those who had vaccinated for COVID-19.findings indicate that Hospital utilization was 17.70% (95% CI -24.78%, -9.95%) lower for the partly vaccinated and 20.04% (95% CI -28.26%, -10.88%) lower for the fully vaccinated; while the relative risk of zero hospital days was 4.34 (95% CI 4.02, 4.68) higher for the partly vaccinated and 18.55 (95% CI 17.12, 20.11) higher for the fully vaccinated. This compares to a reported reduction in the length of hospital stay of 38% (95% CI: 19%–52%) byin a Norwegian study carried out in Norway[31], and athe reduction of 14% (95 %CI -24%, 4%) reduction reported in a Chinese by a study in China [32].

Formatted: Highlight

Formatted: Highlight

Third, in terms of hospital expenditure for This study also found hospital expenditure for COVID-19 infection to be substantially lower amongst those who had vaccinated for COVID-19 infection, findings indicate that expenditure was. , hospital utilisation and hospital expenditure in the study population. After adjustment for other risk factors and when compared with the non-vaccinated, the hospitalisation risk was 94.51% lower for the partly vaccinated and 93.49% lower for the fully vaccinated; hospital utilisation was 17.70% lower for the partly vaccinated and 20.04% lower for the fully vaccinated; the relative risk of zero hospital days was 4.33 for the partly vaccinated and 18.55 for the fully vaccinated; hA retrospective study based on electronic health records of more than 180 for-profit hospitals across 20 states in the United States and comprising 29,732 (21,525 unvaccinated and 8,207 vaccinated) patients admitted with COVID-19 from January to December 2021 reported vaccinated patients having a 2.13 (CI 2.73–1.55 days) lower average length of stay (LoS) when compared with non-vaccinated[35]. A retrospective cohort study of 1927 hospitalized patients infected with COVID-19 in a tertiary-level public hospital in the Community of Madrid (Spain) between 1 January and 31 July 2021 found a reduction in hospital stay in vaccinated patients of 3.17 days (CI 95%: 5.88 to 0.47)[36]. A study on COVID-19 positive patients (716 fully vaccinated and 2487 unvaccinated) hospitalized in Norway between 1 February and 30 November 2021 found that, after adjustment, the LoS of fully vaccinated patients was 38% (95% CI: 19%–52%) shorter than the non-vaccinated[33]. A retrospective cohort study of patients with COVID-19 admitted to a hospital in China (222 vaccinated, 327 unvaccinated) between January 2023 and February 2023, reported an after-adjustment reduction in the LoS of 1.2 days (14% 95 %CI -24%, 4%) [34]. In terms of evidence from LMICs, a retrospective observational study carried out in a tertiary care hospital in western India, from April 1 to June 30 2021, reported unadjusted ward LoS of 6.21 ± 3.20 for vaccinated patients and 5.56 ± 4.55 days ($p < 0.001$) for non-vaccinated patients. The unadjusted mean length of ICU stay for vaccinated patients was reported to be 4.47 ± 2.3 days for vaccinated patients versus 6.29 ± 2.19 days ($p < 0.001$) for non-vaccinated patients [37]. A retrospective

Field Code Changed

Field Code Changed

Field Code Changed

Field Code Changed

cohort study of 1407 patients (812 vaccinated, 1029 unvaccinated) admitted with COVID-19 at a hospital in Karachi, Pakistan reported an unadjusted median and (interquartile range) length of hospital stay of 7 (4–12) days for unvaccinated COVID-19 patients compared with 6 (4–11) days in the vaccinated group[38]. Hospital expenditure for COVID-19 infection was 32.83%(95% CI -41.06%, -23.44%) lower for the partly vaccinated and 55.29% (95% CI -61.13%, -48.57%) lower for the fully vaccinated; and the relative risk of zero hospital expenditure was 4.38 (95% CI 4.06, 4.73) higher for the partly vaccinated and 18.61 (95% CI 17.18, 20.16) higher for the fully vaccinated. In the only study we were able to find reporting on the impact of vaccination on hospital expenditures, a retrospective cohort study carried out on individuals hospitalized for COVID-19 between April 2021 and January 2022carried out at a large academic medical center in the United States of America, the difference in the unadjusted cost of hospitalisation for the vaccinated ($119,630) compared with the unvaccinated ($191,146) was not statistically significant (P=0.06), after adjusting for age and comorbidities. Vvaccinated patients experienced a 26% lower cost of hospitalisationhospitalization compared with the non-unvaccinated patients (P = 0.004). [33].

This analysis study has several limitations. WWhile the results of the study are arguably generalizable to the insured population, generalizability to the broader South African population is limited given differences in demographics, socio-economic profiles, access to care and clinical guidelines between the insured and non-insured populations.

Formatted: Not Highlight

Firstly, The analysis is limited by the fact that it istThe analysis uses based on the routine real world demographic and claims data collected by the health insurance funds as part of their business, not data collected for research purposes pe se. Only services and vaccines for which claims were submitted were analysed. The implications are that both COVID-19 vaccination events and hospitalization for COVID-19

infection could be over or underreported. While it is hard to predict the impact of these biases, it seems plausible that they would not negate the findings entirely, given the magnitude of the impact. In addition, the available demographic and claims data did not enable an analysis of severity of COVID-19 infection. orSurvival analysis or COVID-19 -related mortality related assessments were also not possible due to limitations of the mortality data. an assessment of mortality. Although rare, evidence Evidence now indicates that although rare, there are concerns around safety and side-effects of the COVID-19 vaccines[34, 35]. These were not taken into consideration in this study. Given that this Since this is a retrospective observational cohort study, subject loss-to-follow up was not really a concern. However, the observational nature of the study may have resulted in selection and confounding biases which have not been accounted for and the cohort may therefore suffer from selection bias. Only services and vaccines for which claims were submitted were analysed. This could have resulted in under-recording of COVID-19 vaccinations which could have biased the reported outcome, but it is difficult to comment on the direction of the bias. However, if the bias resulted in an overstatement of the impact of the vaccine, it is unlikely that it would negate it entirely, given the magnitude of the impact. Due to data limitations, the severity of the disease in the individuals hospitalized for COVID-19 could not be factored into the analysis. The analysis aimed to provide policy-relevant insights into the overall impact of Covid-19 vaccinations on three key outcomes and hence provides results at a macro aggregated level. This aggregation comes at the expense of providing a more granular view on the specific components of the pandemic (e.g. different variants of the virus, the different vaccines etc.) as has been done in most other studies. Evidence now indicates that although rare, there are concerns around safety and side-effects of the COVID-19 vaccines[40, 41]. These were not taken into consideration in this study. While the results of the study are arguably generalizable to the insured population, generalizability to the broader South African population is limited given the differences in demographics, socio-economic profiles, and access to care between the insured and non-insured populations. There are also

Field Code Changed

Field Code Changed

differences in the clinical guidelines governing the public and private sectors in South Africa. Caution should therefore be exercised in generalizinginterpreting the findings of this study. Caution should therefore be exercised in generalizing the findings of this study.

A retrospective study based on electronic health records of more than 180 for-profit hospitals across 20 states in the United States and comprising 29,732 (21,525 unvaccinated and 8,207 vaccinated) patients admitted with COVID-19 from January to December 2021 reported vaccinated patients having a 2.13 (CI 2.73–1.55 days) lower average length of stay (LoS) when compared with non-vaccinated[35]. A retrospective cohort study of 1927 hospitalized patients infected with COVID-19 in a tertiary-level public hospital in the Community of Madrid (Spain) between 1 January and 31 July 2021 found a reduction in hospital stay in vaccinated patients of -3.17 days (CI 95%: 5.88 to 0.47)[36]. A study on COVID-19 positive patients (716 fully vaccinated and 2487 unvaccinated) hospitalized in Norway between 1 February and 30 November 2021 found that, after adjustment, the LoS of fully vaccinated patients was 38% (95% CI: 19%–52%) shorter than the non-vaccinated[33]. A retrospective cohort study of patients with COVID-19 admitted to a hospital in China (222 vaccinated, 327 unvaccinated) between January 2023 and February 2023, reported an after-adjustment reduction in the LoS of 1.2 days (14% 95 %CI -24%, 4%) [34]. In terms of evidence from LMICs, a retrospective observational study carried out in a tertiary care hospital in western India, from April 1 to June 30 2021, reported unadjusted ward LoS of 6.21 ± 3.20 for vaccinated patients and 5.56 ± 4.55 days (p < 0.001) for non-vaccinated patients. The unadjusted mean length of ICU stay for vaccinated patients was reported to be 4.47 ± 2.3 days for vaccinated patients versus 6.29 ± 2.19 days (p < 0.001) for non-vaccinated patients [37]. A retrospective cohort study of 1407 patients (812 vaccinated, 1029 unvaccinated) admitted with COVID-19 at a hospital in Karachi,

Pakistan reported an unadjusted median and (interquartile range) length of hospital stay of 7 (4–12) days for unvaccinated COVID-19 patients compared with 6 (4–11) days in the vaccinated group[38]. In the only study we were able to find reporting on impact of vaccination on hospital expenditures, a retrospective cohort study carried out on individuals hospitalized for COVID-19 between April 2021 and January 2022 at a large academic medical center in the United States of America, the difference in the unadjusted cost of hospitalisation for the vaccinated ($119,630) compared with the unvaccinated ($191,146) was not statistically significant (P=0.06), after adjusting for age and comorbidities. Vaccinated patients experienced a 26% lower cost of hospitalisation compared with unvaccinated patients (P = 0.004). [39].

With the emergence of different variants, differences in variant severity, and with reducing population susceptibility over time either due to immunity because of previous infections or through vaccination, there was a complex interplay of many different factors during the full period of the COVID-19 pandemic. In contrast to many studies that have reported on the efficacy of the vaccine in clinical trial situations, in population sub-sets using test-negative controls[28], on specific viruses and/or vaccines, this study examined and provides insights on the impact of the COVID-19 vaccination on an aggregated basis in a "real world" population.

The findings of the study unequivocally confirmindicate that over the entire period - covering the emergence of the various strains of the virus, different vaccines and vaccine regimens and evolving clinical protocols - COVID-19 vaccination was very effective in reducing the risk of COVID-19 related hospitalisation, hospital utilisation and hospital expenditure for COVID-19 was substantially lower amongst those who vaccinated for COVID-19.

While the results of the study are arguably generalizable to the insured population, generalizability to the broader South African population is limited given the differences in demographics, socio-economic

~~profiles, and access to care between the insured and non-insured populations. There are also differences in the clinical guidelines governing the public and private sectors in South Africa. Caution should therefore be exercised in generalizing the findings of this study.~~

Conclusion

~~The findings of t~~This study, based on a large cohort of insured individuals followed over the entire period of the pandemic~~ using real world data~~, found that ~~confirms that~~ all other things being equal - ~~COVID-19 vaccination substantially reduced~~ the risk of ~~hospitalisation~~hospitalization, hospital ~~utilisation~~ utilization and expenditure for COVID-19 infection was substantially lower amongst those who had vaccinated for COVID-19. The ~~results of this~~ study ~~contribute~~provides ~~to~~ evidence ~~regarding~~ on~~help~~ ~~fill~~filling ~~the knowledge gap on~~ the overall "real-world" impact of COVID-19 vaccination, ~~across the entire pandemic period,~~ particularly in insured populations and those in Sub-Saharan Africa ~~and provides date for further economic evaluation of~~ . ~~It also provides valuable data for assessing the effectiveness, cost, and cost-effectiveness of COVID-19 vaccination in preventing COVID-19-related illness~~. Given the differences in demographics, socio-economic profiles, and access to care between insured and non-insured populations, caution should be exercised in generalizing the findings of this study.

Financial disclosure

The South African Medical Research Council provided support in the form of salaries for authors Geetesh Solanki, and the University of Cape Town for Francesca Little and Susan Cleary. None of these institutions had any additional role in the study design, data collection and analysis, decision to publish, or preparation of the manuscript. The specific roles of these authors are articulated in the 'author contributions' section." Geetesh Solanki is also employed on a contractual basis by NMG Consultants and Actuaries, an independent consulting firm providing consulting and actuarial services to South African private health insurance funds. NMG provided access to the data used for the study but did not have any other role or involvement in the study design, data collection and analysis, decision to publish, or preparation of the manuscript. Geetesh Solanki's commercial affiliation to NMG therefore played no role in influencing the study.

Competing Interests

Geetesh Solanki is employed on a contractual basis by the South African Medical Research Council and NMG Consultants and Actuaries, an independent consulting firm providing consulting and actuarial services to South African private health insurance funds. NMG provided access to the data used for the study but did not have any other role or involvement in the study design, data collection and analysis, decision to publish, or preparation of the manuscript and Geetesh Solanki's commercial affiliation to NMG therefore played no role in influencing the study. Geetesh Solanki's association with NMG does not alter our adherence to PLOS One policies on sharing data and materials.

Ethics Statement

Ethics approval for the use of the secondary database used to carry out this study was granted by the Faculty of Health Sciences Human Research Ethics Committee of the University of Cape Town – approval number HREC Ref: 606/2023. No individual consent was required as the research was based on anonymised secondary claims data. Data for the study were made available as part of NMG Consultants and Actuaries (NMG) commitment to support research initiatives with broader public health significance. The data was accessed in terms of and under the conditions set out in the agreement between NMG and the client schemes and between NMG and the researcher. The terms set out in these agreements ensured that the access to and use of the data is in full compliance with the requirements of the Protection of Personal Information (PoPI) Act in South Africa [42]. All the data were provided in a de-identified format and aggregated at an individual level and the research team had no access to information that would enable the identification of any individual. All findings are presented at an aggregate level and no confidential member, health care provider or scheme information is disclosed. Individual consent for the data was therefore not required.

Formatted: Normal

Author contributions

Geetesh Solanki designed the study, Francesca Little lead the data analysis and Susan Cleary provided critical review oversight. Geetesh Solanki provided guidance in extracting the data from the NMG database. Geetesh Solanki wrote the first draft of the manuscript which was then critically reviewed and revised by the other authors. The corresponding author attests that all listed authors meet authorship criteria and that no others meeting the criteria have been omitted. Geetesh Solanki is the guarantor.

Data Availability

Yes - all data are fully available without restriction. All relevant data are within the manuscript and its Supporting Information files.

Formatted: Normal

Acknowledgements

The authors would like to acknowledge the institutional support to the study authors provided by the South African Medical Research Council (SAMRC). We also acknowledge NMG Consultant and Actuaries for assisting in providing access to the data, to Priya Makanjee for extracting the data, and to Jud Cornell for assistance in fine editing the script.

Formatted: Normal

References

1. Statement on the second meeting of the International Health Regulations (2005) Emergency Committee regarding the outbreak of novel coronavirus (2019-nCoV) [Internet]. Geneva WHO; 2020
2. WHO Director-General's opening remarks at the media briefing on COVID-19 – 11 March 2020 [Internet]. Geneva WHO; 2020
3. Thanh Le T, Andreadakis Z, Kumar A, Gomez Roman R, Tollefsen S, Saville M, et al. The COVID-19 vaccine development landscape. Nat Rev Drug Discov. 2020;19(5):305-6. doi: 10.1038/d41573-020-00073-5. PubMed PMID: 32273591.
4. Dzinamarira T, Nachipo B, Phiri B, Musuka G. COVID-19 Vaccine Roll-Out in South Africa and Zimbabwe: Urgent Need to Address Community Preparedness, Fears and Hesitancy. Vaccines. 2021;9(3). Epub 2021/04/04. doi: 10.3390/vaccines9030250. PubMed PMID: 33809002; PubMed Central PMCID: PMCPMC8000117.
5. World Health Organisation. WHO Coronavirus (COVID-19) Dashboard. https://covid19.who.int/table.
6. Bradshaw D, Dorrington R, Laubscher R, Groenewald P, Moultrie T. COVID-19 and all-cause mortality in South Africa – the hidden deaths in the first four waves. South African Journal of Science. 2022;118(5/6). doi: 10.17159/sajs.2022/13300.
7. Ackah BBB, Woo M, Stallwood L, Fazal ZA, Okpani A, Ukah UV, et al. COVID-19 vaccine hesitancy in Africa: a scoping review. Global Health Research and Policy. 2022;7(1):21. doi: 10.1186/s41256-022-00255-1.
8. Jassat W, Mudara C, Ozougwu L, Tempia S, Blumberg L, Davies M-A, et al. Difference in mortality among individuals admitted to hospital with COVID-19 during the first and second waves in South Africa: a cohort study. The Lancet Global Health. 2021;9(9):e1216-e25. doi: 10.1016/S2214-109X(21)00289-8.
9. Solanki G, Wilkinson T, Bansal S, Shiba J, Manda S, Doherty T. COVID-19 hospitalization and mortality and hospitalization-related utilization and expenditure: Analysis of a South African private health insured population. PLOS ONE. 2022;17(5):e0268025. doi: 10.1371/journal.pone.0268025.
10. Collie S, Nayager J, Bamford L, Bekker LG, Zylstra M, Gray G. Effectiveness and Durability of the BNT162b2 Vaccine against Omicron Sublineages in South Africa. The New England journal of medicine. 2022;387(14):1332-3. Epub 2022/09/15. doi: 10.1056/NEJMc2210093. PubMed PMID: 36103455; PubMed Central PMCID: PMCPMC9511610.
11. Collie S, Champion J, Moultrie H, Bekker LG, Gray G. Effectiveness of BNT162b2 Vaccine against Omicron Variant in South Africa. The New England journal of medicine. 2022;386(5):494-6. Epub 2021/12/30. doi: 10.1056/NEJMc2119270. PubMed PMID: 34965358; PubMed Central PMCID: PMCPMC8757569.
12. Bekker LG, Garrett N, Goga A, Fairall L, Reddy T, Yende-Zuma N, et al. Effectiveness of the Ad26.COV2.S vaccine in health-care workers in South Africa (the Sisonke study): results from a single-arm, open-label, phase 3B, implementation study. Lancet. 2022;399(10330):1141-53. Epub 2022/03/21. doi: 10.1016/s0140-6736(22)00007-1. PubMed PMID: 35305740; PubMed Central PMCID: PMCPMC8930006 ViiV Health Care, and Gilead. .
13. Gray G, Collie S, Goga A, Garrett N, Champion J, Seocharan I, et al. Effectiveness of Ad26.COV2.S and BNT162b2 Vaccines against Omicron Variant in South Africa. New England Journal of Medicine. 2022;386(23):2243-5. doi: 10.1056/NEJMc2202061. PubMed PMID: 35507482.
14. Kassanjee R, Davies M-A, Heekes A, Mahomed H, Hawkridge AJ, Morden E, et al. COVID-19 Vaccine Uptake and Effectiveness by Time since Vaccination in the Western Cape Province, South Africa:

An Observational Cohort Study during 2020–2022. Vaccines. 2024;12(6):628. PubMed PMID: doi:10.3390/vaccines12060628.

15. Vandenbroucke JP, von Elm E, Altman DG, Gøtzsche PC, Mulrow CD, Pocock SJ, et al. Strengthening the Reporting of Observational Studies in Epidemiology (STROBE): Explanation and Elaboration. PLOS Medicine. 2007;4(10):e297. doi: 10.1371/journal.pmed.0040297.

16. Centre for Respiratory Diseases and Meningitis. Proposed definition of COVID-19 wave in South Africa. Communicable Diseases Communiqué. November 2021, Vol. 20 (11). https://www.nicd.ac.za/wp-content/uploads/2021/11/Proposed-definition-of-COVID-19-wave-in-South-Africa.pdf.

17. National Department of Health.. Media Release: Cabinet approves changes to COVID-19 regulations. 30 December 2021. https://sacoronavirus.co.za/2021/12/30/media-release-cabinet-approves-changes-to-covid-19-regulations/.

18. Council for Medical Schemes. Annual Report 2021/22. https://www.medicalschemes.co.za/cms-annual-report-2021-22/.

19. Havers FP, Pham H, Taylor CA, Whitaker M, Patel K, Anglin O, et al. COVID-19-Associated Hospitalizations Among Vaccinated and Unvaccinated Adults 18 Years or Older in 13 US States, January 2021 to April 2022. JAMA Internal Medicine. 2022;182(10):1071-81. doi: 10.1001/jamainternmed.2022.4299.

20. Discovery Health. When are you fully vaccinated? September 2021. https://www.discovery.co.za/corporate/covid19-when-are-you-fully-vaccinated.

21. South African Medical Association. How to Code for COVID-19 and the necessity of legal disclaimers (Updated Article). Med-e-Mail. https://www.samedical.org/cmsuploader/viewArticle/1119.

22. Stats SA. PO141- Consumer Price Index (CPI). CPI History. 11 June 2024. https://www.statssa.gov.za/publications/P0141/CPIHistory.pdf?SCH=73795. .

23. Fathi M, Vakili K, Sayehmiri F, Mohamadkhani A, Hajiesmaeili M, Rezaei tavirani M, et al. The prognostic value of comorbidity for the severity of COVID-19: A systematic review and meta-analysis study. PLOS ONE. 2021;16:e0246190. doi: 10.1371/journal.pone.0246190.

24. Council for Medical Schemes. Guidelines for the Identification of Beneficiaries with Risk Factors in Accordance with the Entry and Verification Criteria. Version 10.1. Pretoria: CMS.

25. StataCorp. 2023. Stata Statistical Software: Release 16.1. College Station, TX: StataCorp LLC.

26. South Africa. Protection of Personal Information Act No. 4 of 2013. https://www.gov.za/documents/promotion-access-information-act.

27. Chua H, Feng S, Lewnard JA, Sullivan SG, Blyth CC, Lipsitch M, et al. The Use of Test-negative Controls to Monitor Vaccine Effectiveness: A Systematic Review of Methodology. Epidemiology (Cambridge, Mass). 2020;31(1):43-64. Epub 2019/10/15. doi: 10.1097/ede.0000000000001116. PubMed PMID: 31609860; PubMed Central PMCID: PMCPMC6888869.

28. Zheng C, Shao W, Chen X, Zhang B, Wang G, Zhang W. Real-world effectiveness of COVID-19 vaccines: a literature review and meta-analysis. International journal of infectious diseases : IJID : official publication of the International Society for Infectious Diseases. 2022;114:252-60. Epub 2021/11/21. doi: 10.1016/j.ijid.2021.11.009. PubMed PMID: 34800687; PubMed Central PMCID: PMCPMC8595975.

29. Liu Q, Qin C, Liu M, Liu J. Effectiveness and safety of SARS-CoV-2 vaccine in real-world studies: a systematic review and meta-analysis. Infectious diseases of poverty. 2021;10(1):132. Epub 2021/11/16. doi: 10.1186/s40249-021-00915-3. PubMed PMID: 34776011; PubMed Central PMCID: PMCPMC8590867.

30. Mohammed I, Nauman A, Paul P, Ganesan S, Chen KH, Jalil SMS, et al. The efficacy and effectiveness of the COVID-19 vaccines in reducing infection, severity, hospitalization, and mortality: a systematic review. Hum Vaccin Immunother. 2022;18(1):2027160. Epub 20220203. doi: 10.1080/21645515.2022.2027160. PubMed PMID: 35113777; PubMed Central PMCID: PMCPMC8862168.

31. Whittaker R, Bråthen Kristofferson A, Valcarcel Salamanca B, Seppälä E, Golestani K, Kvåle R, et al. Length of hospital stay and risk of intensive care admission and in-hospital death among COVID-19 patients in Norway: a register-based cohort study comparing patients fully vaccinated with an mRNA vaccine to unvaccinated patients. Clinical microbiology and infection : the official publication of the European Society of Clinical Microbiology and Infectious Diseases. 2022;28(6):871-8. Epub 2022/02/28. doi: 10.1016/j.cmi.2022.01.033. PubMed PMID: 35219807; PubMed Central PMCID: PMCPMC8872711.
32. Fang X, Tao G, Zhou H, Zhou Y. Vaccines reduced hospital length of stay and fraction of inspired oxygen of COVID-19 patients: A retrospective cohort study. Preventive medicine reports. 2024;39:102632. Epub 2024/02/13. doi: 10.1016/j.pmedr.2024.102632. PubMed PMID: 38348219; PubMed Central PMCID: PMCPMC10859302.
33. Somani ST, Firestone RL, Donnelley MA, Sanchez L, Hatfield C, Fine J, et al. Impact of Vaccination on Cost and Course of Hospitalization Associated with COVID-19 Infection. Antimicrobial stewardship & healthcare epidemiology : ASHE. 2023;3(1):e19. Epub 2023/01/31. doi: 10.1017/ash.2022.364. PubMed PMID: 36714292; PubMed Central PMCID: PMCPMC9879923.
34. Chirico F, Teixeira da Silva JA, Tsigaris P, Sharun K. Safety & effectiveness of COVID-19 vaccines: A narrative review. The Indian journal of medical research. 2022;155(1):91-104. Epub 2022/07/22. doi: 10.4103/ijmr.IJMR_474_21. PubMed PMID: 35859436; PubMed Central PMCID: PMCPMC9552389.
35. Beatty AL, Peyser ND, Butcher XE, Cocohoba JM, Lin F, Olgin JE, et al. Analysis of COVID-19 Vaccine Type and Adverse Effects Following Vaccination. JAMA network open. 2021;4(12):e2140364. Epub 2021/12/23. doi: 10.1001/jamanetworkopen.2021.40364. PubMed PMID: 34935921; PubMed Central PMCID: PMCPMC8696570

Supplementary Tables.

Table S1. Comparison of study population versus rest of the insured in South Africa

Table S2. ~~Hospitilisation~~Hospitalization risk dataset

Table S3. Hospital ~~utilisation~~ utilization and expenditure dataset
